# Exploring the Potential Mechanism of Liupao Tea Using UPLC-Q-TOF/MS and Network Pharmacology

**DOI:** 10.3390/ph18030294

**Published:** 2025-02-21

**Authors:** Fang Jia, Qi Yang, Lihao Yao, Yunfei Liu, Jiagang Deng, Jing Leng, Lili Fan, Erwei Hao

**Affiliations:** 1 School of Pharmacy, Guangxi University of Chinese Medicine, Nanning 530200, China; jiafang2022@stu.gxtcmu.edu.cn (F.J.); fanli228@163.com (L.F.); 2 Guangxi Key Laboratory of Efficacy Study on Chinese Materia Medica, Guangxi University of Chinese Medicine, Nanning 530200, China; 3 Engineering Research Center of Reutilization of Traditional Chinese Medicine Resources, Guangxi University of Chinese Medicine, Nanning 530200, China; 4 Guangxi Key Laboratory of TCM Formulas Theory and Transformation for Damp Diseases, Guangxi University of Chinese Medicine, Nanning 530200, China; 5 Guangxi Key Lab High Incidence Infect Dis Integrat, Guangxi University of Chinese Medicine, Nanning 530200, China

**Keywords:** Liupao tea, UPLC-Q-TOF/MS, network pharmacology, molecular docking, gastrointestinal motility disorder

## Abstract

**Background:** Gastrointestinal motility disorder (GMD) is a common condition characterized by dysfunction or degeneration of the myenteric plexus in specific segments of the gastrointestinal tract. Liupao tea (LPT) is a post-fermented tea that is rich in various secondary metabolites and has demonstrated a range of pharmacological effects, including lipid-lowering properties, antioxidant activity, and modulation of the gut microbiota. However, the underlying mechanisms by which LPT improves GMD remain poorly understood. **Methods:** Blood was collected after gavage of LPT extract in SD rats. The active components in the aqueous extract of LPT and its serum were analyzed using ultra-high-performance liquid chromatography quadrupole-time-of-flight mass spectrometry (UPLC-Q-TOF/MS). The targets of LPT in the treatment of GMD were predicted by network pharmacology and molecular docking. **Results:** 65 compounds were identified in the water extract of LPT, including flavonoids, phenolic acids, alkaloids, and amino acids. In rats treated with LPT, 14 prototype compounds and 6 metabolites were detected in serum. Network pharmacology and molecular docking analyses revealed 298 common targets between LPT and GMD, including IL-6, AKT1, and TP53. Functional enrichment analysis suggested that LPT may improve GMD through the regulation of immune, inflammatory, and cytokine signaling pathways. Molecular docking further indicated that the primary bioactive components of LPT exhibit a strong affinity for IL-6, AKT1, and TP53. **Conclusions:** These findings provide new insights into the bioactive components, molecular targets, and mechanisms of LPT, suggesting its potential as a therapeutic strategy for gastrointestinal motility disorders.

## 1. Introduction

Gastrointestinal motility disorder (GMD) is a common condition characterized by dysfunction or absence of the enteric nervous system in specific segments of the gastrointestinal tract [1]. When the intestine loses its ability to coordinate muscle contractions, motility disturbances arise, leading to various clinical symptoms. These symptoms primarily include bloating, abdominal pain, nausea, poor appetite, and fatigue [2]. Treatment strategies typically involve dietary modifications, vitamin and nutritional supplementation, anti-nausea medications, and promotility agents such as metoclopramide, domperidone, and macrolide antibiotics to enhance gastric emptying [3]. However, prolonged use can produce adverse drug reactions.

Tea is traditionally categorized into five types based on its level of fermentation: unfermented tea (green tea), lightly fermented tea (white and yellow teas), semi-fermented tea (oolong tea), fully fermented tea (black tea), and post-fermented tea (dark tea). LPT, a variety of dark tea, is named after its place of origin—Liubao Town in Cangwu County, Guangxi. With a history spanning over 1000 years, LPT was recognized as one of China’s 24 famous teas during the Qing Dynasty [4]. Recent studies have highlighted the various pharmacological properties of LPT, including its ability to lower blood lipids and glucose, provide antioxidant effects, modulate the gut microbiota, and inhibit tumor growth [5,6,7,8,9,10]. Chemical analyses reveal [8] that LPT contains several bioactive compounds, particularly tea polyphenols, which possess antioxidant, anti-inflammatory, and hepatoprotective properties, as well as caffeine, known for its mild stimulating effects that promote gastrointestinal motility, particularly by aiding gastric emptying. Based on these findings, we propose that the polyphenol-rich extract of LPT may enhance gastrointestinal function by exerting anti-inflammatory effects and stimulating smooth muscle activity in the intestines, potentially benefiting individuals with GMD.

Therefore, this study employs ultra-high-performance liquid chromatography coupled with quadrupole time-of-flight tandem mass spectrometry (UPLC-Q-TOF-MS/MS) to conduct a qualitative analysis of the chemical composition of the aqueous extract of LPT, as well as the serum components absorbed into the bloodstream. Additionally, network pharmacology and molecular docking techniques are applied to investigate the mechanisms by which LPT may improve GMD. The findings are expected to demonstrate the prokinetic effects of LPT on the gastrointestinal system, thereby providing a scientific basis for its pharmacological mechanisms.

## 2. Results

### 2.1. Total Ion Chromatogram Collection Based on UPLC-Q-TOF-MS/MS

**Figure 1:**
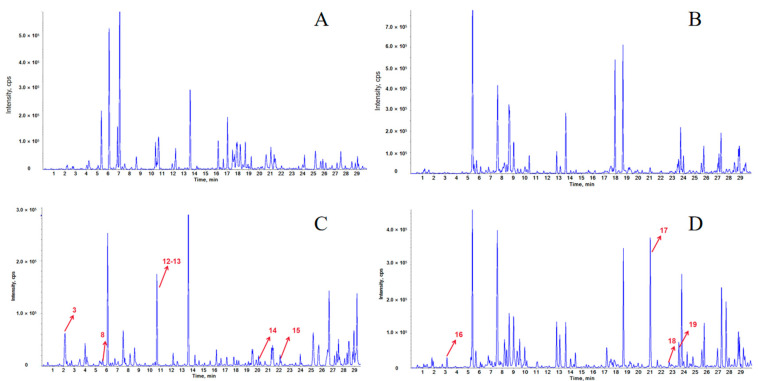
Total ion chromatograms (TIC) of serum samples based on UPLC-Q-TOF/MS. TIC of blank serum samples in ESI- mode (**A**), TIC of blank serum samples in ESI+ mode (**B**), TIC of LPT drug-containing serum samples in ESI- mode (**C**), and TIC of LPT drug-containing serum samples in ESI+ mode (**D**).

Using the established method for analyzing the blood components of LPT, data were collected from both blank serum and LPT-administered serum in positive and negative ion modes. The total ion chromatograms were obtained, as shown in Figure 1.

### 2.2. Active Components of LPT Based on UPLC-Q-TOF-MS/MS

Structural identification of compounds with available reference standards was performed by comparing retention times and secondary mass spectra. The fragmentation patterns of these compounds were further validated by their fragment ions. Using this method, 65 compounds were identified in the aqueous extract of LPT (Supplementary Material S1), including flavonoids, phenolic acids, alkaloids, amino acids, and other compounds. Mass spectrometric data from the LPT aqueous extract, serum containing LPT, and control serum samples were compared to identify blood-absorbed components. A compound was classified as a prototype of LPT absorbed into the bloodstream if it was present in both the LPT aqueous extract and the serum containing LPT, but absent in the control serum. A compound was identified as a metabolite of LPT if it was found exclusively in the serum containing LPT and not in the aqueous extract or control serum [11]. In this study, 20 blood-absorbed components were detected in the serum samples, including 14 prototype compounds and 6 metabolites, as summarized in Table 1.

### 2.3. Network Pharmacology and Molecular Docking Experimental Results

#### 2.3.1. Core Targets of LPT’s Active Compounds

The Traditional Chinese Medicine System Pharmacology (TCMSP; https://old.tcmsp-e.com/tcmsp.php, accessed on 24 March 2024) and TargetNet (http://targetnet.scbdd.com/calcnet/index/, accessed on 24 March 2024) databases were searched for the targets of active ingredients in LPT. After screening and refinement, 803 potential targets associated with these active ingredients in LPT were ultimately identified.

#### 2.3.2. Targets of Gastrointestinal Motility Disorder

Genes associated with gastrointestinal motility dysfunction were retrieved from the OMIM, GeneCards, and TTD databases. Targets from the GeneCards database with relevance scores equal to or greater than the average score were selected. The gastrointestinal motility disorder targets from all three databases were then merged and de-duplicated, yielding a total of 3093 disease-related targets.

#### 2.3.3. Venn Diagram and PPI Network

**Figure 2:**
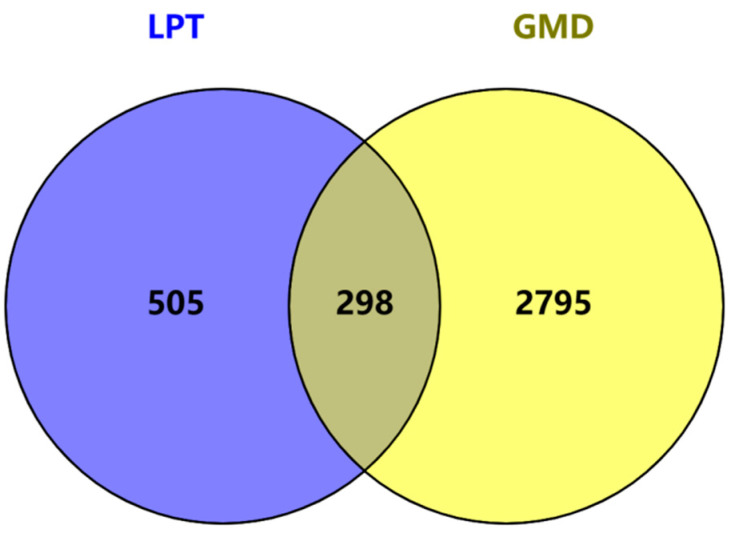
Venn diagram of active ingredients of LPT and targets related to GMD.

**Figure 3:**
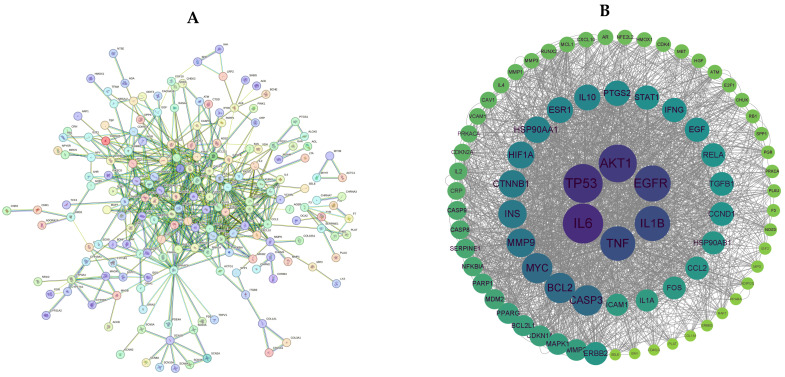
Network diagram of PPI proteins (**A**), and interaction network of key target (**B**).

The active compound targets of LPT and the targets associated with GMD were imported into Venny 2.1 (https://bioinfogp.cnb.csic.es/, accessed on 15 April 2024) to generate a Venn diagram (Figure 2) and identify the intersecting targets. A total of 298 intersecting targets were identified, which represent potential pathways through which LPT may ameliorate gastrointestinal dysfunction. These 298 targets were subsequently imported into the STRING online database to construct a protein–protein interaction (PPI) network, as shown in Figure 3A. Topological analysis was then conducted using Cytoscape 3.9.1 software. The core targets were selected based on their degree values. The degree of each gene target was visualized, with darker colors and larger node sizes indicating higher degree values, as shown in Figure 3B. The top six targets, ranked by degree, for LPT’s potential effects on improving gastrointestinal motility disorder were IL-6, TP53, AKT1, EGFR, IL-1β, and TNF.

**Figure 4:**
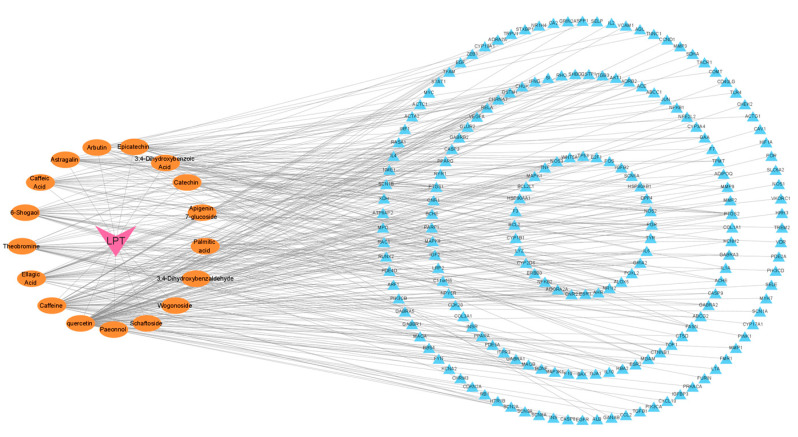
The component–targe network.

A ‘compound–target’ network was constructed using Cytoscape 3.9.1 to elucidate the interactions between the active compounds of LPT and the 298 intersection targets associated with gastrointestinal motility disorder (Figure 4). In this network, orange circular nodes represent the active compounds of LPT, pink triangular nodes indicate LPT itself, and blue triangular nodes denote disease-related intersection targets. The network reveals that LPT exerts its therapeutic effects through multiple components acting on various targets. Among these compounds, quercetin, caffeine, and ellagic acid interact with the greatest number of targets, suggesting that they may play a key role in the therapeutic effects of LPT.

#### 2.3.4. GO Functional and Module Analysis, KEGG Enrichment Pathway Analysis

**Figure 5:**
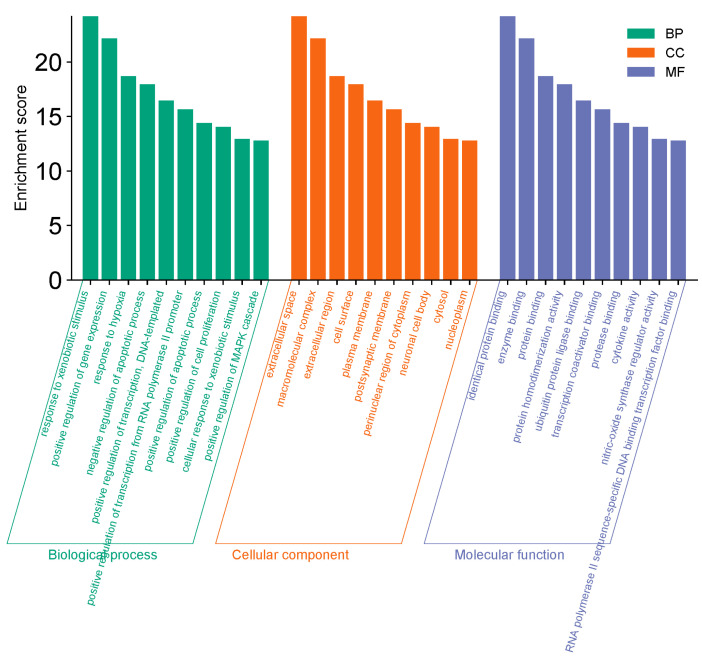
Analyzing the GO functional enrichment in LPT (top 10 for each BP/CC/MF).

A total of 298 potential targets were imported into the DAVID and Metascape databases for functional enrichment analysis based on the Gene Ontology (GO) database (http://geneontology.org/, accessed on 16 April 2024). A significance threshold of *p* ≤ 0.01 was applied for the selection of 374 biological process (BP) entries, 52 cellular component (CC) entries, and 74 molecular function (MF) entries. The BP, CC, and MF entries are presented in descending order of count values (Figure 5). The results indicate that the primary mechanisms through which LPT affects GMD include responses to xenobiotic stimuli, positive regulation of gene expression, and negative regulation of the apoptotic process.

**Figure 6:**
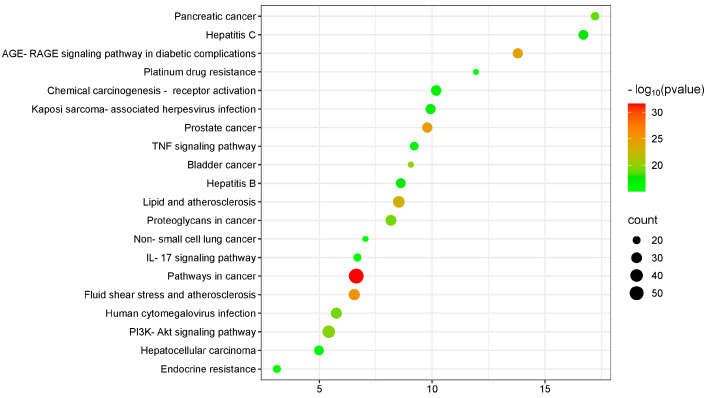
Enrichment analysis of KEGG pathway (top 20).

In the KEGG pathway enrichment analysis, a significance threshold of *p* < 0.01 was applied, identifying 137 signaling pathways. The top 20 pathways were visualized in a scatter plot (Figure 6). The results reveal that the potential target pathways are involved in several biologically relevant processes, with pathways related to malignancy and inflammation showing the strongest associations. Among the inflammation-related pathways, the “TNF signaling pathway”, “PI3K-Akt signaling pathway” and “Interleukin-17 signaling pathway” were the most enriched.

#### 2.3.5. Verification with Molecular Docking

**Figure 7:**
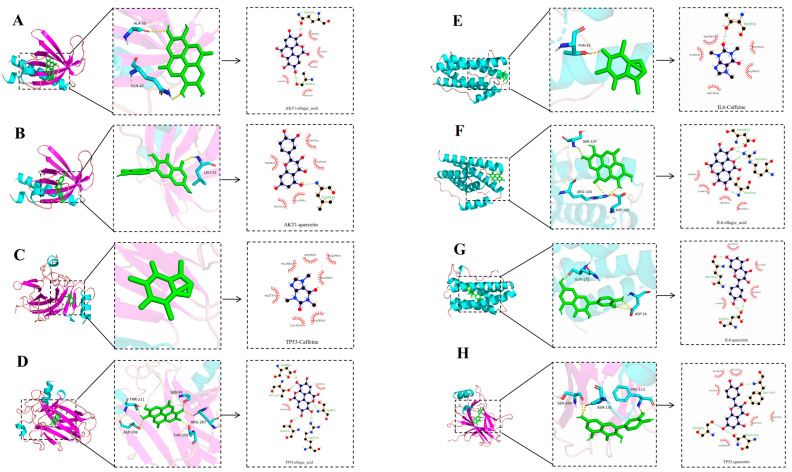
Visual analysis of molecular docking. AKT1-ellagic acid (**A**); AKT1-quercetin (**B**); TP53-caffeine (**C**); TP53-ellagic acid (**D**); IL-6-caffeine (**E**); IL-6-ellagic acid (**F**); IL-6-quercetin (**G**); TP53-quercetin (**H**).

The key active compounds of LPT, including quercetin, caffeine, and ellagic acid, were selected as ligands for docking with the key targets IL-6, AKT1, and TP53, which were identified as the top-ranked targets according to their degree values. The binding energy was used to evaluate the affinity between the active compounds and their respective targets. A lower binding energy indicates a greater release of energy, enhancing the likelihood of a successful docking interaction [11]. As detailed in Table 2, all binding energies are negative, indicating thermodynamically favorable binding between the drug molecules and the proteins. Among these, ellagic acid exhibits the strongest binding with IL-6, with a binding energy of −7.1 kcal/mol, suggesting it may be a promising candidate molecule with high target activity. The molecular docking models of key active ingredients and core targets, with binding energies lower than −5 kcal/mol, were generated using PyMOL, as shown in Figure 7.

## 3. Discussion

Gastrointestinal dysfunction is strongly influenced by lifestyle, genetic factors, and environmental conditions [12]. Studies have shown that gastrointestinal dysfunction is a common postoperative complication during the perioperative period, which directly affects patients’ postoperative recovery. The rapid recovery of gastrointestinal function is crucial. The longer gastrointestinal suppression persists, the more likely gastrointestinal motility becomes impaired or even absent, leading to increased gas and fluid accumulation in the gut lumen. This can result in intestinal dilation and may lead to severe complications such as adhesive intestinal obstruction, nutritional disorders, poor wound healing, dysbiosis, and multi-organ failure [13]. Treatment strategies typically involve dietary modifications, vitamin and nutritional supplementation, anti-nausea medications, and promotility agents such as metoclopramide, domperidone, and macrolide antibiotics to enhance gastric emptying. In traditional Chinese medicine (TCM), treatment for gastrointestinal dysfunction primarily involves oral herbal therapies, acupuncture, acupoint application, and herbal enemas [2].

China has a long history of using tea for therapeutic purposes. The *Compendium of Materia Medica Supplement* (1765) [4] records that “Pu-erh tea… relieves greasiness, detoxifies the effects of beef and mutton, and should be avoided by those with deficiency. It is bitter and astringent, promotes phlegm expulsion, and clears the intestines”. Similarly, the *Compendium of Materia Medica* (1851) states that “Pu-erh tea aids digestion, relieves stagnation, and alleviates dysentery”. In addition, Anhua tea is recognized for its milder digestive benefits. The *Compendium of Materia Medica Supplement* further notes that “Anhua tea... has a dark color, a bitter taste with a hint of sweetness. It clears the mind, harmonizes the stomach, and warms the body”. It is also known to relieve belching, promote digestion, and dispel cold. LPT, like Pu-erh and Anhua tea, belongs to the category of black tea. Based on these historical references, it is reasonable to speculate that LPT may also have regulatory effects on the gastrointestinal system. However, it should be noted that while historical texts highlight the digestive benefits of tea, modern research still requires further scientific investigation and clinical trials to substantiate these claims. This study employed UPLC-Q-TOF-MS/MS to analyze the chemical constituents of both the water extract of LPT and the serum samples from rats administered LPT extract. By examining chromatographic retention times, precise molecular weights, and characteristic fragment ions, 65 compounds were identified in the water extract. In the drug-containing serum, 20 bioavailable compounds were identified, comprising 14 prototype compounds and 6 metabolites, primarily consisting of flavonoids, alkaloids, and organic acids. Quercetin, a flavonoid commonly found in plants, has been shown to promote gastrointestinal motility and improve gut movement in constipated rats [14]. A study by Saccon et al. [15]. examined the effects of a long-term combination of the anti-aging drug dasatinib and quercetin on gut aging, inflammation, and microbiome composition in aged mice. The results indicated that this combination reduced gut aging, alleviated inflammation, and improved metabolic function in elderly individuals. GMD is closely associated with inflammation. Ellagic acid, a natural antioxidant, exhibits anti-inflammatory, bacteriostatic, and antitumor properties. Research by Hooi Poay T et al. [16]. found that ellagic acid inhibited inflammatory mediator-induced colon cancer in rats, significantly reducing the levels of IL-6, NF-κB, COX-2, and TNF-α in colon cancer cells. Additionally, ellagic acid demonstrated anti-inflammatory and chemoprotective effects, inhibiting cell proliferation and inducing apoptosis in HCT-15 colon cancer cells via the Akt signaling pathway. Caffeine [17], one of the most abundant active compounds in tea, is well-known for its stimulating effect on gastric acid secretion. Recent studies have shown that when combined with other compounds, it acts synergistically through the AMPKα-LXRα/SREBP-1c pathway to regulate lipid metabolism in high-fat-diet-induced obese mice. Furthermore, caffeine stimulates gastric parietal cells to secrete gastric acid, which promotes the faster transit of food residue into the intestine, thereby accelerating digestion.

To further explore the mechanisms underlying the effects of LPT on GMD, network pharmacology and molecular docking approaches were utilized. The results indicate that the primary active compounds of LPT in the treatment of these disorders are quercetin, caffeine, and ellagic acid. The key targets identified include IL-6, AKT1, TP53, and EGFR. Gene Ontology (GO) enrichment analysis highlights the involvement of processes such as responses to exogenous stimuli, positive regulation of gene expression, hypoxic responses, and negative regulation of apoptosis. KEGG pathway enrichment analysis revealed that the potential target pathways are primarily involved in biologically significant processes, with the most prominent associations observed in pathways related to malignancy and inflammation. Among the inflammation-related pathways, the “TNF signaling pathway”, ”PI3K-Akt signaling pathway” and “Interleukin-17 signaling pathway” were the most significantly enriched. Molecular docking results demonstrate strong binding between the core targets and their corresponding active components. IL-6, an important cytokine primarily secreted by mononuclear macrophages, plays a role in the differentiation of various cells in the body [18]. In a study by Pohjanen VM et al. [19], it was found that IL-6 is highly expressed in both gastric cancer tissues and the serum of patients with gastric cancer, suggesting that IL-6 may be involved in the malignant transformation of the gastric mucosa. Furthermore, other studies have shown that the IL-6 level is significantly elevated in the serum of patients with gastritis and peptic ulcers, and it is also expressed at higher levels in gastric cancer patients [20]. Kim et al. [21] found that 2’-fucosyllactose (2’-FL) and 3-fucosyllactose (3-FL) significantly ameliorate intestinal barrier dysfunction induced by interleukin-6 (IL-6). These oligosaccharides enhance intestinal mucosal integrity by modulating gut microbiota composition and function. In vitro experiments showed that 2’-FL and 3-FL protect intestinal epithelial cells by inhibiting proinflammatory cytokines (including IL-6) and reducing inflammatory cell infiltration. Song et al. [22] found that electroacupuncture accelerates delayed intestinal transit in postoperative ileus by suppressing M1-like muscularis macrophages and IL-6 secretion. AKT1, a serine/threonine protein kinase, is involved in a variety of biological processes, including metabolism, proliferation, cell survival, and growth. Research has shown that AKT1 can enhance cell invasion and migration, leading to the malignant transformation of the human gastric epithelial cell line GES-1 [23]. Lin et al. [24] investigated the anti-colorectal cancer mechanism of Wei-Tong-Xin Ethanol Extract (WTXE) using network pharmacology and experimental validation. Cell experiments have shown that WTXE treatment significantly reduced the expression of key proteins in the PI3K/AKT pathway, such as p-AKT and mTOR. Animal studies further demonstrated that WTXE induced apoptosis in tumor cells by inhibiting the PI3K/AKT signaling pathway. The Wuda Granule (WDG) [25] is a traditional Chinese medicine compound used to treat GMD, and recent studies have explored its mechanisms and pharmacological effects. Network pharmacology and molecular docking indicate that the key active components in WDG may exert therapeutic effects by targeting the PI3K-Akt and Rap1 signaling pathways, regulating targets like AKT and PIK3CA to modulate inflammation and improve gastrointestinal motility. Clinical trials have shown that WDG significantly increases the contraction amplitude and motility index of the gastric antrum, duodenum, and jejunum, aiding in the faster recovery of normal gastrointestinal function. TP53 [26] is a tumor suppressor gene located on chromosome 17p13, also known as an oncogene. The p53 protein encoded by the TP53 gene plays a critical role in regulating cell division, proliferation, apoptosis, and DNA repair. Studies have reported that TP53 gene mutations are closely associated with cancer subtypes, especially solid tumors. Gu et al. [27] investigated the impact of TP53 mutations on adjuvant chemotherapy and immunotherapy in gastric cancer (GC). TP53 mutations occur in about 50% of GC cases and up to 70% of metastatic tumors. The study found that GC patients with TP53 mutations often exhibit poor responses to traditional adjuvant chemotherapy due to compromised DNA repair, cell cycle regulation, and apoptosis, leading to chemoresistance. Zhao et al. [28] induced an infantile anorexia (IFA) model in juvenile rats using a high-fat diet to investigate the effects of Shenqu Xiaoshi Oral Solution (SQXSOS) on digestive function and gastrointestinal microbiota. Network pharmacology analysis identified AKT1, TP53, JUN, and MAPK1 as the key target genes for SQXSOS in treating IFA. Experimental data have shown that the mRNA transcription levels of these genes in the gastric antrum of IFA rats were significantly higher than those in the control group. However, treatment with SQXSOS (3.78 and 7.56 mL/kg/day) significantly reduced these levels by 41.1–77.3% (*p* < 0.05). The TNF signaling pathway and IL-17 signaling pathway play crucial roles in the inflammatory response. TNF-α [29] is a key inflammatory mediator that not only induces neutrophil infiltration and inflammation but also promotes intestinal epithelial cell apoptosis and increases intestinal epithelial permeability, contributing to gastrointestinal dysfunction. IL-17 is essential for regulating inflammation, as it induces the release of proinflammatory cytokines such as IL-6, IL-1β, and TNF-α, which mediate the body’s defense response. The expression levels of IL-17A and IL-17F are significantly elevated in the inflamed mucosa of patients with inflammatory bowel disease (IBD) [30,31,32].

Overall, this study provides valuable new insights into the potential therapeutic effects of LPT on GMD. These findings suggest that LPT could play a significant role in modulating the progression of GMD and may offer a novel avenue for treatment. However, to fully confirm the relevance and applicability of these results, further experimental validation is necessary. In the next phase of our research, we plan to conduct in-depth animal experiments to assess the therapeutic efficacy of LPT in vivo. Additionally, we will perform mechanistic studies to better understand the underlying molecular pathways involved. Moreover, we will further validate the molecular docking results to ensure that the predicted interactions between LPT and target proteins align with biological outcomes.

## 4. Materials and Methods

### 4.1. Materials

#### 4.1.1. Drugs

LPT was provided by Guangxi Wuzhou Zhongcha Tea Co., Ltd. (Wuzhou, China).

#### 4.1.2. Animals

Adult male Sprague Dawley (SD) rats, weighing between 180 and 220 g, were purchased from Hunan Sleck Jingda Experimental Animal Co., Ltd. (Changsha, China). The animals were housed in a pathogen-free environment, maintained at a temperature of 25 ± 5 °C and a relative humidity of 55 ± 5%. Standard laboratory chow and water were provided ad libitum. All animal procedures were conducted in accordance with the *Guidelines for the Care and Use of Laboratory Animals*, and the study was approved by the Animal Care and Use Committee of Guangxi University of Chinese Medicine (approval number: DW20240319-039).

#### 4.1.3. Instruments and Reagents

We employed the following instruments: HDM-500 constant-temperature electric heating mantle (Jiangsu Tianyou Co., Ltd., Jintan, China); TGL-16M desktop high-speed refrigerated centrifuge (Hunan Xiangyi Co., Ltd., Changsha, China); RV-8V rotary evaporation apparatus (Ronghui Innovative Technology Co., Ltd., Huizhou, China); 100-mesh nylon filter bag (Shanghai Xinmiao Laboratory Equipment Co., Ltd., Shanghai, China); HH-S8 digital constant-temperature water bath (Jintan Medical Instrument Factory, Jintan, China); ExionLC AC liquid chromatography–mass spectrometry system (Agilent Technologies Co., Ltd., Santa Clara, CA, USA); X500R QTOF mass spectrometer (Agilent Technologies Co., Ltd., Santa Clara, CA, USA); KQ-500DE ultrasonic cleaner (Kunshan Ultrasonic Instrument Co., Ltd., Kunshan, China). We employed the following reagents: acetonitrile, formic acid (Thermo Fisher Scientific, Waltham, MA, USA); methanol (Chengdu Kolon Chemicals Co., Ltd., Chengdu, China); ellagic acid, D-(-)-quinic acid, gallic acid, protocatechuic aldehyde, (-)-epigallocatechin, catechin, epicatechin, epicatechin gallate (Shanghai Yuan Ye Bio-Technology Co., Ltd., Shanghai, China), all analytical grade; chaferoside, ellagic acid, rutin, delphinidin, hyperoside, genistein, quercetin, hesperidin, L-valine, and caffeine (Chengdu Manster Biotech Co., Ltd., Chengdu, China), all analytical grade.

### 4.2. Preparation of the Aqueous Extract of LPT

LPT was refined using established procedures with slight adjustments, as outlined in [7]. An accurate amount of LPT was weighed and mixed with distilled water at a 1:10 (tea leaves: purified water, g/mL) ratio. The mixture was boiled and extracted for 10 min, and then filtered. The residue was re-extracted with purified water at a 1:8 (tea leaves: purified water, g/mL) ratio, boiled for an additional 10 min, and filtered again. The two filtrates were combined, and the solvent was removed under reduced pressure at 55 °C using a rotary evaporator. The resulting concentrate was then freeze-dried to obtain the final extract.

### 4.3. Animal Dosing and Sample Collection

After a 1-week acclimatization period, healthy male Sprague Dawley (SD) rats were randomly assigned to two groups: a control group and a treatment group, with 6 rats in each group. Prior to the experiment, the rats were fasted for 12 h and allowed free access to water. The rats in the treatment group received an intragastric administration of 4 g/kg body weight of LPT freeze-dried powder dissolved in purified water, while the control group was given an equal volume of physiological saline. Blood samples were collected from the abdominal aorta of anesthetized rats in both groups two hours after treatment. The blood samples were allowed to stand at room temperature for 1 h, followed by centrifugation at 3000 rpm for 10 min at 4 °C. The resulting supernatant was collected as serum and stored at −80 °C for subsequent analysis. All animal experiments were performed according to institutional guidelines for ethical animal studies.

### 4.4. Sample Preparation

#### 4.4.1. Preparation of Test Solution

An accurately weighed amount of 10 mg of LPT powder (prepared using method Section 4.2) was placed into a 10 mL Erlenmeyer flask. To this, 10 mL of 50% aqueous methanol was added, and the mixture was sealed and shaken thoroughly. The solution was then sonicated for 30 min. After filtration through a 0.22 µm membrane filter, the filtrate was collected as the test solution.

#### 4.4.2. Preparation of Standard Solution

Standard substances (10 mg each) were accurately weighed and dissolved in 10 mL of 50% aqueous methanol. The solution was sonicated and mixed thoroughly to prepare the stock solutions. After filtration through a 0.22 µm membrane filter, the resulting solution was utilized for analysis.

#### 4.4.3. Preparation of Serum Samples

The serum was prepared following the method outlined in a previous report [33]. After thawing the blank and drug-containing sera at 4 °C, 1 mL of each serum sample was aliquoted. The serum samples were then mixed with three times their volume of mass spectrometry-grade methanol to precipitate proteins. After centrifugation at 15,000 rpm for 5 min at 4 °C, the supernatant was transferred to new centrifuge tubes for subsequent concentration and drying. The residue was redissolved in 80 μL of 80% aqueous methanol, mixed, and centrifuged at 12,000 rpm for 5 min at 4 °C. The supernatant was then collected for UPLC-Q-TOF-MS analysis.

### 4.5. Chromatographic and Mass Spectrometric Conditions

#### 4.5.1. Chromatographic Conditions

Chromatographic separation was performed on a Waters ACQUITY UPLC HSS T3 C18 column (1.8 µm, 2.1 × 100 mm). The mobile phase comprised A: 0.1% formic acid in water and B: acetonitrile. A gradient elution was applied as follows: 0–30 min, from 5% to 95% B. The flow rate was maintained at 0.4 mL/min, with an injection volume of 3 µL. The column temperature was kept constant at 40 °C throughout the analysis.

#### 4.5.2. Mass Spectrometric Conditions

Mass spectrometry analysis was conducted using a dual-spray TurboV ion source, with ionization performed in both positive and negative ion modes. The TOF MS scan range was set from *m/z* 100 to 1500. The ion source voltages were set to −4500 V for the negative mode and 5500 V for the positive mode. The ion source temperature was maintained at 600 °C, and the gas flow rates for GS1 and GS2 were both set to 55 psi. The curtain gas pressure was kept at 35 psi. For parent ions, the collision energy was set to ±10 V, while the declustering potential (DP) was ±80 V and the collision energy (CE) for fragment ions was ±35 V.

### 4.6. Establishment of the LPT Database and Data Processing

Relevant literature on the chemical components of LPT was reviewed using databases such as China National Knowledge Infrastructure (CNKI, https://www.cnki.net/, accessed on 22 March 2024), PubMed (https://pubmed.ncbi.nlm.nih.gov/, accessed on 22 March 2024), Web of Science (https://www.webofscience.com/, accessed on 22 March 2024), and PubChem (https://pubchem.ncbi.nlm.nih.gov/, accessed on 22 March 2024). The identification and extraction of peaks, along with the correction of raw data to obtain retention times, accurate molecular weights, and MS/MS fragment ion information, were performed using the SCIEX system, PubChem, reference standards, and literature databases.

### 4.7. Network Pharmacology and Molecular Docking

#### 4.7.1. Screening of Target Genes for Active Compounds in LPT

The targets of the compounds in the LPT drug-containing serum were searched in the Traditional Chinese Medicine System Pharmacology (TCMSP; https://old.tcmsp-e.com/tcmsp.php, accessed on 24 March 2024) and TargetNet (http://targetnet.scbdd.com/calcnet/index/, accessed on 24 March 2024) databases. Through reading the literature research, we found that oral bioavailability (OB) and drug-likeness (DL) are commonly used as key indicators of the drug-like properties of bioactive molecules with potential therapeutic effects. Therefore, compounds in the LPT-containing drug serum, as well as the chemical components with OB ≥ 30% and DL ≥ 0.18% in the aqueous extract of LPT, were categorized as “active ingredients.” The chemical constituents of LPT that met these criteria were selected, and their corresponding targets were compiled to generate a list of active compound targets for LPT.

#### 4.7.2. Screening of Targets for GMD

To identify disease-related targets for GMD, we conducted a search using the keyword “gastrointestinal motility disorder” across three databases: OMIM (https://www.omim.org/, accessed on 26 March 2024), GeneCards (https://www.genecards.org/, accessed on 26 March 2024), and TTD (https://db.idrblab.net/ttd/, accessed on 26 March 2024). The results from these databases were then merged and de-duplicated to identify relevant targets associated with gastrointestinal motility disorder.

#### 4.7.3. Construction of Venn Diagram and Protein–Protein Interaction (PPI) Network

The active compound targets of LPT and those associated with GMD were imported into Venny 2.0.2 (https://bioinfogp.cnb.csic.es/tools/venny/index.html, accessed on 15 April 2024) to generate a Venn diagram. The intersections of these targets were identified as potential targets through which LPT may improve gastrointestinal dysfunction. Subsequently, these intersection targets were uploaded to the STRING database (Search Tool for the Retrieval of Interacting Genes/Proteins, https://cn.string-db.org/, accessed on 15 April 2024), where “Homo sapiens” was selected under the “Multiple Proteins” option for interaction analysis. A high interaction score threshold of 0.900 (indicating highest confidence) was applied, and isolated nodes were excluded. This approach facilitated the construction of a drug–target protein–disease protein interaction network. The data were then visualized using Cytoscape version 3.9.0 to calculate degree values and rank the targets accordingly. The core targets for LPT in improving gastrointestinal motility disorder were selected based on their degree values.

#### 4.7.4. GO Functional and Module Analysis, KEGG Pathway Enrichment Analysis

To gain a deeper understanding of the functions of the overlapping target genes, these genes were uploaded to the Database for Annotation, Visualization, and Integrated Discovery (DAVID) (https://david.ncifcrf.gov/, accessed on 16 April 2024), specifying the species as “Homo sapiens”. Functional enrichment analysis was performed on the overlapping genes using Gene Ontology (GO) and Kyoto Encyclopedia of Genes and Genomes (KEGG) pathway analyses. The results of the KEGG pathway and GO functional enrichment analyses were visualized using the online tool Microbioinfo (www.bioinformatics.com.cn, accessed on 16 April 2024).

#### 4.7.5. Molecular Docking

To further validate the primary active components of LPT based on drug–target interactions, molecular docking studies were conducted. The target network of the components was analyzed using Cytoscape software, and the components with the highest degree values were selected as docking ligands. The core target was chosen based on the PPI network, and the target protein for molecular docking was selected. The 3D structure of the docking protein was downloaded from the PDB database (https://www.rcsb.org/, accessed on 23 April 2024), saved in the “pdb” format, and imported into PyMOL 2.4.0 software to remove water molecules and ligands. Finally, the structure was imported into AutoDockTools 4.0 software for hydrogenation, charge calculation, and to set the atomic type of the protein. AutoDock Vina (https://vina.scripps.edu/, accessed on 26 April 2024) was then used to perform semi-flexible docking, identifying the binding energy and position of the LPT component.

## 5. Conclusions

This study, through UPLC-Q-TOF/MS, identified 65 chemical compounds and 20 prototype/metabolic blood components in LPT. Based on the identified components, network pharmacology and molecular docking were used to screen the effective components of LPT and clarify their underlying mechanisms in improving GMD. The mechanism by which LPT improves GMD may involve the action of quercetin, caffeine, and ellagic acid on key targets such as IL6, AKT1, and TP53, which play significant roles in signaling pathways related to malignancies and inflammation. These findings provide new insights into the bioactive components, molecular targets, and mechanisms of LPT, suggesting its potential as a therapeutic strategy for gastrointestinal motility disorders.

## Figures and Tables

**Table 1:** Identification results of active components of drug-containing plasma samples.

NO.	Rt/min	Component Name	Formula	Adduct	Precursor Mass	Found at Mass	Mass Error (ppm)	Category
1	1.12	7-methylxanthine	C_6_H_6_N_4_O_2_	[M-H]^−^	165.0418	165.0420	1.3	prototype
2	2.08	esculin hydrate	C_7_H_8_N_4_O_2_	[M-H]^−^	339.0722	339.0722	0.1	metabolite
3	2.16	theobromine	C_15_H_16_O_9_	[M-H]^−^	179.0574	179.0576	0.8	prototype
4	3.34	protocatechuic acid	C_7_H_6_O_4_	[M-H]^−^	153.0193	153.0195	1.0	metabolite
5	4.39	paeonol	C_9_H_10_O_3_	[M-H]^−^	165.0557	165.0560	1.6	prototype
6	5.10	wogonoside	C_22_H_20_O_11_	[M-H]^−^	459.0933	459.0932	−0.3	metabolite
7	5.64	apigenin 7-glucoside	C_21_H_20_O_10_	[M-H]^−^	431.0984	431.0982	−0.4	prototype
8	5.82	3,4-Dihydroxybenzaldehyde	C_7_H_6_O_3_	[M-H]^−^	137.0244	137.0245	0.6	prototype
9	5.89	schaftoside	C_26_H_28_O_14_	[M-H]^−^	563.1406	563.1405	−0.2	prototype
10	6.58	astragalin	C_21_H_20_O_11_	[M-H]^−^	447.0933	447.0935	0.6	prototype
11	7.15	caffeic acid	C_9_H_8_O_4_	[M-H]^−^	179.035	179.0351	0.7	metabolite
12	11.03	catechin	C_15_H_14_O_6_	[M-H]^−^	289.0718	289.0746	9.9	prototype
13	11.03	epicatechin	C_15_H_14_O_6_	[M-H]^−^	289.0718	289.0746	9.9	prototype
14	20.61	ganoderic acid C6	C_30_H_42_O_8_	[M-H]^−^	529.2807	529.2798	−1.7	metabolite
15	22.40	6-shogaol	C_17_H_24_O_3_	[M-H]^−^	275.1653	275.165	−0.9	prototype
16	3.03	caffeine	C_8_H_10_N_4_O_2_	[M+H]^+^	195.0877	195.0873	−2.0	prototype
17	21.85	arbutin	C_12_H_16_O_7_	[M+H]^+^	273.0969	273.0971	0.6	metabolite
18	22.92	palmitic acid	C_16_H_33_NO	[M+H]^+^	256.2635	256.2635	0.1	prototype
19	23.58	oleamide	C_18_H_35_NO	[M+H]^+^	282.2791	282.2794	0.9	prototype
20	26.01	stearic acid amide	C_18_H_37_NO	[M+H]^+^	284.2948	284.2948	0.1	prototype

**Table 2:** Interaction details of molecular docking.

Compound	Targets	Combination of Energy (kcal/mol)
Ellagic acid	TP53	−6.8
Quercetin	TP53	−6.4
Caffeine	TP53	−5.1
Ellagic acid	IL-6	−7.1
Quercetin	IL-6	−6.8
Caffeine	IL-6	−5.1
Ellagic acid	AKT1	−6.5
Quercetin	AKT1	−6.1
Caffeine	AKT1	−4.9

## Data Availability

The databases used in this study are all public databases, and citations should be noted when referenced. The databases include the following: CNKI (https://www.cnki.net/, accessed on 22 March 2024), PubMed (https://pubmed.ncbi.nlm.nih.gov/, accessed on 22 March 2024), PubChem (https://pubchem.ncbi.nlm.nih.gov/, accessed on 22 March 2024), TCMSP (https://old.tcmsp-e.com/index.php, accessed on 29 March 2024), OMIM (https://www.omim.org/, accessed on 30 March 2024), GeneCards (https://www.genecards.org/, accessed on 30 March 2024), TTD (https://db.idrblab.net/ttd/, accessed on 30 March 2024) databases, STRING database (https://cn.string-db.org/, accessed on 16 April 2024) and DAVID (https://david.ncifcrf.gov/, accessed on 16 April 2024). Additionally, the chemical component database of LPT obtained in this study will be shared as Supplementary Material (S1). The ion fragmentation information of LPT by the UPLC-Q-TOF/MS will be shared as Supplementary Material (S2).

## Supplementary Materials

The following supporting information can be downloaded at https://www.mdpi.com/article/10.3390/ph18030294/s1, Supplementary Material S1: Chemical constituents in Liupao tea by UPLC-QTOF-MS /MS. Supplementary Material S2: Ion fragment information.
