# Supplementary material for: Exploring the Potential Mechanism of Liupao Tea Using UPLC-Q-TOF/MS and Network Pharmacology
Source: Pharmaceuticals (Basel). 2025 Feb 21;18(3):294. doi: 10.3390/ph18030294 (PMC11946460; doi:10.3390/ph18030294)

Total ion flow diagram of LPT aqueous extracts （ESI+）

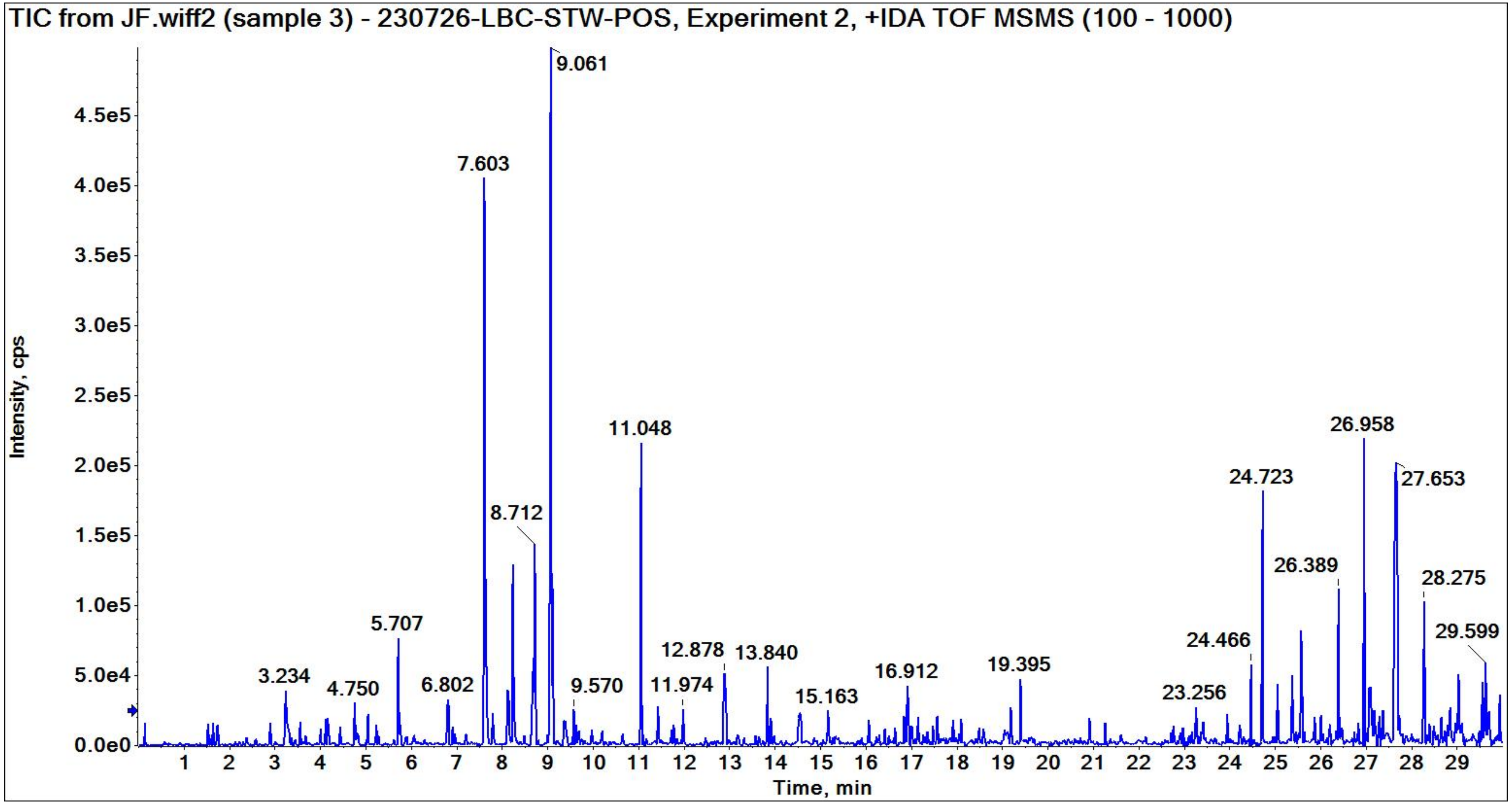

Total ion flow diagram of LPT aqueous extracts （ESI-）

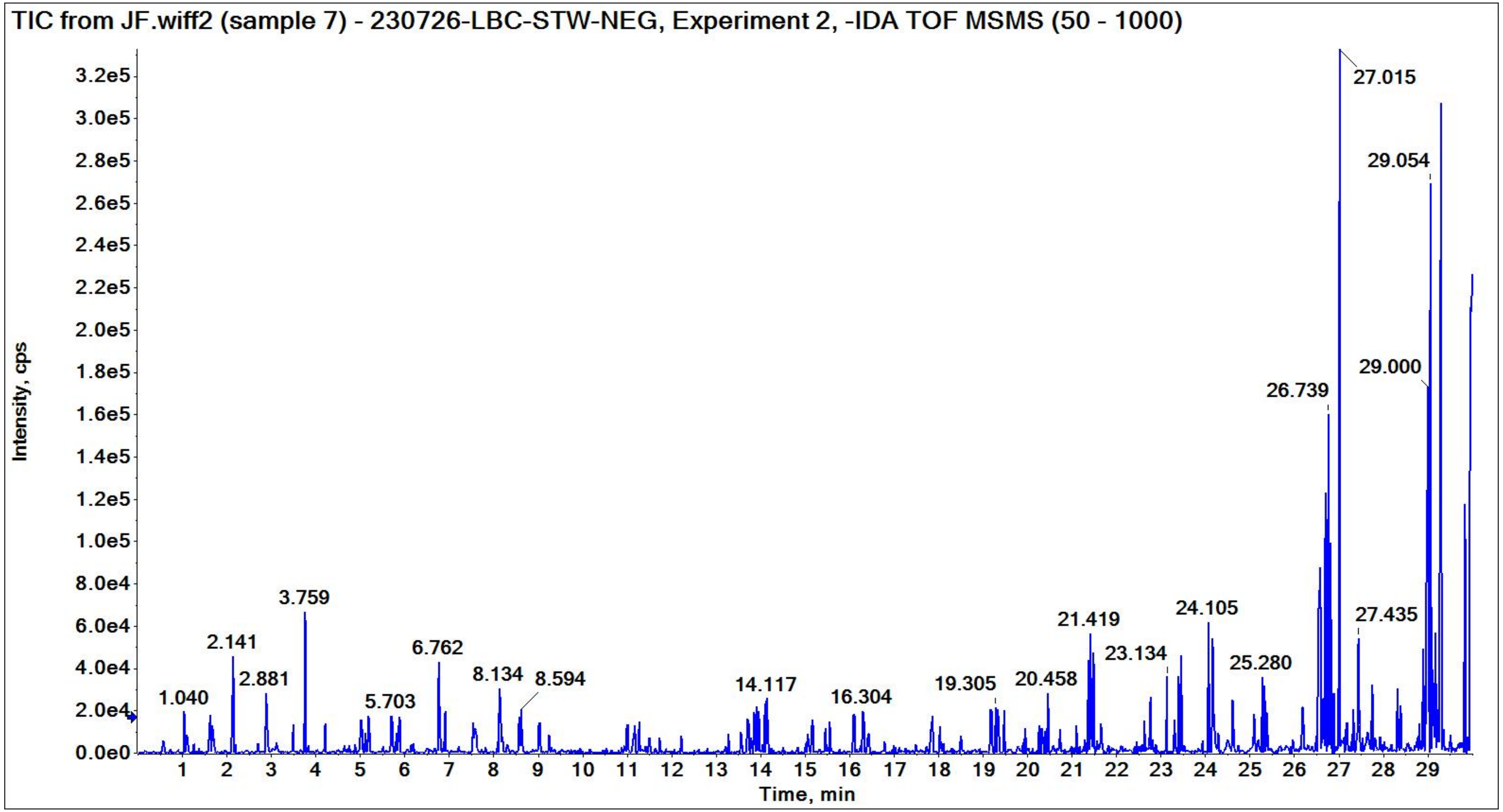

6-O-(beta-D-Xylopyranosyl)-beta-D-glucopyranose

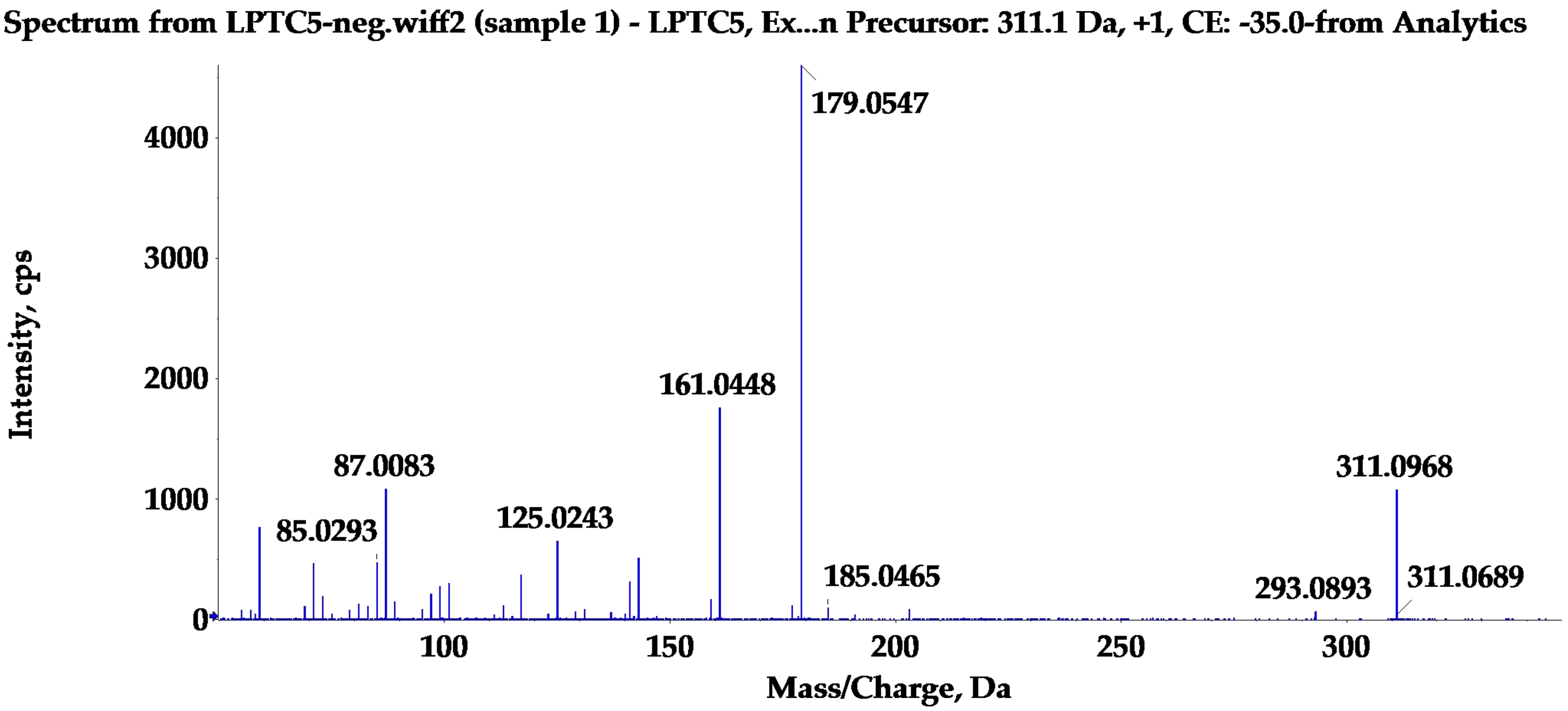

D-(-)-Quinic acid

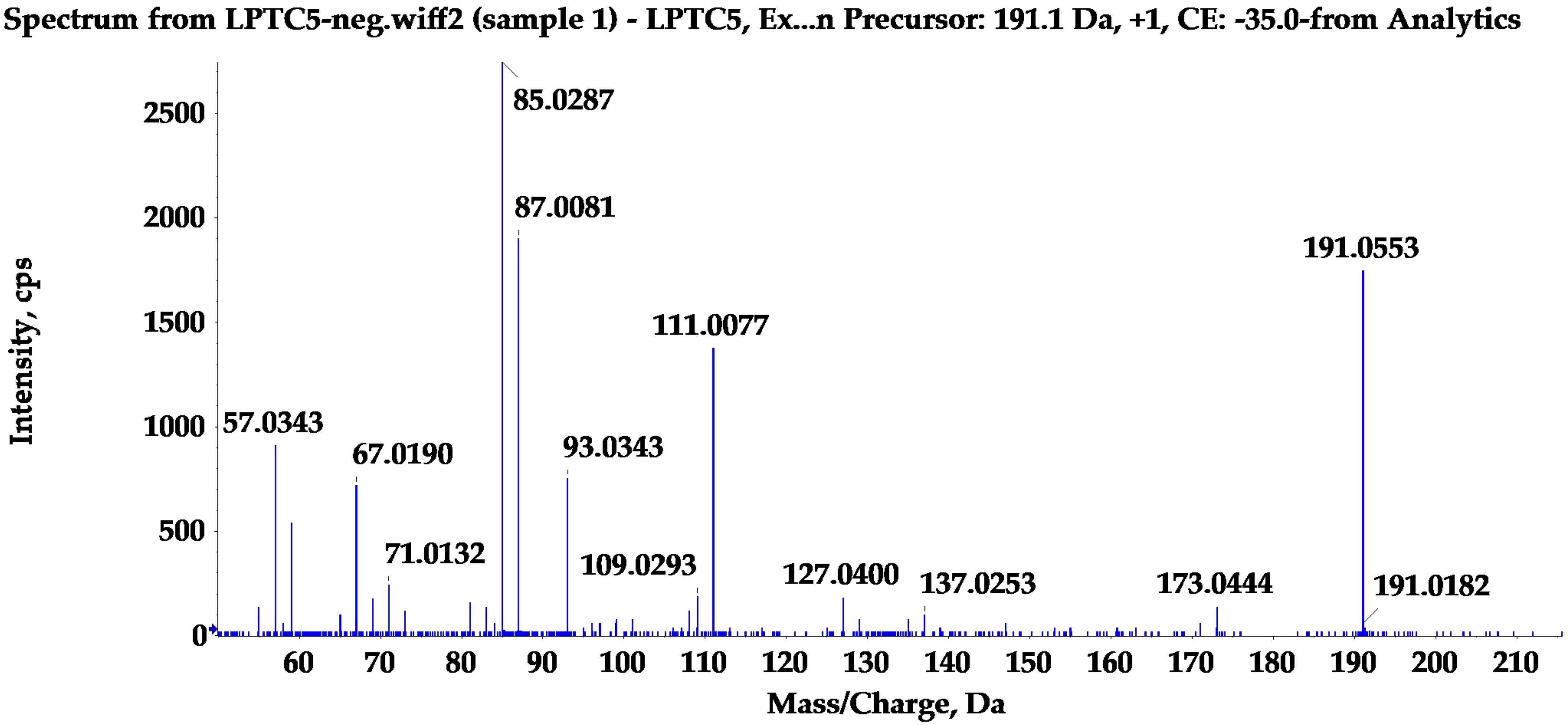

1-Galloyl-glucose

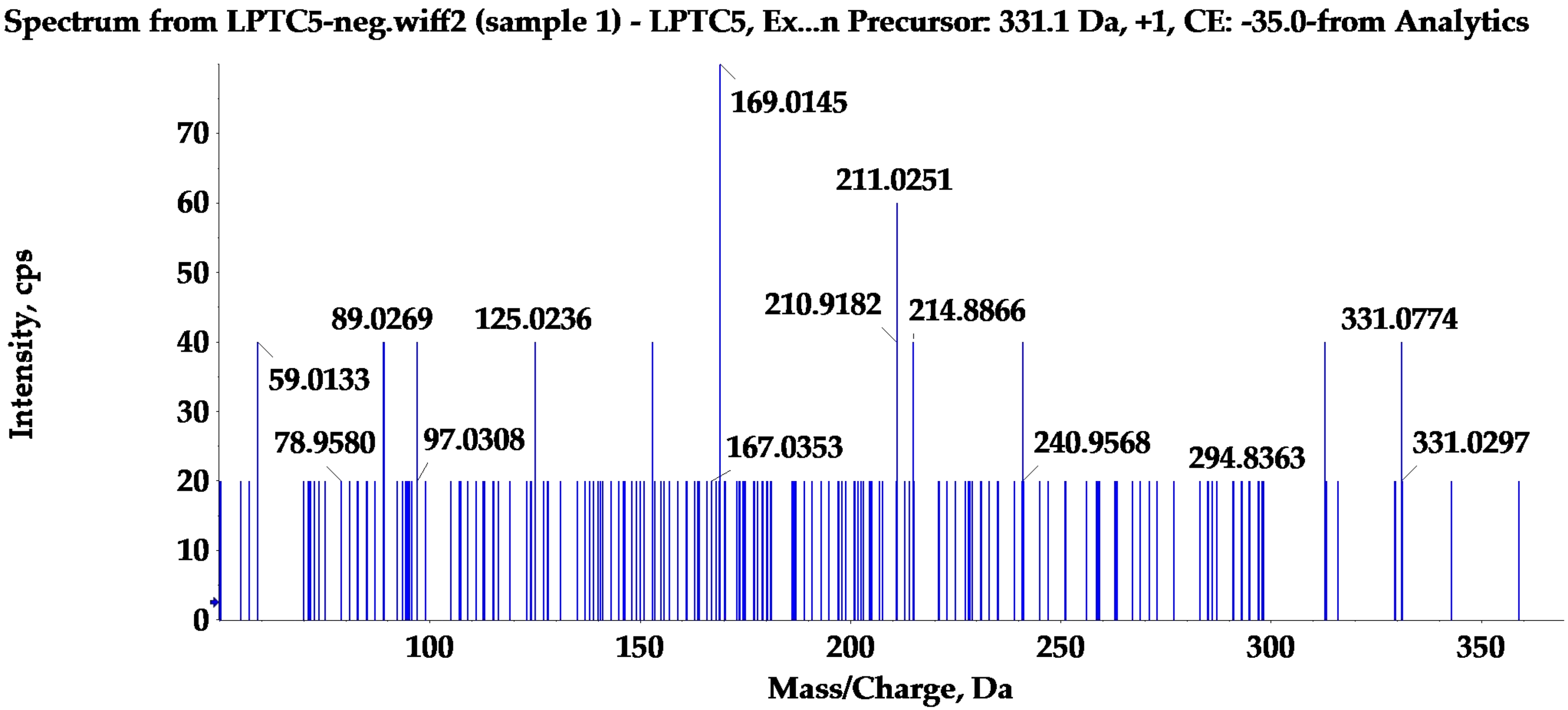

Adenine

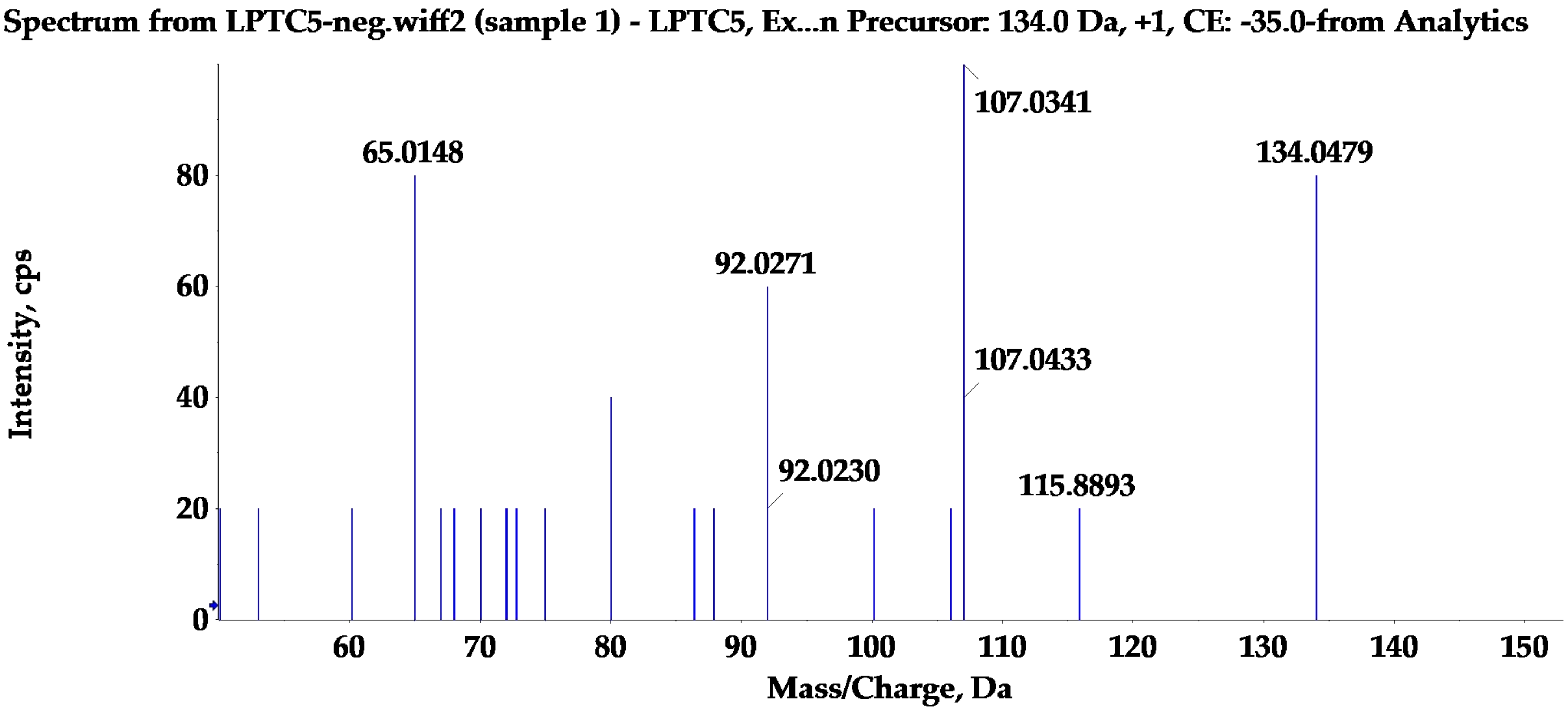

Spectrum from LPTC5-neg.wiff2 (sample 1) - LPTC5, Ex...n Precursor: 134.0 Da, +1, CE: -35.0-from Analytics
107.0341
65.0148
134.0479
92.0271
107.0433
92.0230
115.8893
Intensity, cps
0
20
40
60
80
60
70
80
90
100
110
120
130
140
150
Mass/Charge, Da

Citric Acid

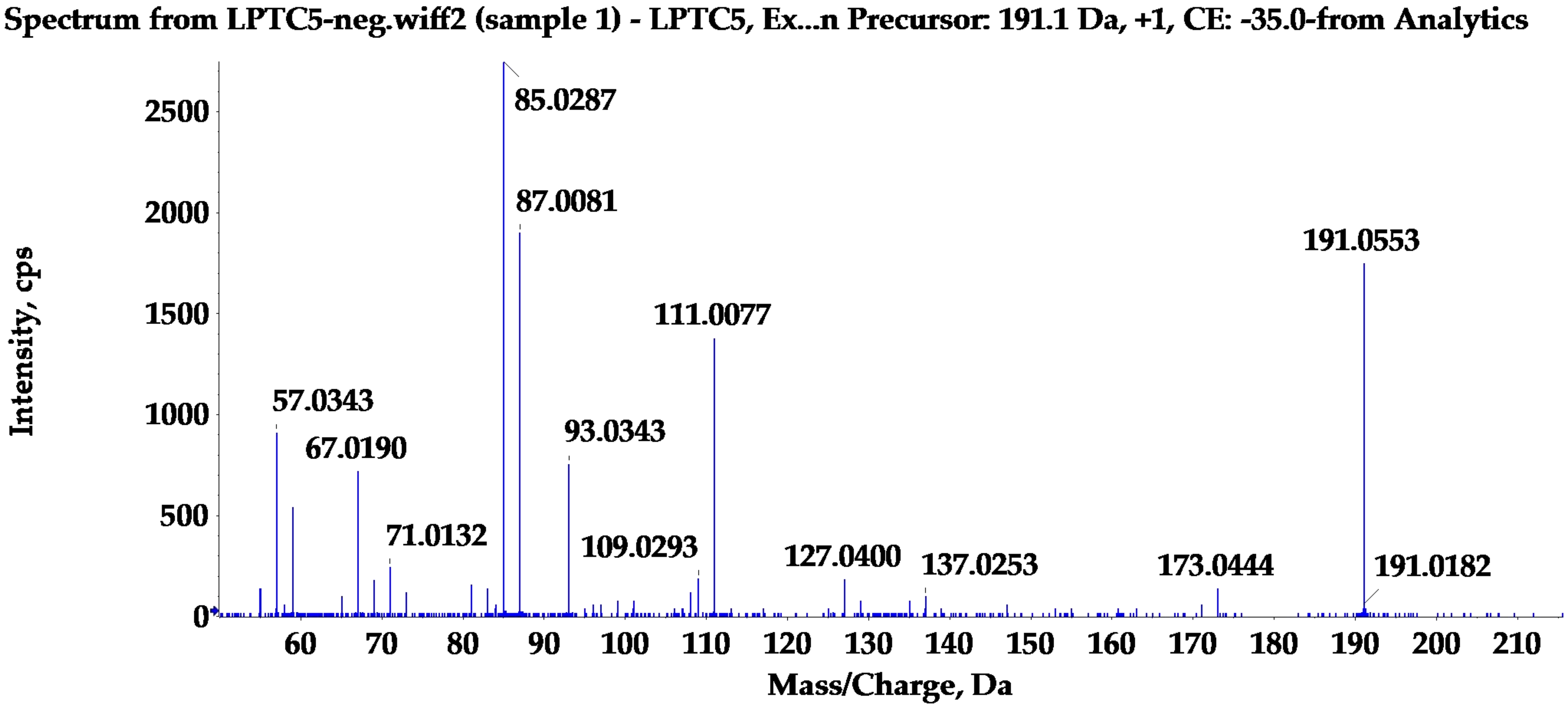

Spectrum from LPTC5-neg.wiff2 (sample 1) - LPTC5, Ex...n Precursor: 191.1 Da, +1, CE: -35.0-from Analytics
Intensity, cps
2500
2000
1500
1000
500
0
85.0287
87.0081
191.0553
111.0077
57.0343
67.0190
93.0343
71.0132
109.0293
127.0400
137.0253
173.0444
191.0182
60
70
80
90
100
110
120
130
140
150
160
170
180
190
200
210
Mass/Charge, Da

Uridine

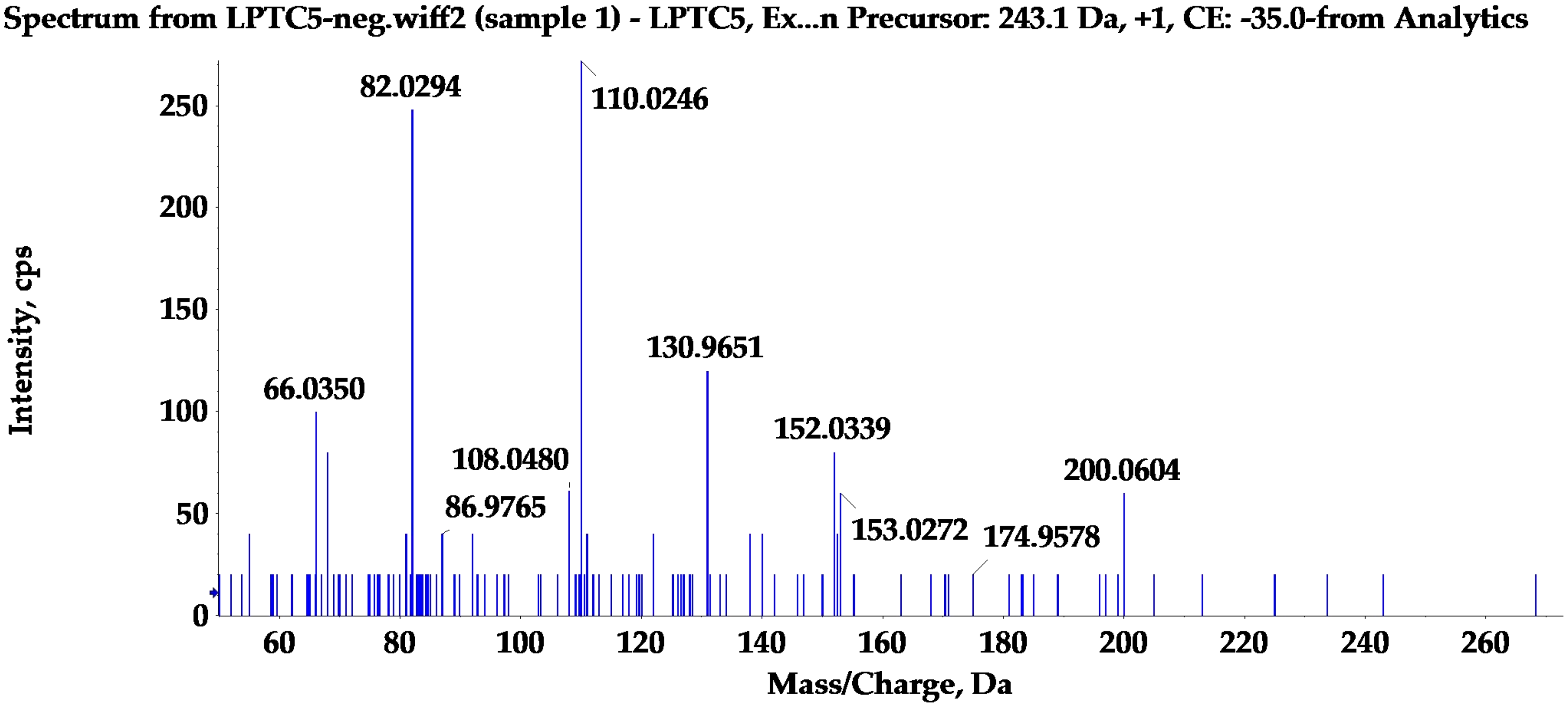
Spectrum from LPTC5-neg.wiff2 (sample 1) - LPTC5, Ex...n Precursor: 243.1 Da, +1, CE: -35.0-from Analytics
Intensity, cps
Mass/Charge, Da
82.0294
110.0246
130.9651
66.0350
152.0339
108.0480
200.0604
86.9765
153.0272
174.9578
0
50
100
150
200
250
60
80
100
120
140
160
180
200
220
240
260

Gallic acid

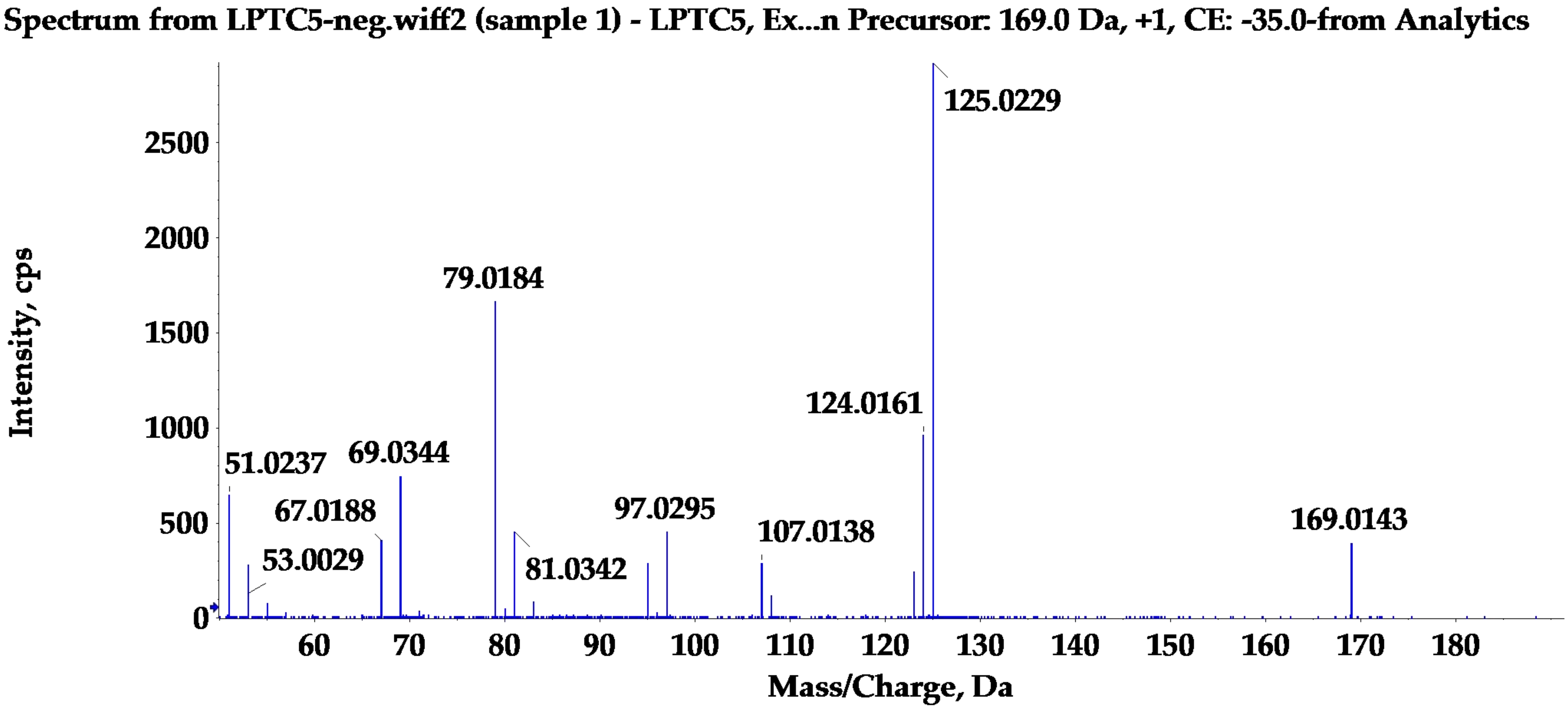

Spectrum from LPTC5-neg.wiff2 (sample 1) - LPTC5, Ex...n Precursor: 169.0 Da, +1, CE: -35.0-from Analytics
Intensity, cps
2500
2000
1500
1000
500
0
125.0229
79.0184
124.0161
51.0237
69.0344
67.0188
53.0029
97.0295
107.0138
81.0342
169.0143
60
70
80
90
100
110
120
130
140
150
160
170
180
Mass/Charge, Da

Pyrogallol

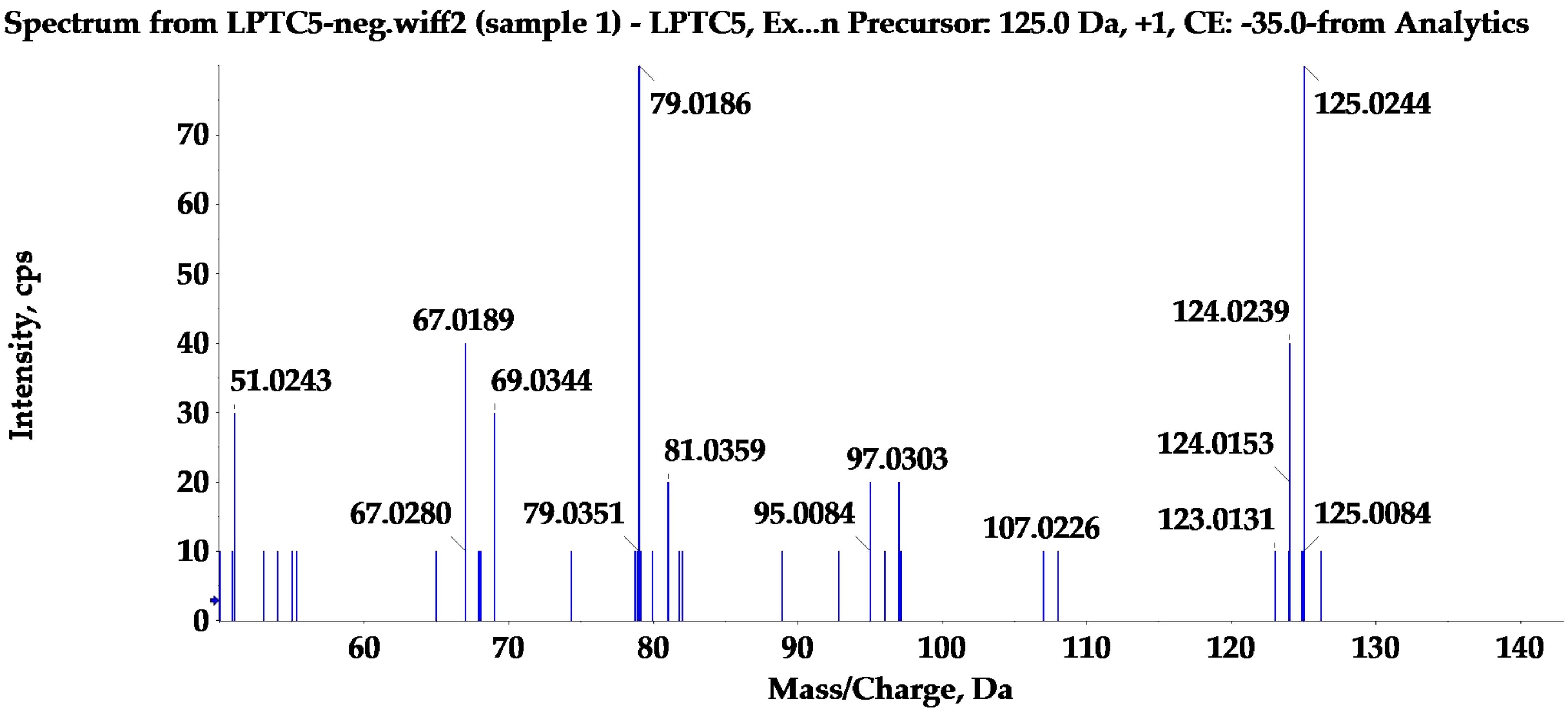

Spectrum from LPTC5-neg.wiff2 (sample 1) - LPTC5, Ex...n Precursor: 125.0 Da, +1, CE: -35.0-from Analytics
Intensity, cps
Mass/Charge, Da
79.0186
125.0244
67.0189
124.0239
51.0243
69.0344
81.0359
97.0303
124.0153
67.0280
79.0351
95.0084
107.0226
123.0131
125.0084
0
10
20
30
40
50
60
70
60
70
80
90
100
110
120
130
140

Theogallin

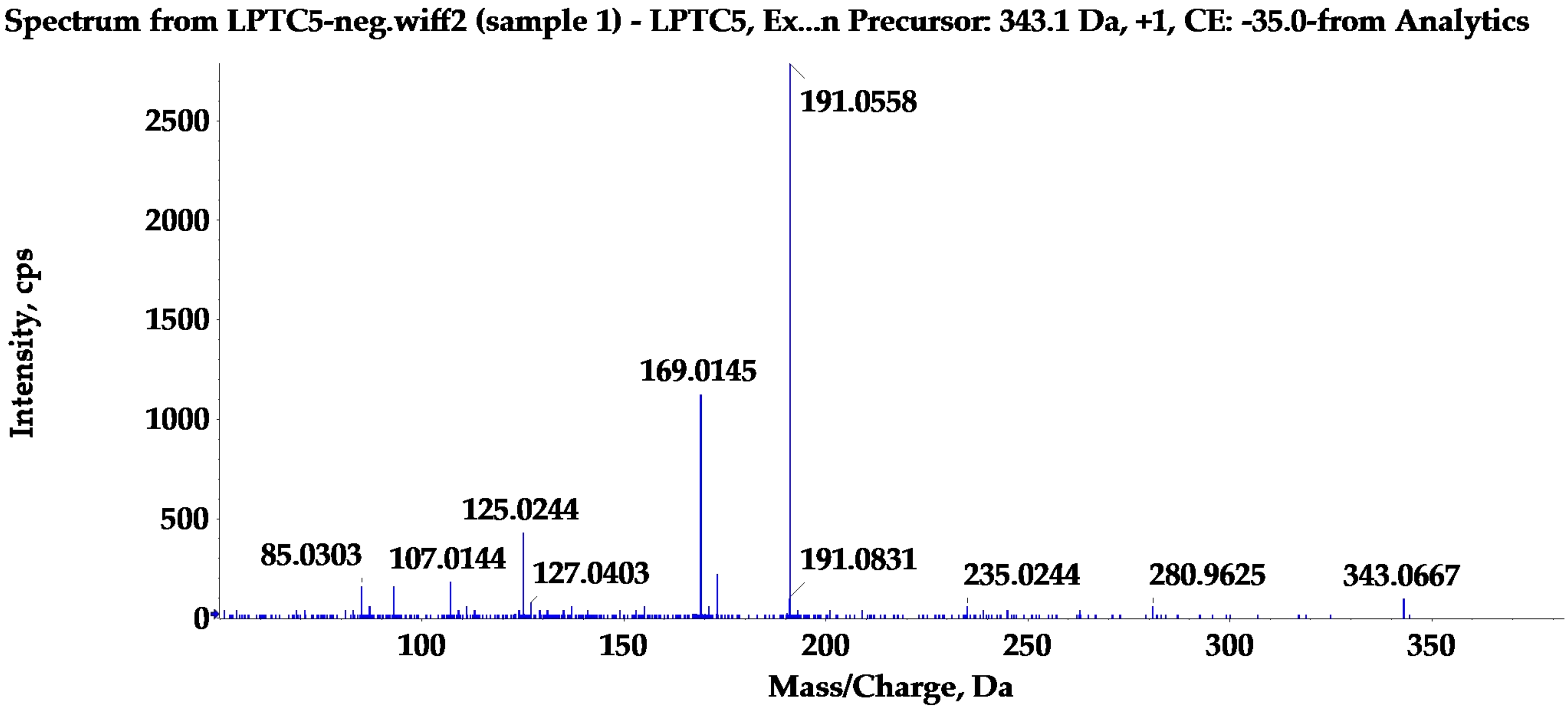

Spectrum from LPTC5-neg.wiff2 (sample 1) - LPTC5, Ex...n Precursor: 343.1 Da, +1, CE: -35.0-from Analytics
Intensity, cps
2500
2000
1500
1000
500
0
191.0558
169.0145
125.0244
85.0303
107.0144
127.0403
191.0831
235.0244
280.9625
343.0667
100
150
200
250
300
350
Mass/Charge, Da

7-Methylxanthine

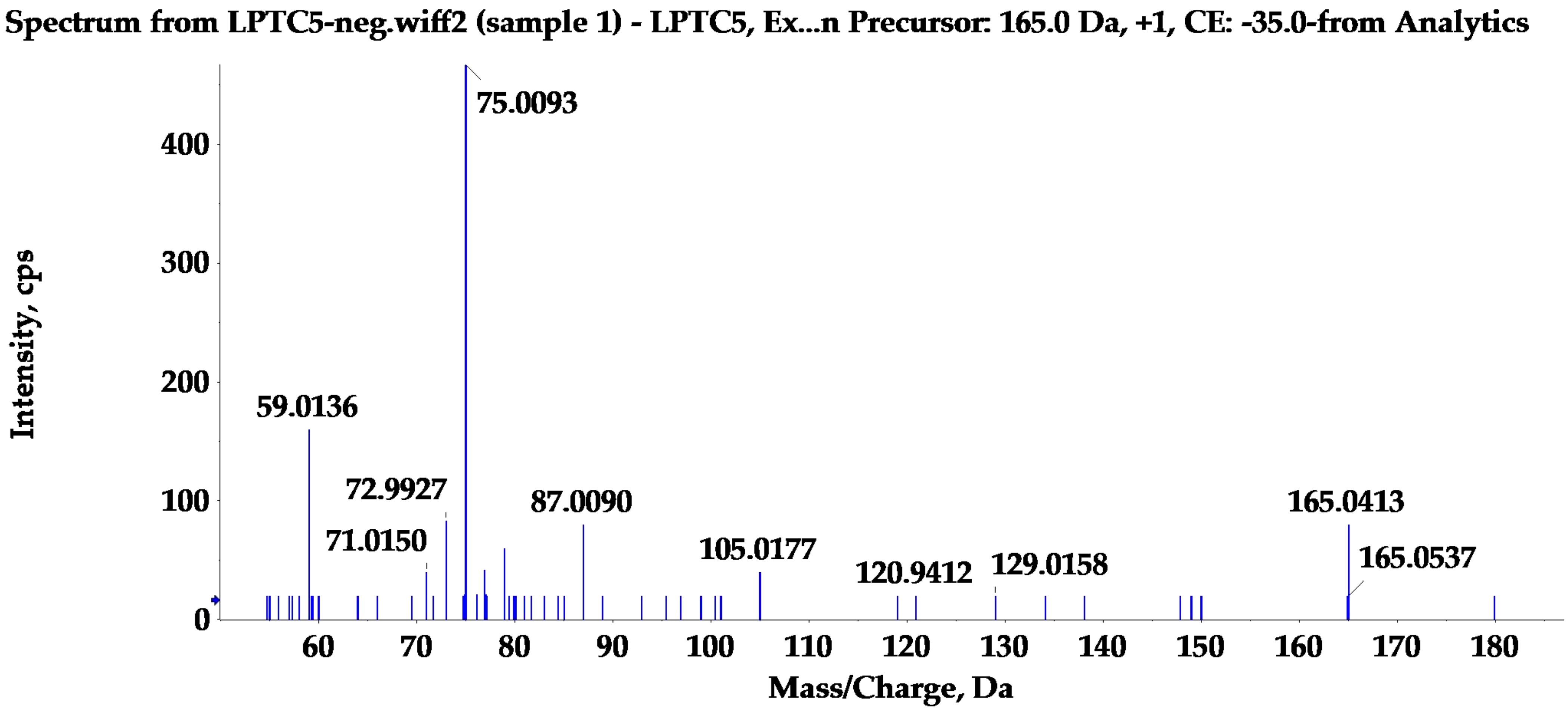

(-)Epigallocatechin

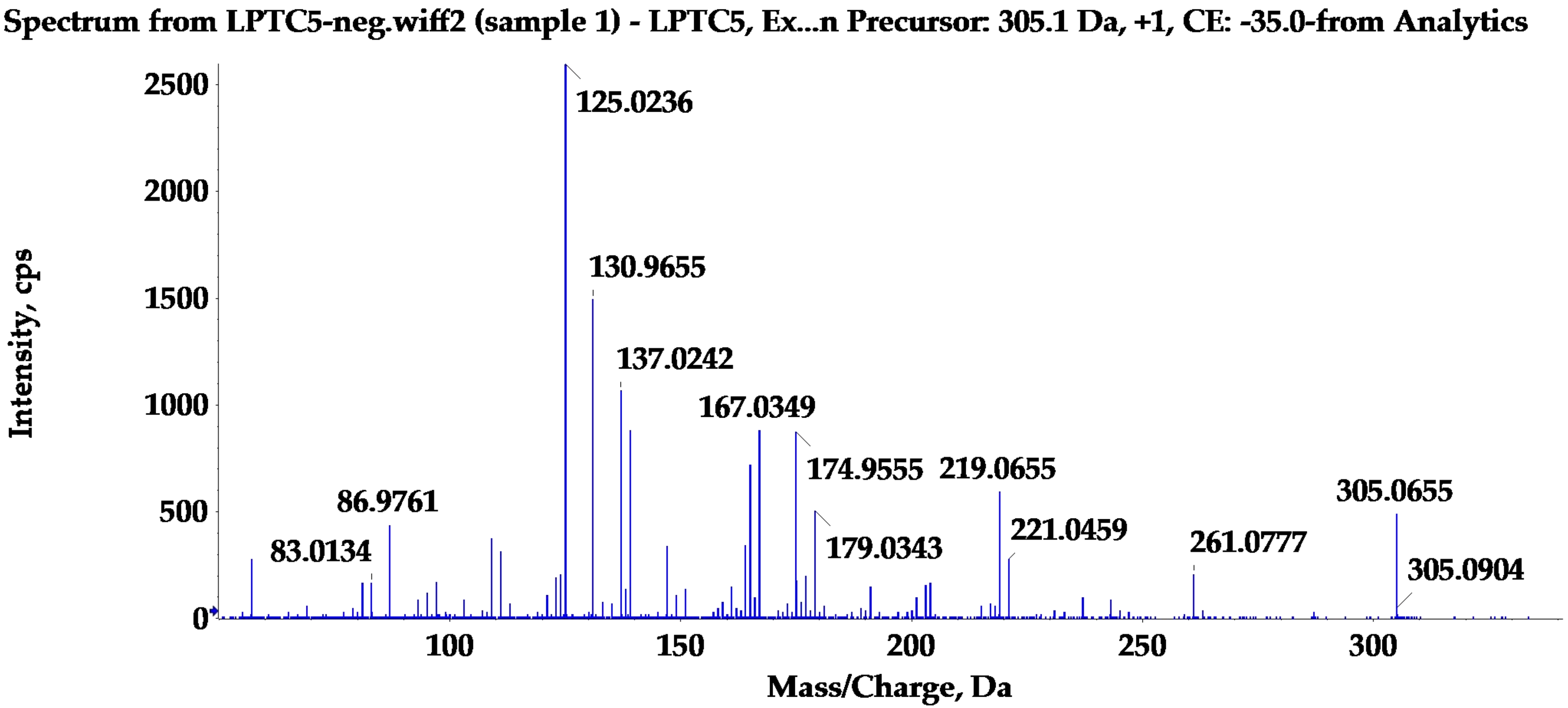

Esculin hydrate

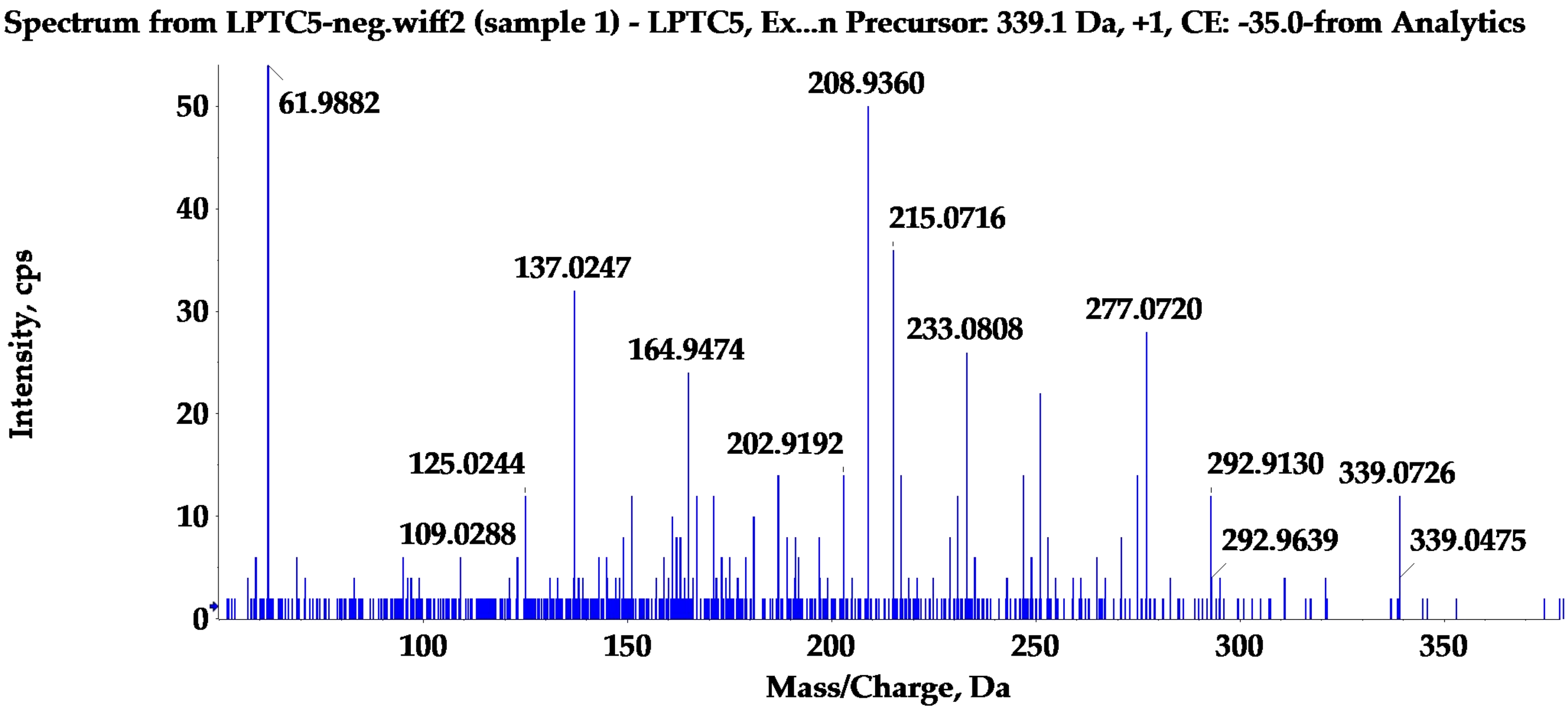

Theobromine

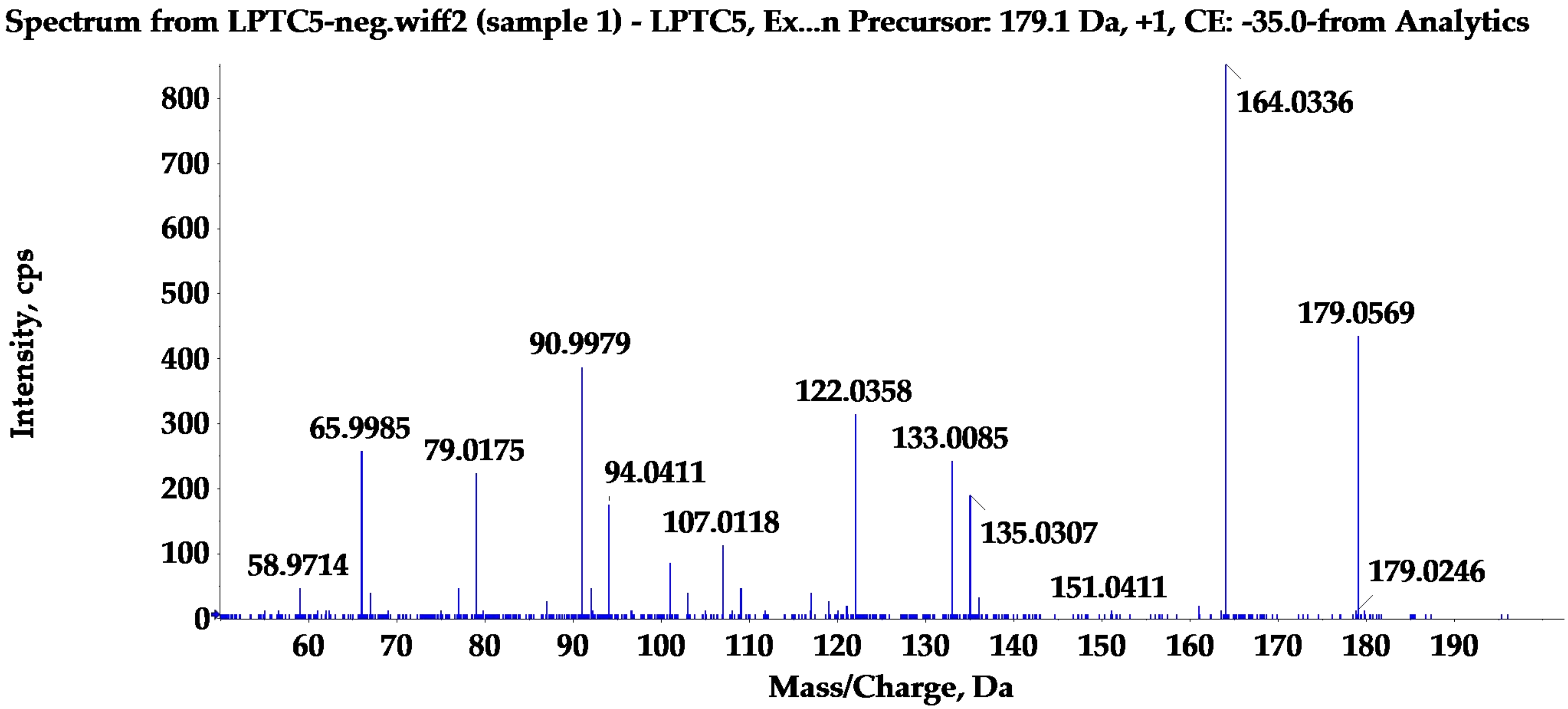

3,4-Dihydroxybenzaldehyde

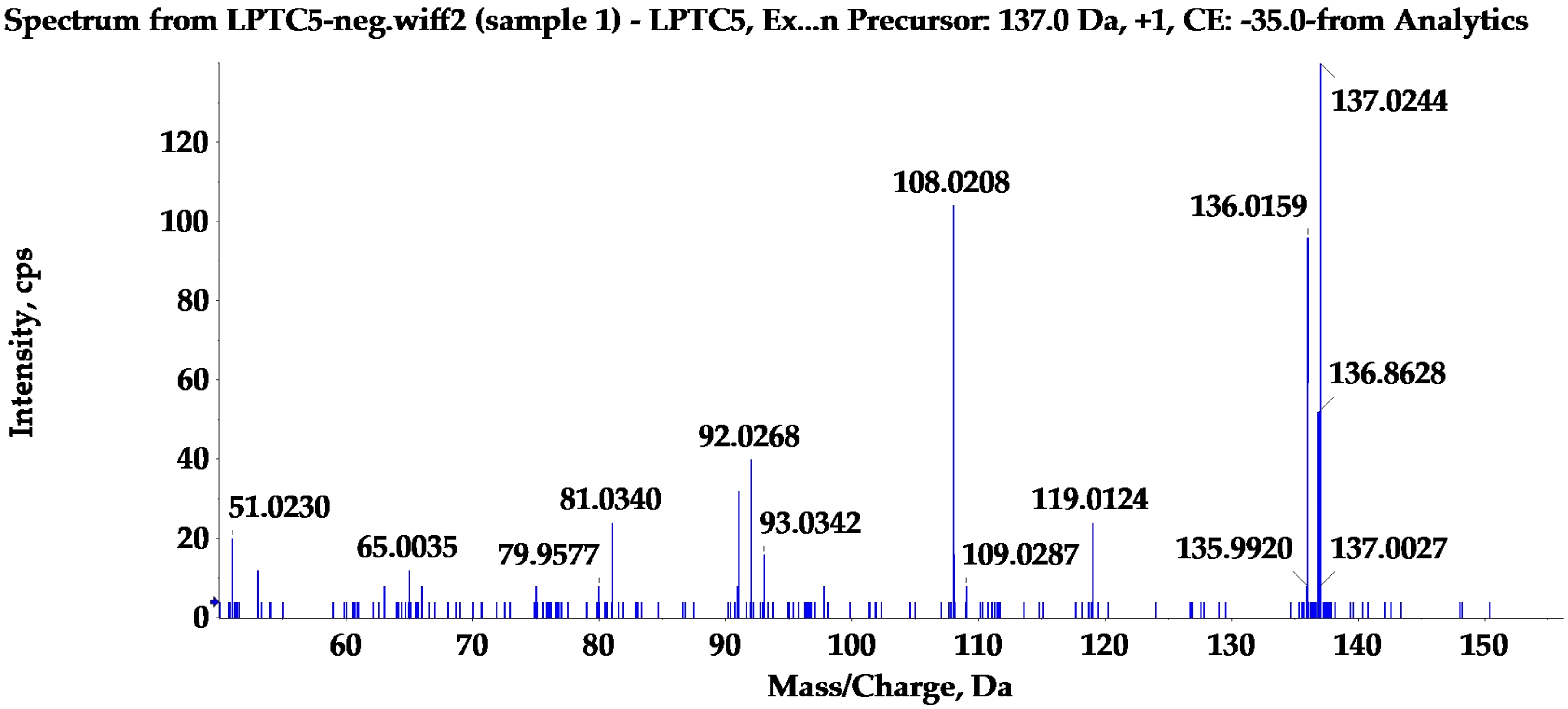

Dihydromyricetin

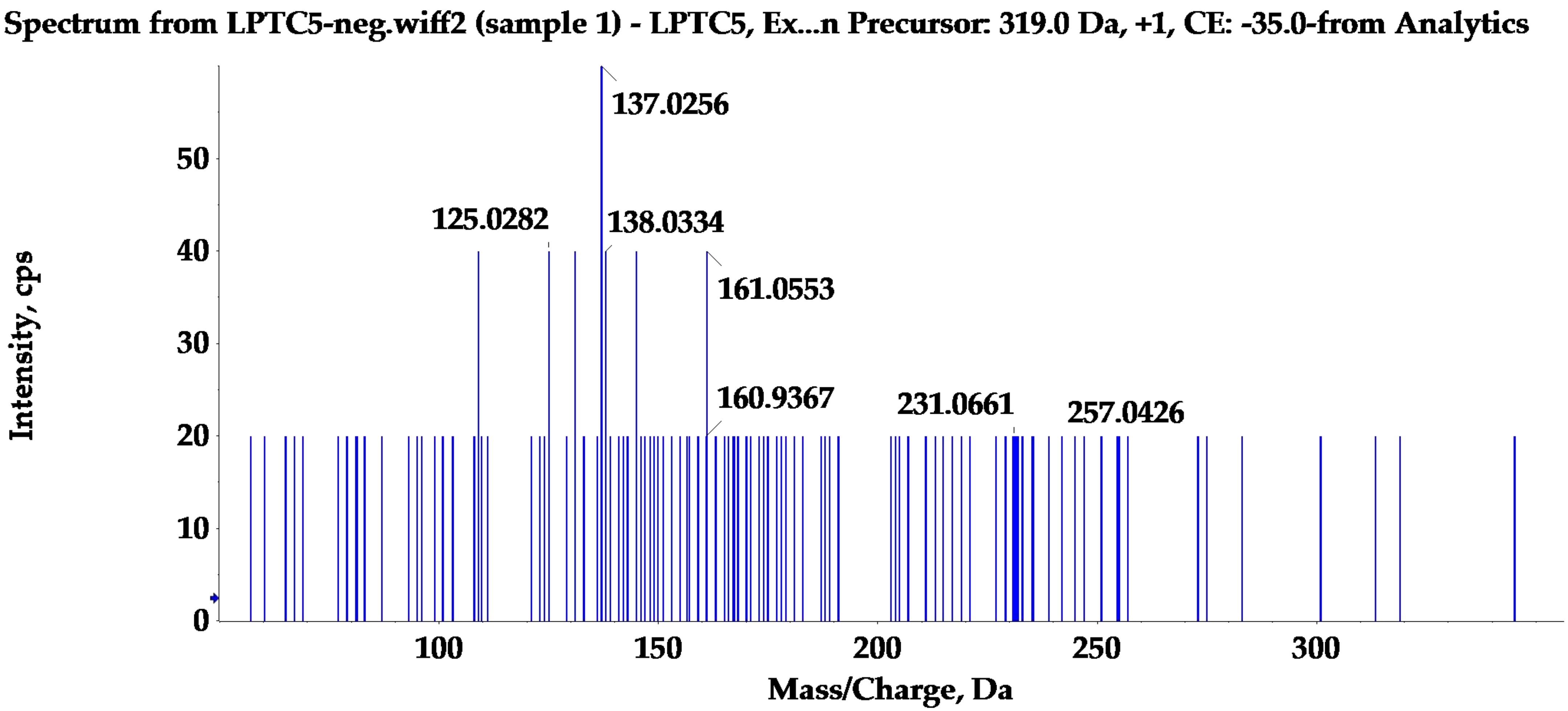

Procyanidin B2

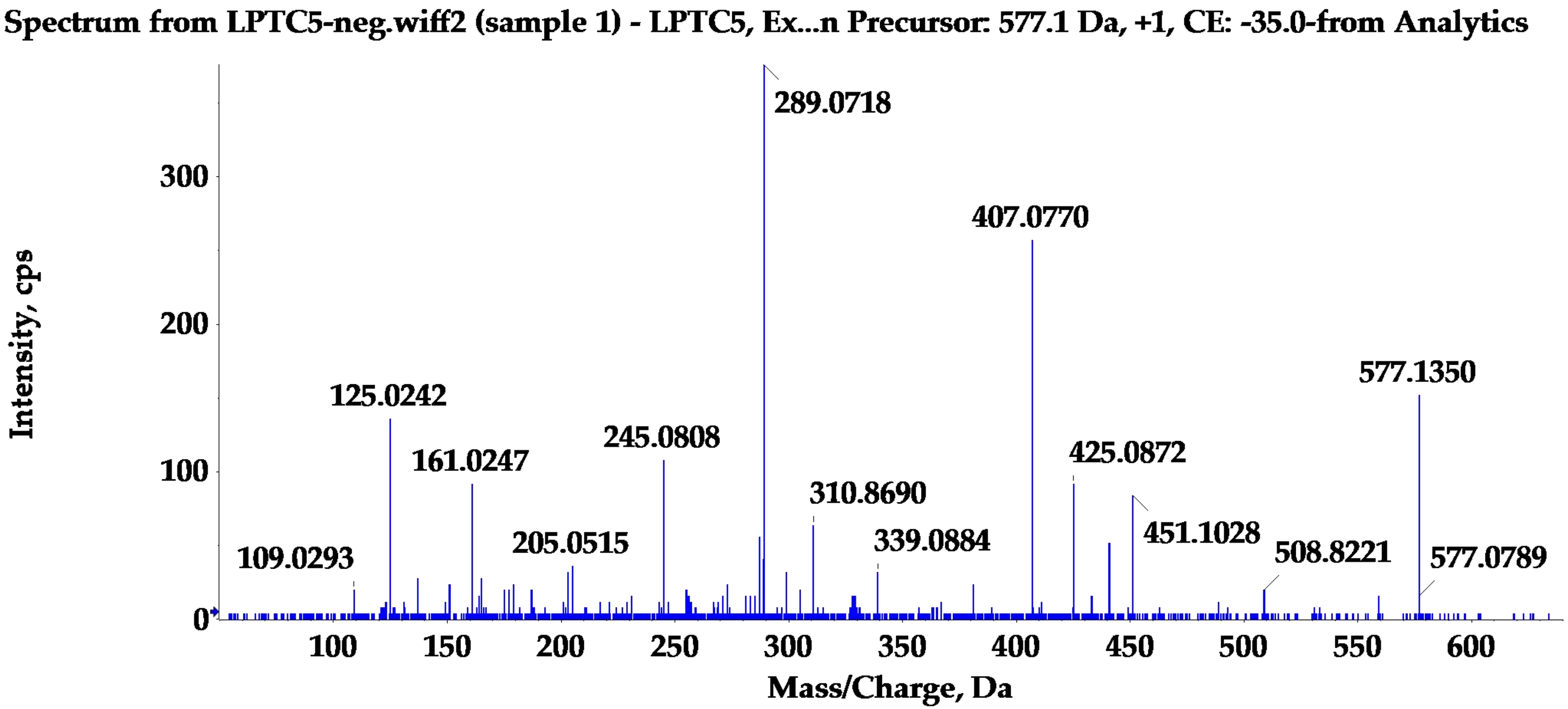

Catechin

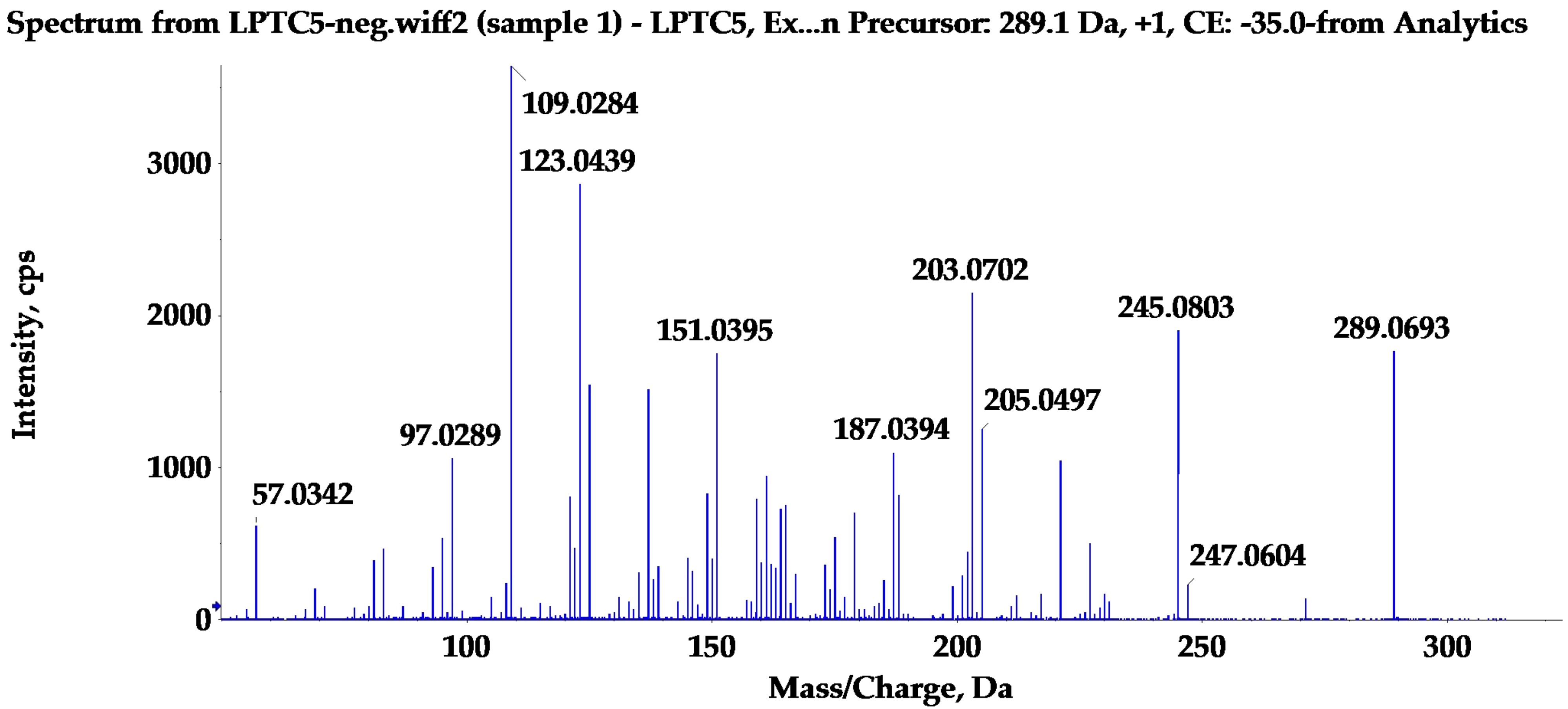
Spectrum from LPTC5-neg.wiff2 (sample 1) - LPTC5, Ex...n Precursor: 289.1 Da, +1, CE: -35.0-from Analytics
109.0284
123.0439
203.0702
245.0803
289.0693
151.0395
205.0497
187.0394
97.0289
57.0342
247.0604
3000
2000
1000
0
100
150
200
250
300
Intensity, cps
Mass/Charge, Da

Marmesin

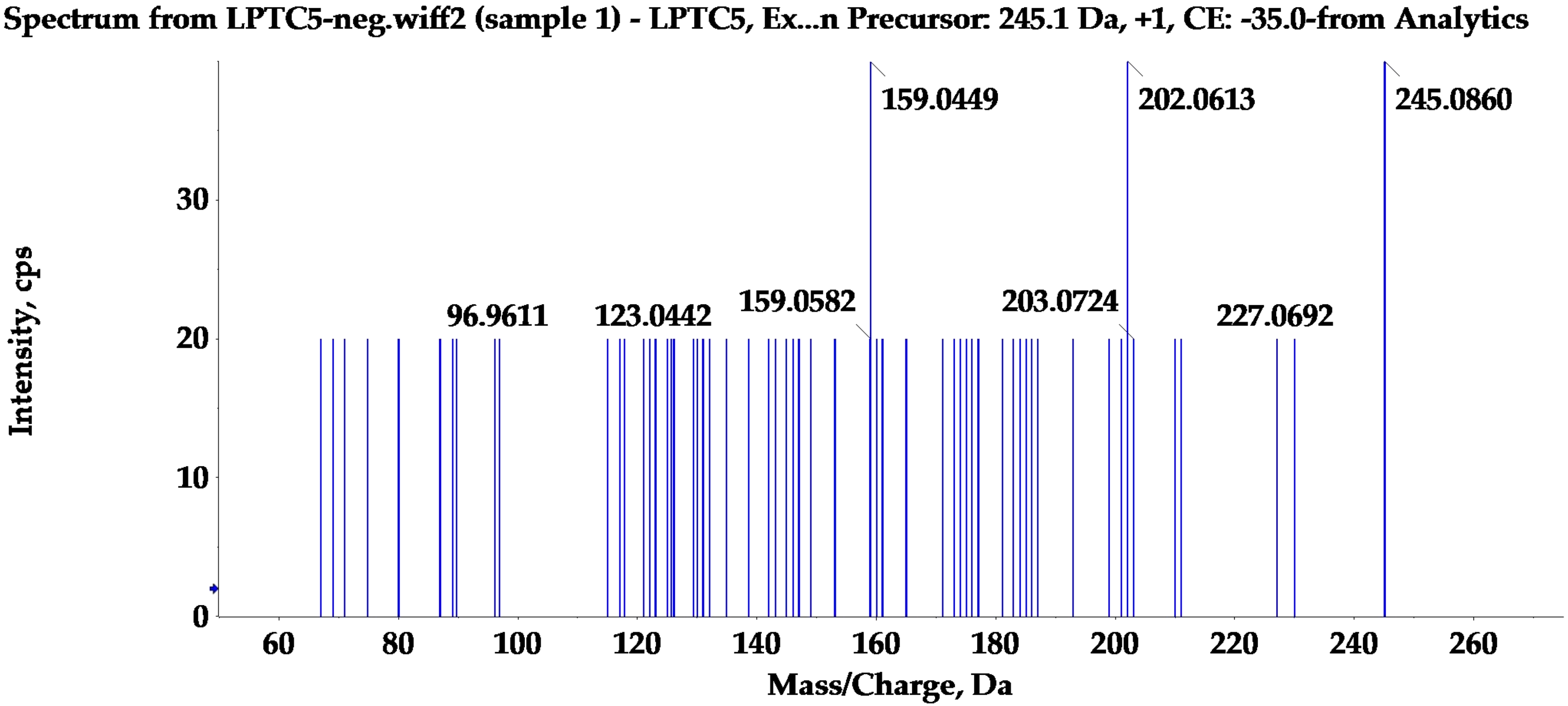

Spectrum from LPTC5-neg.wiff2 (sample 1) - LPTC5, Ex...n Precursor: 245.1 Da, +1, CE: -35.0-from Analytics
Intensity, cps
Mass/Charge, Da
159.0449
202.0613
245.0860
96.9611
123.0442
159.0582
203.0724
227.0692
0
10
20
30
60
80
100
120
140
160
180
200
220
240
260

Dihydrokaempferol

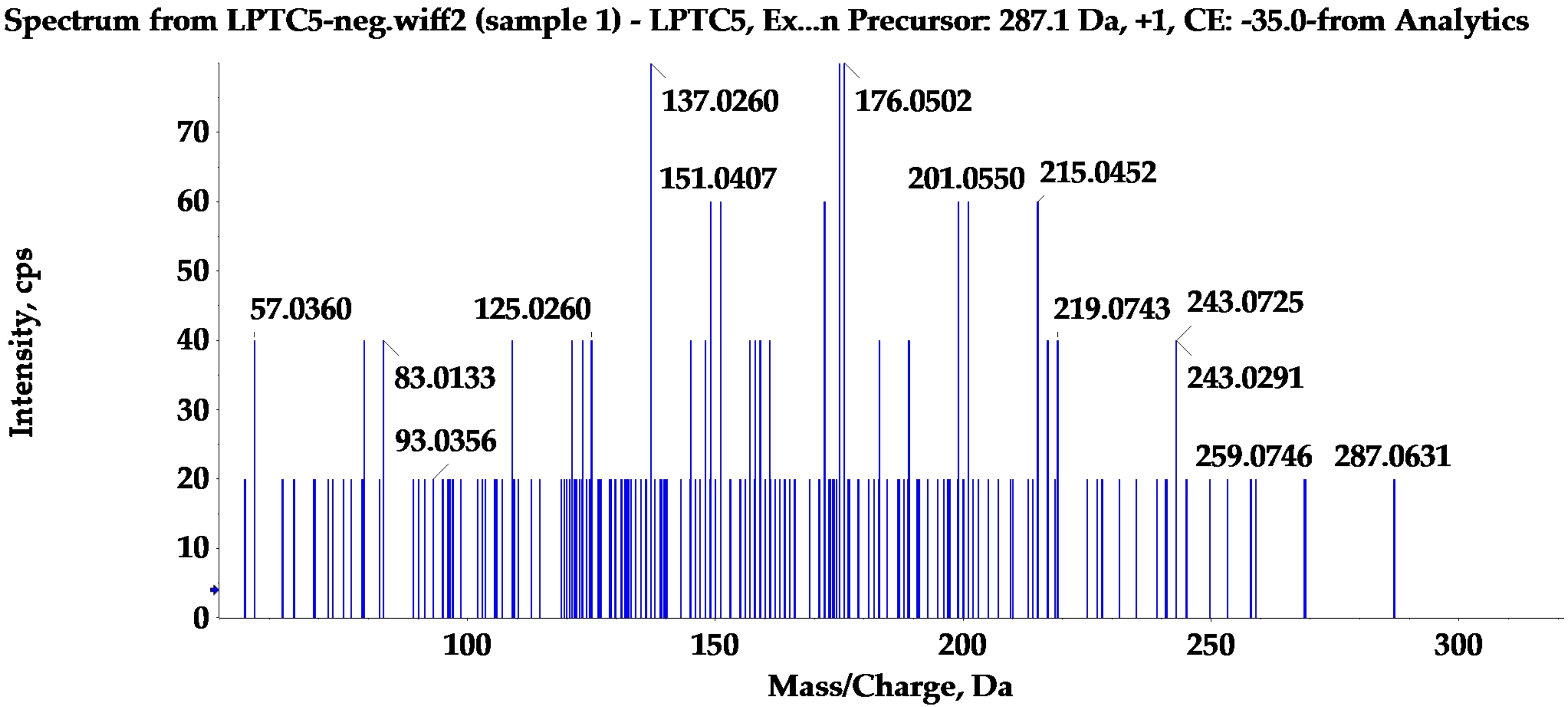

Methyl gallate

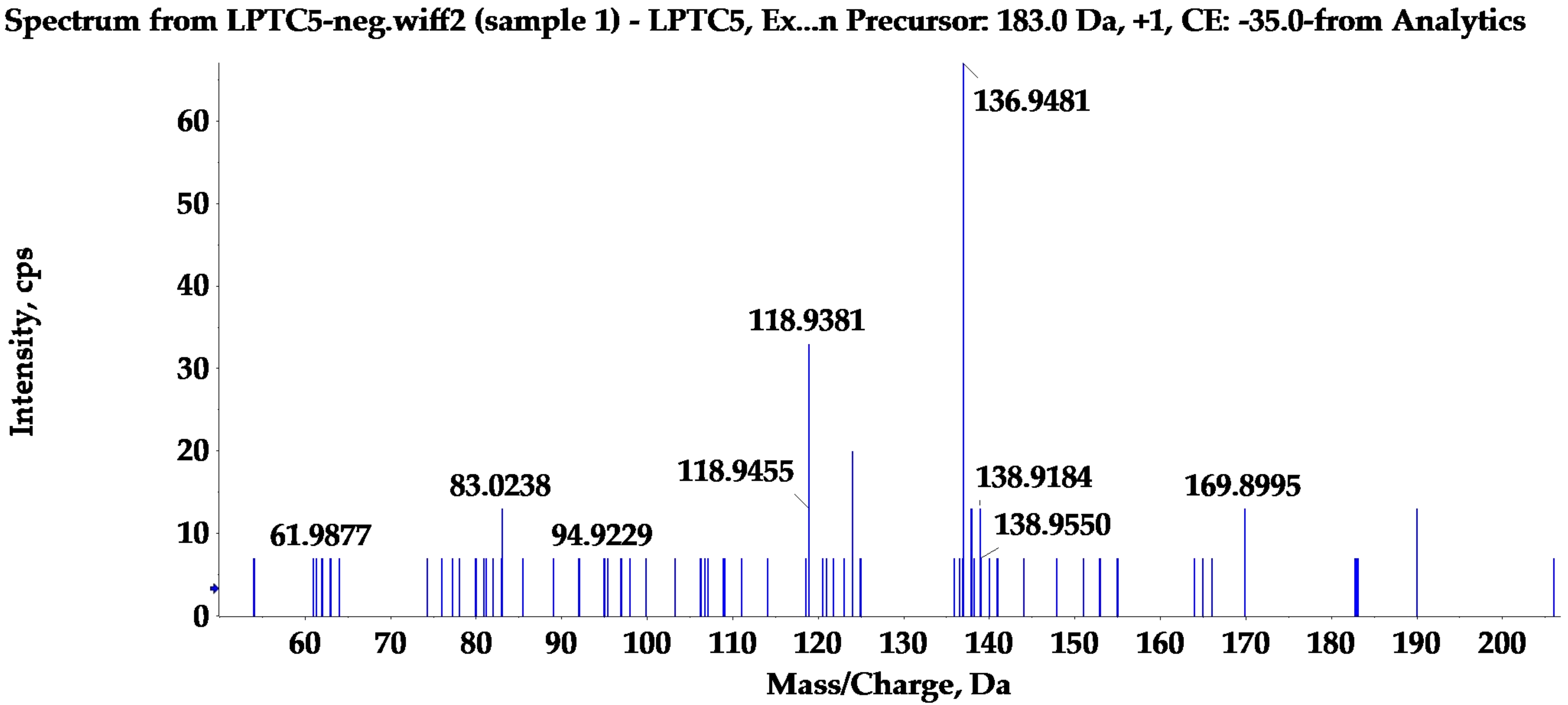

Ethyl ferulate

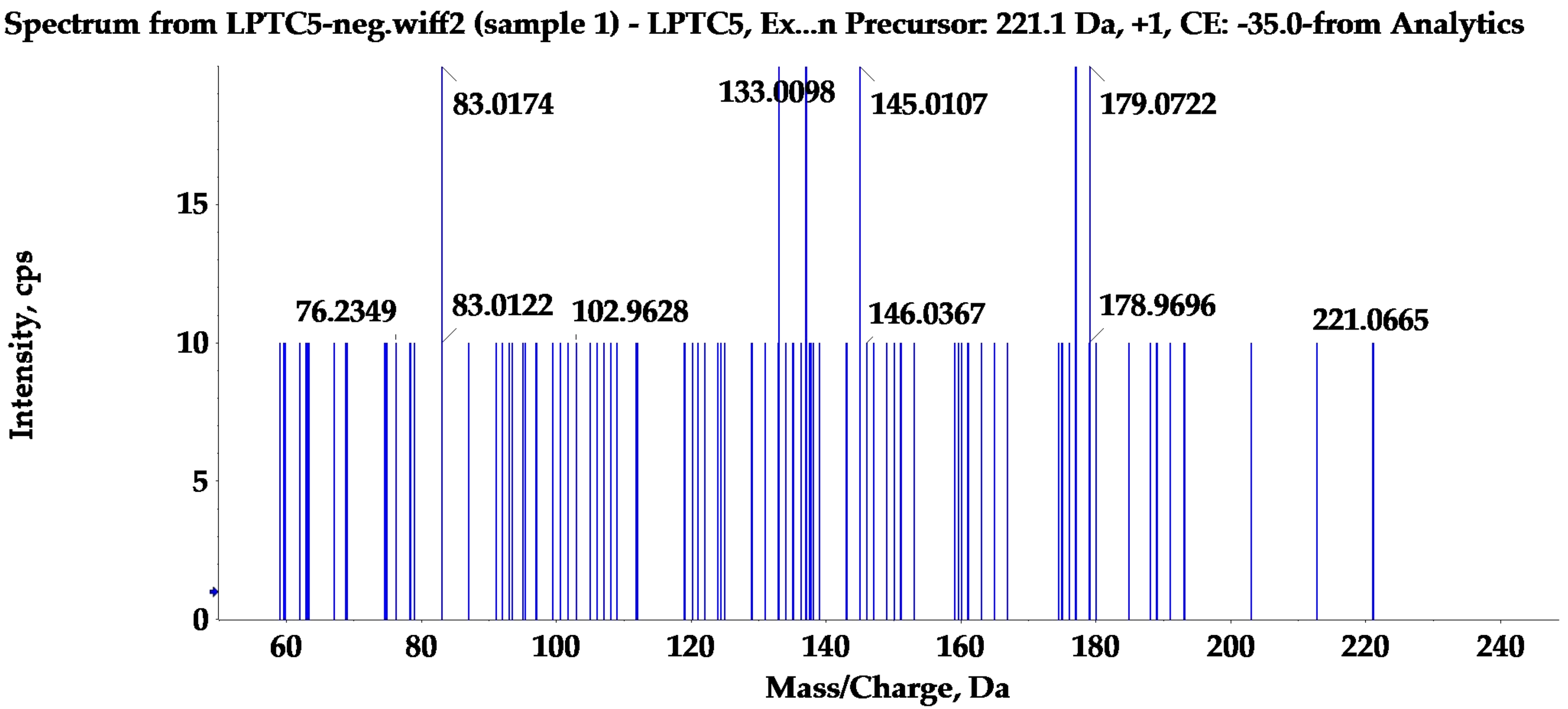

3-pcoumaroylquinic acid

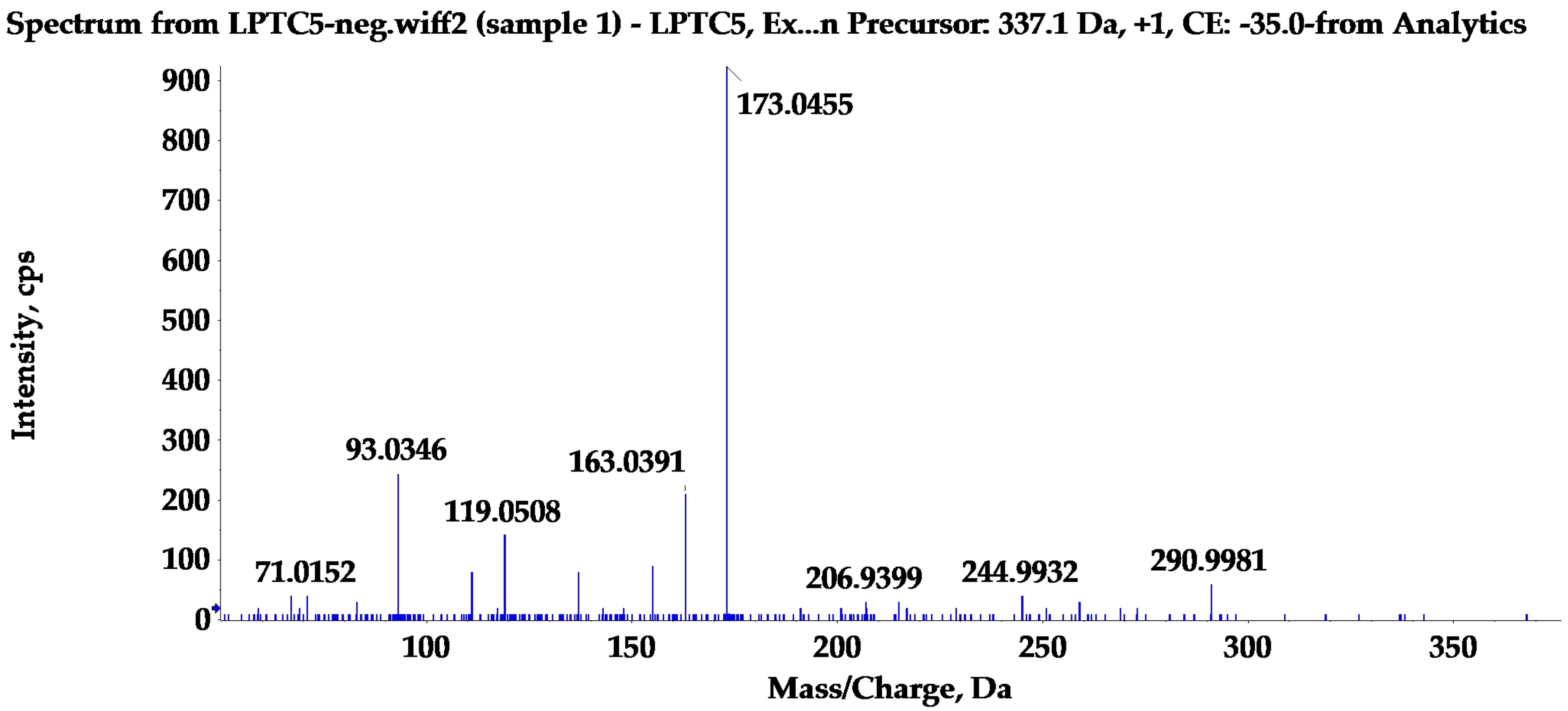

Shikimic Acid

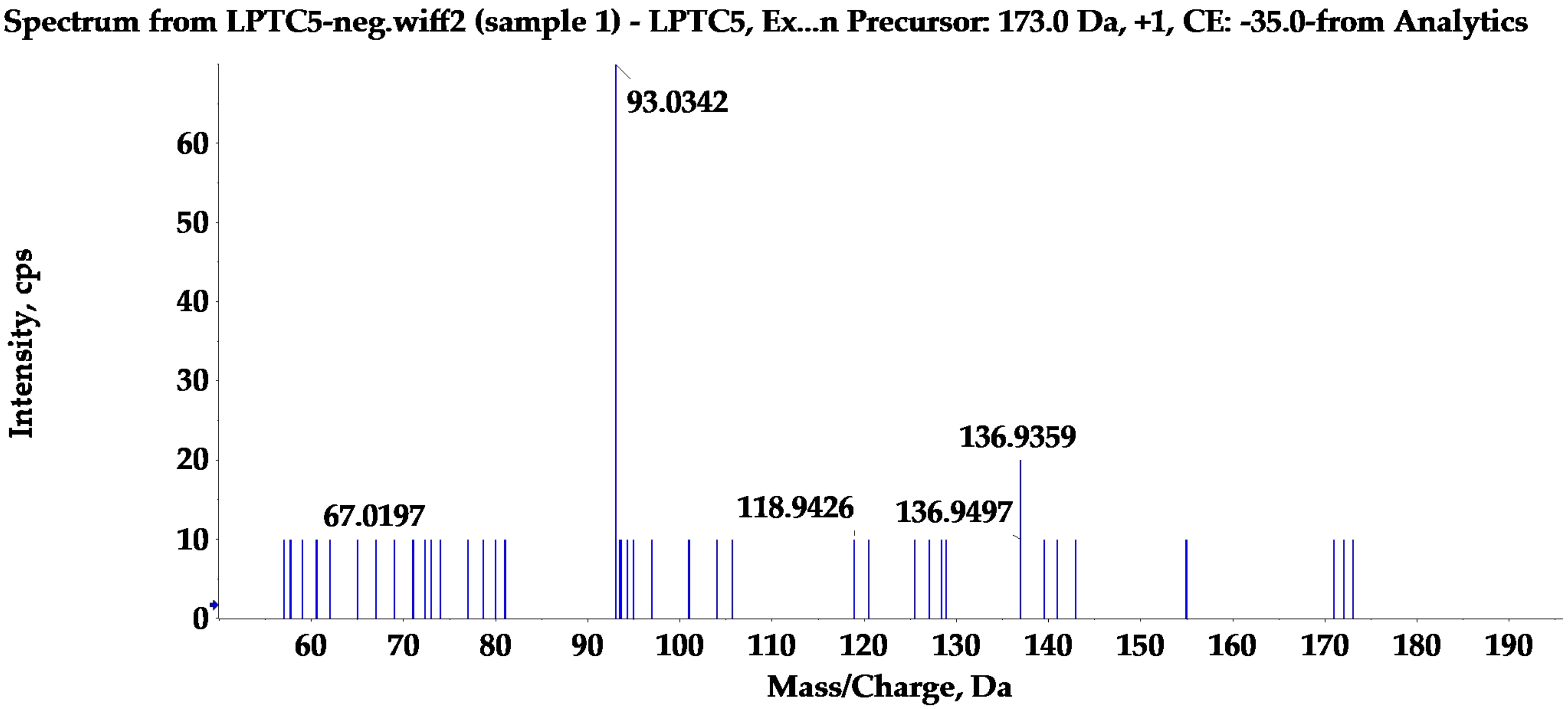

Esculetin

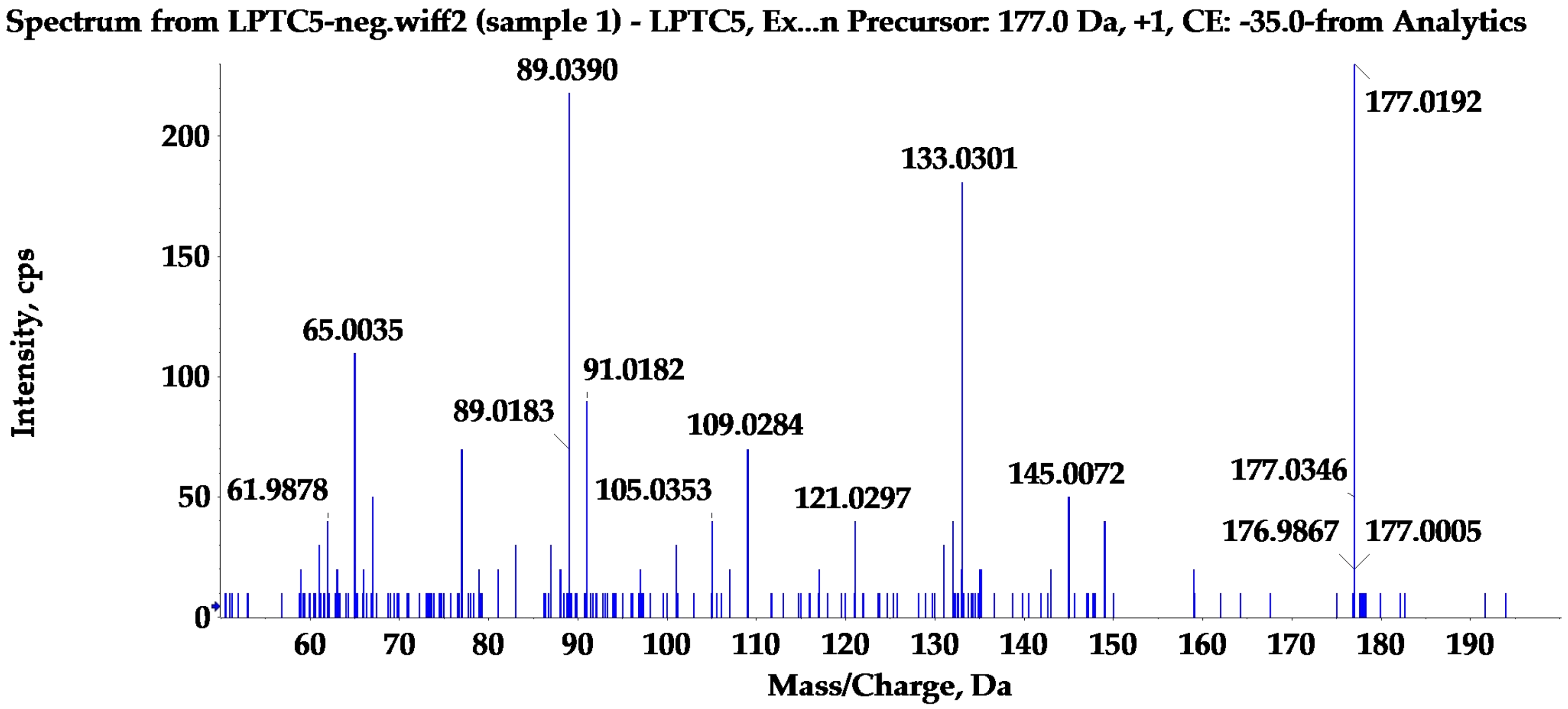

Spectrum from LPTC5-neg.wiff2 (sample 1) - LPTC5, Ex...n Precursor: 177.0 Da, +1, CE: -35.0-from Analytics
Intensity, cps
Mass/Charge, Da
89.0390
177.0192
133.0301
65.0035
91.0182
89.0183
109.0284
61.9878
105.0353
121.0297
145.0072
177.0346
176.9867
177.0005
0
50
100
150
200
60
70
80
90
100
110
120
130
140
150
160
170
180
190

Epigallocatechin 3-O-gallate（EGCG)

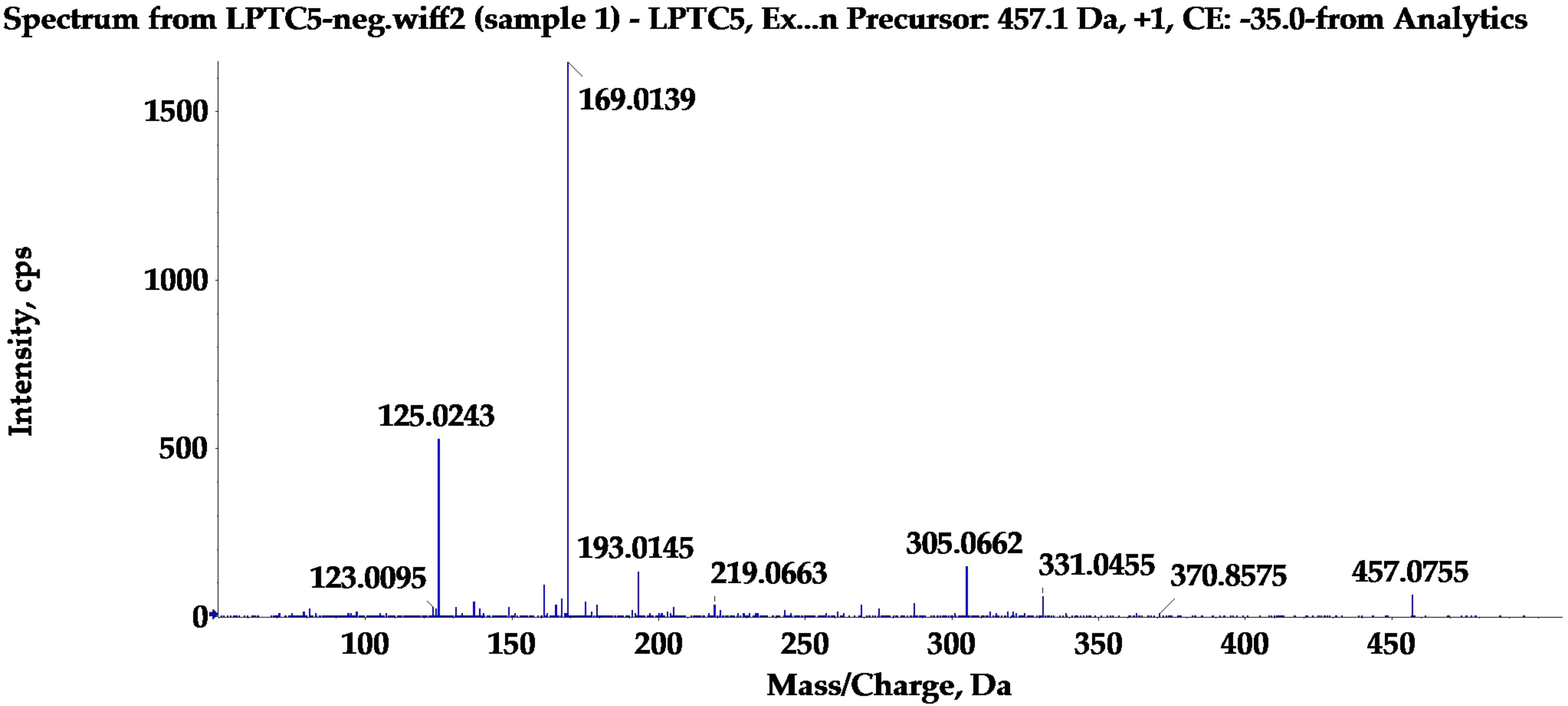

Schaftoside

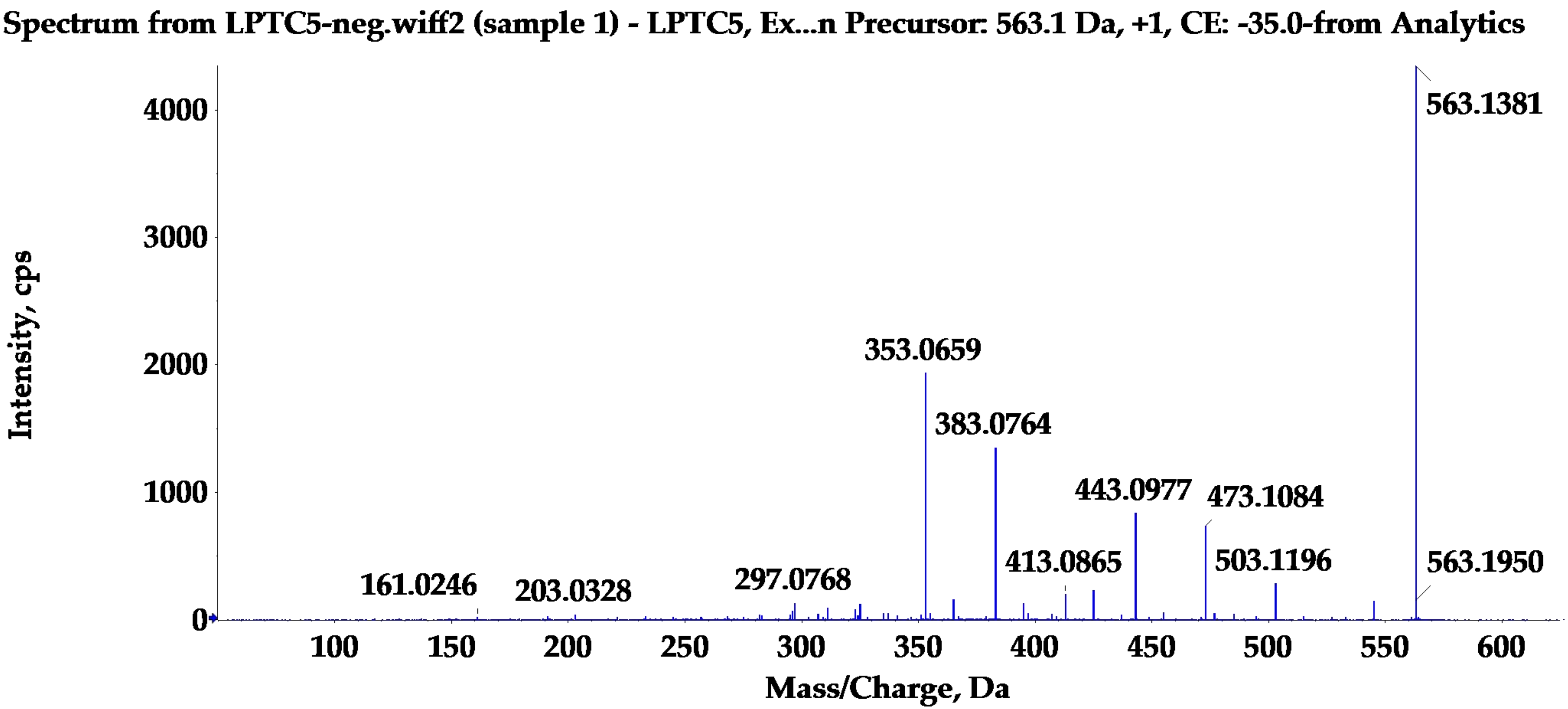

Quercetin 3-O-rhamnoside-7-O-glucoside

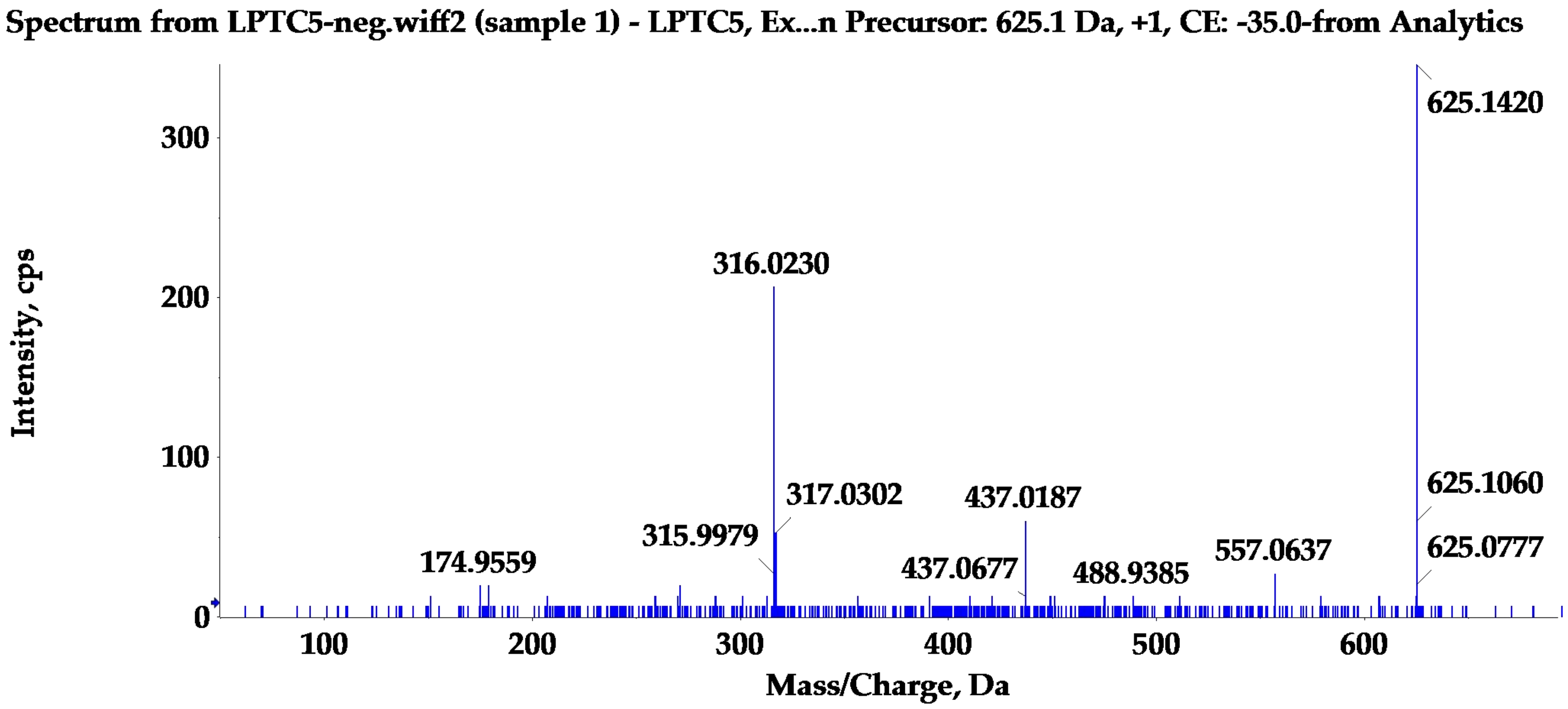

Myricetin 3-O-beta-D-glucopyranoside

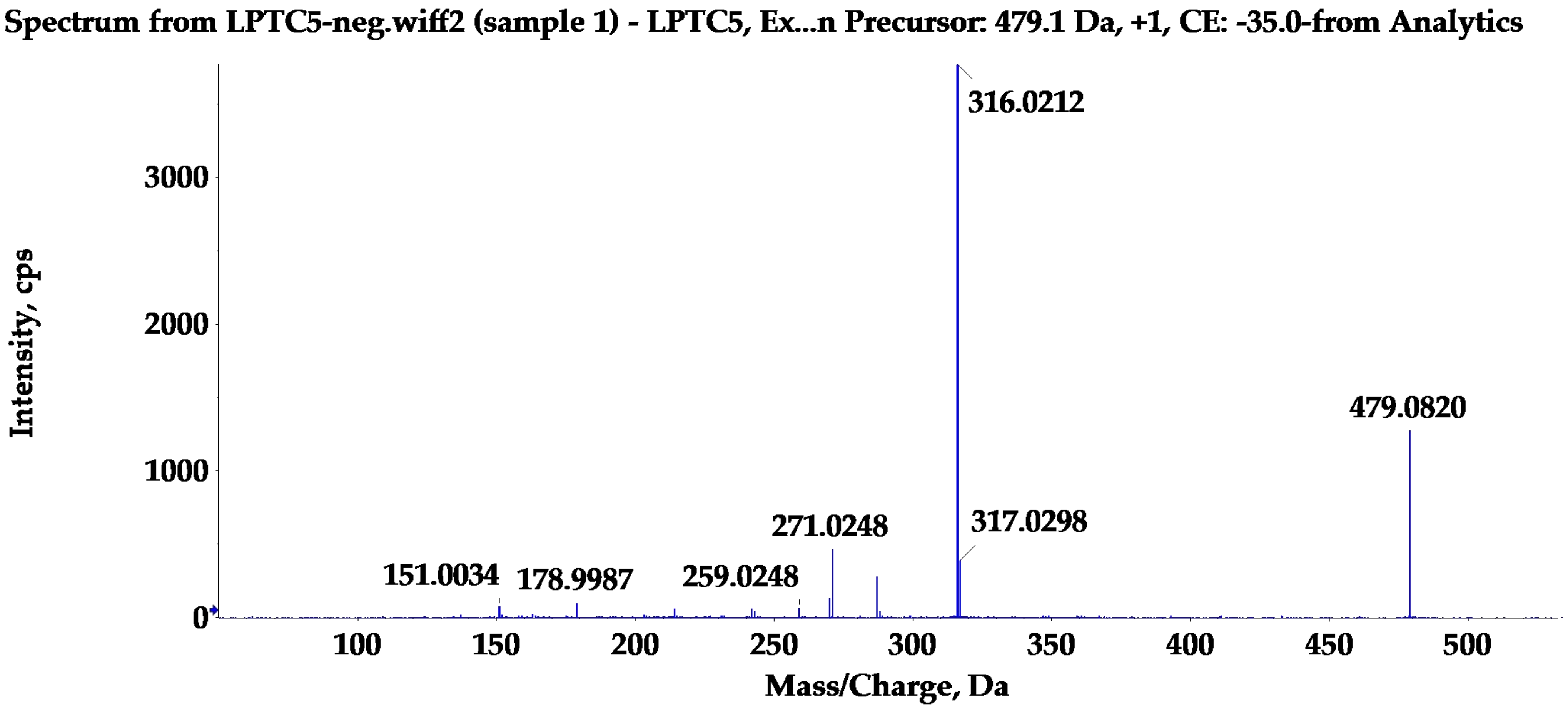

(-)-Epicatechin-3-O-gallate

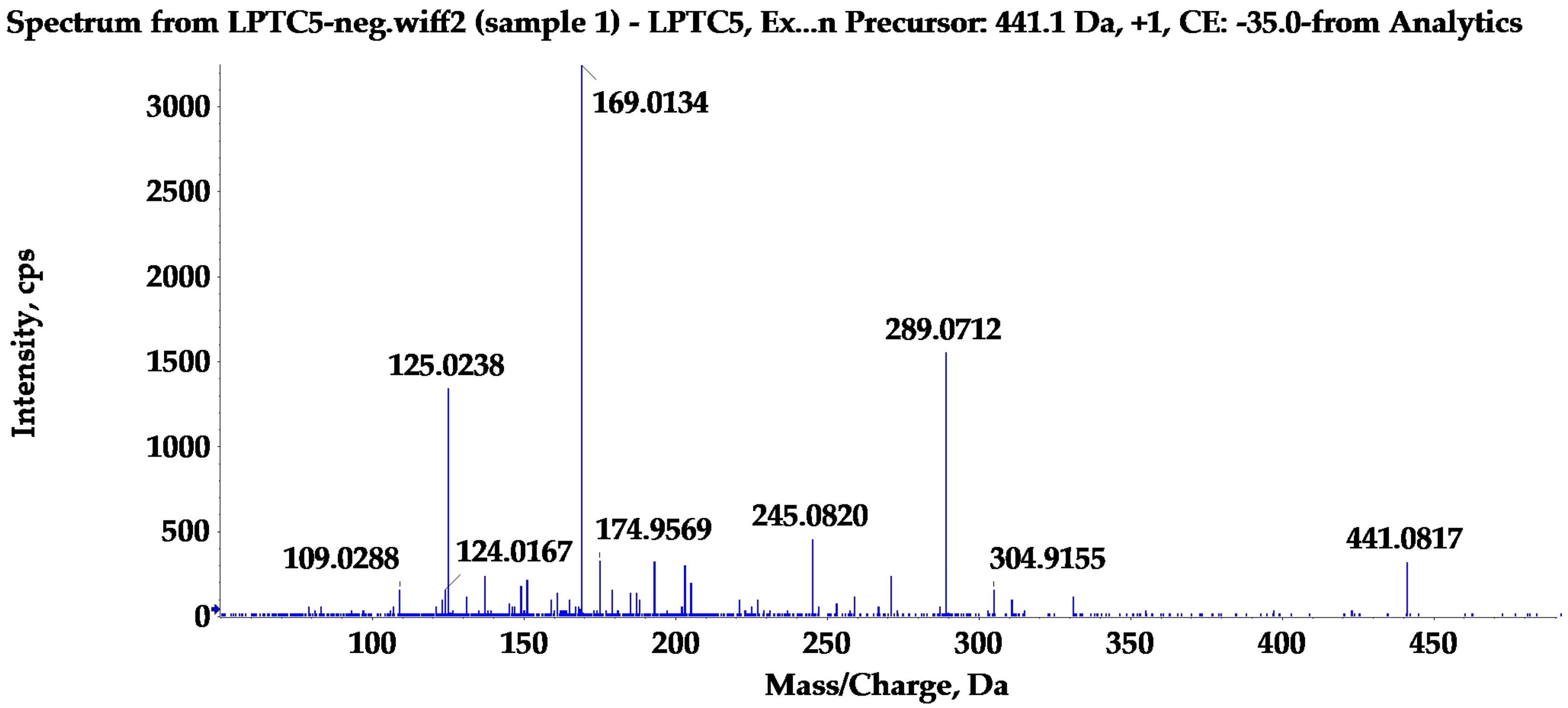

Ellagic acid

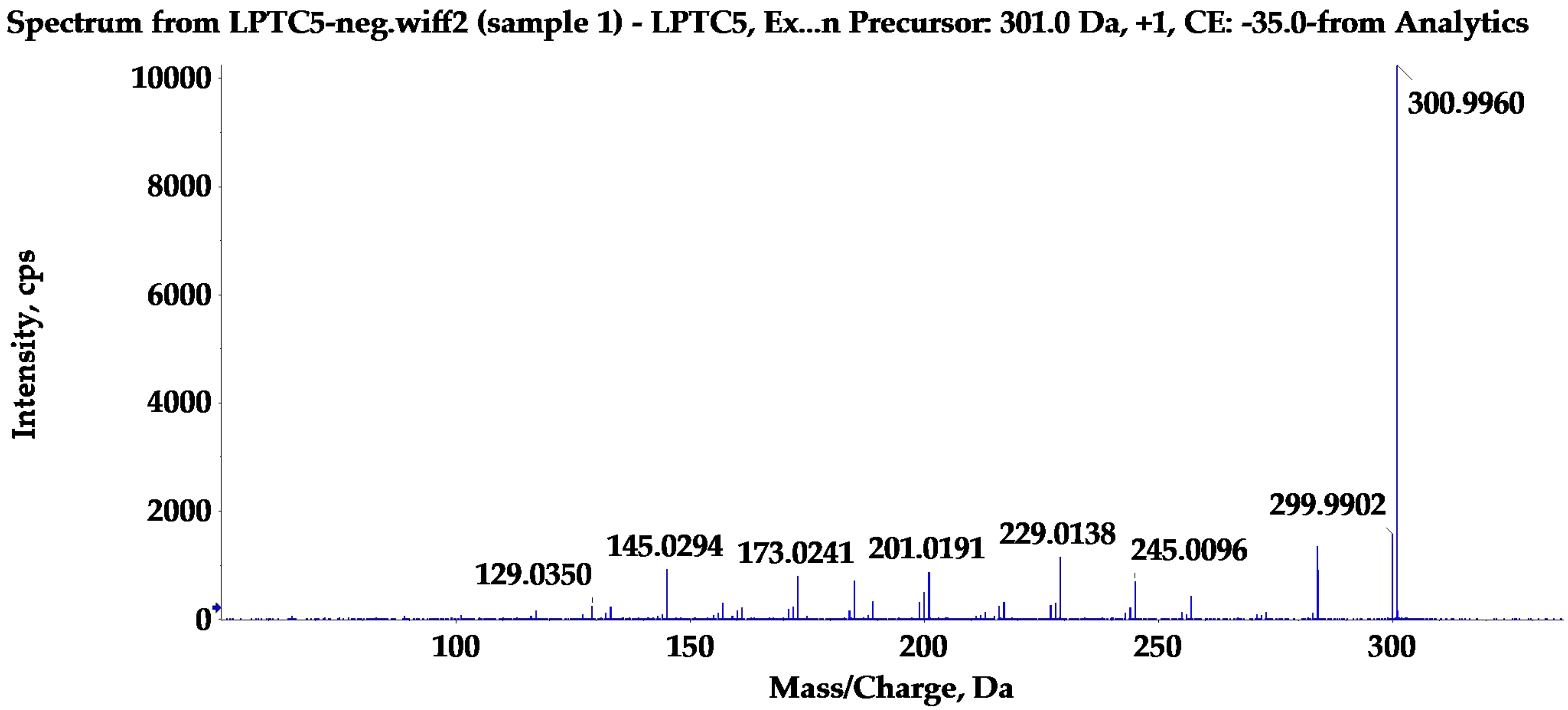

Quercetin

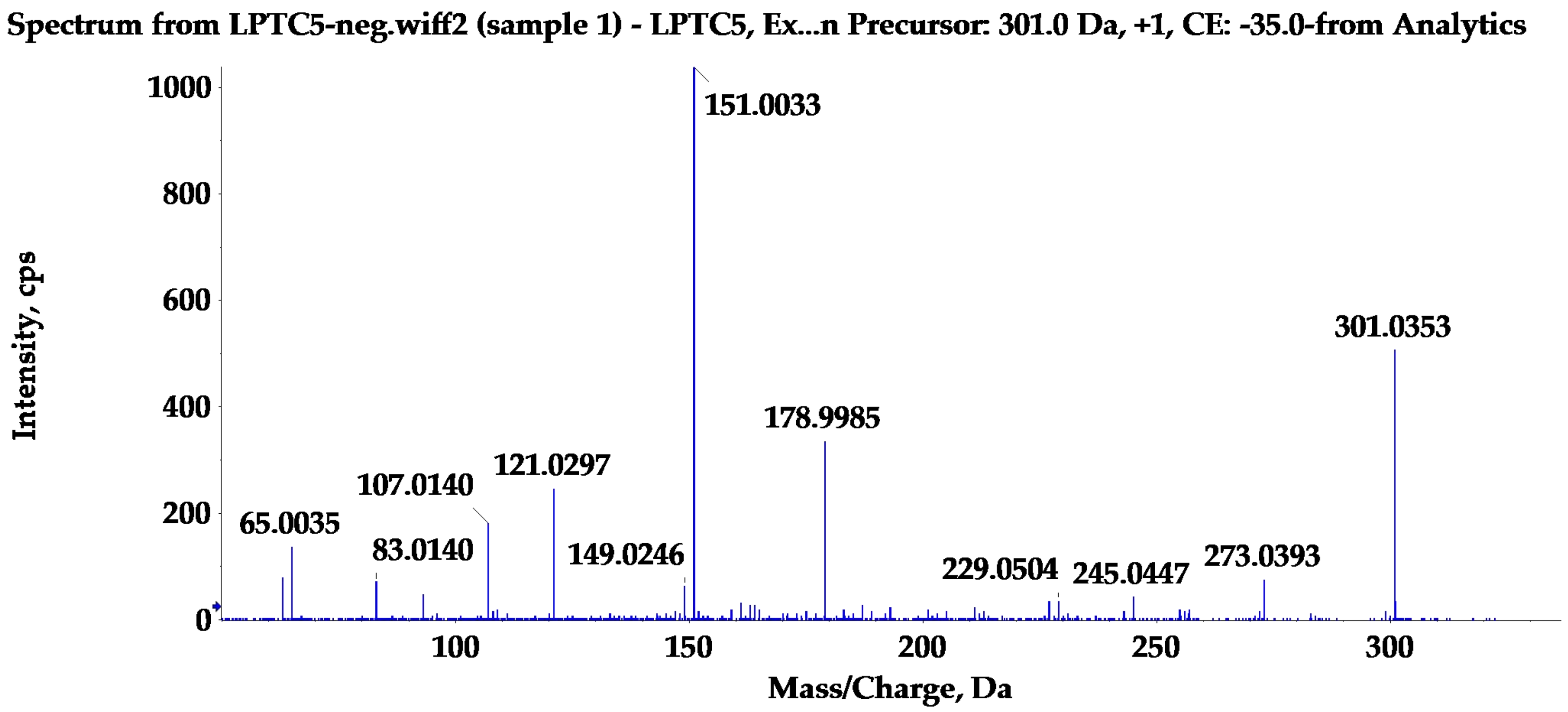
Spectrum from LPTC5-neg.wiff2 (sample 1) - LPTC5, Ex...n Precursor: 301.0 Da, +1, CE: -35.0-from Analytics
Intensity, cps
1000
800
600
400
200
0
151.0033
301.0353
178.9985
121.0297
107.0140
65.0035
83.0140
149.0246
229.0504
245.0447
273.0393
100
150
200
250
300
Mass/Charge, Da

6-O-(beta-D-Xylopyranosyl)-beta-D-glucopyranose

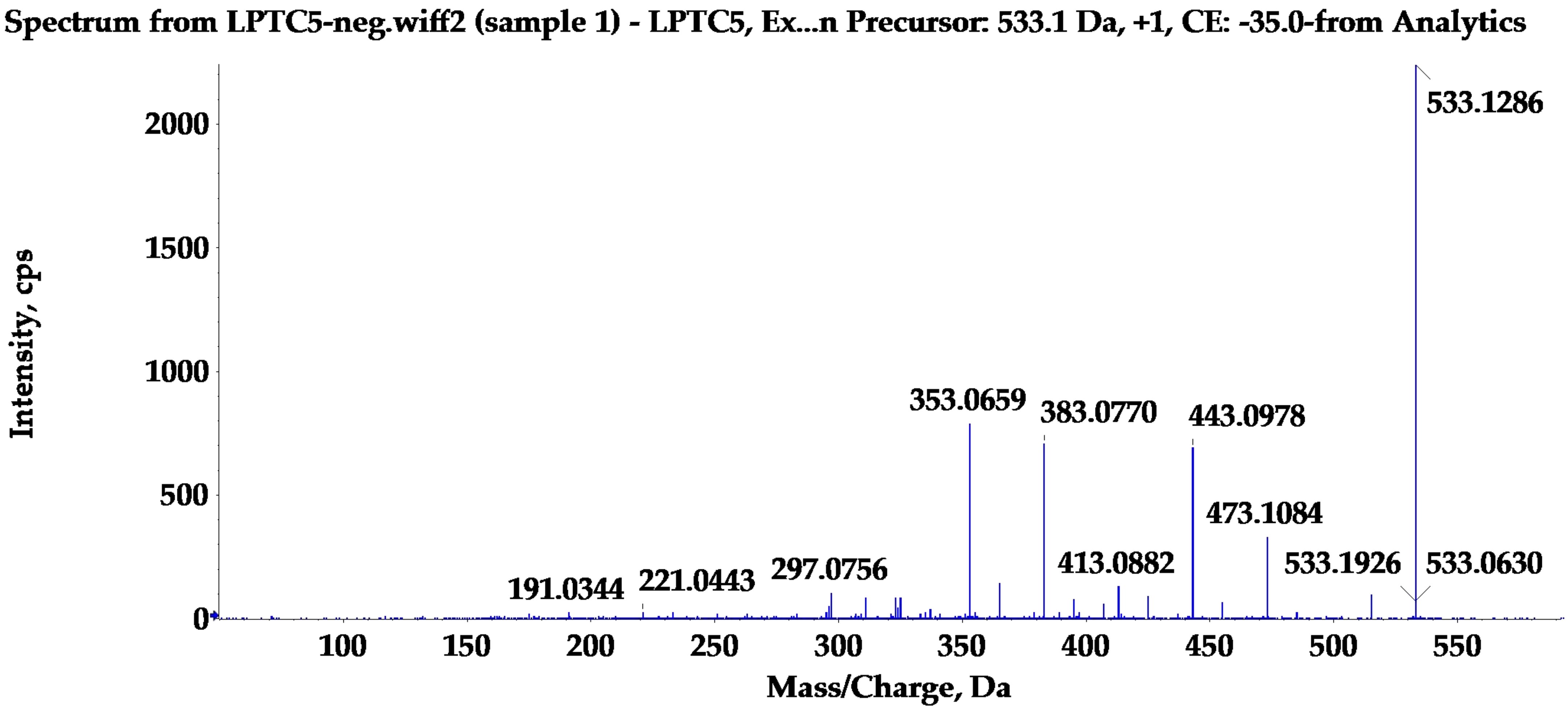

Rutin

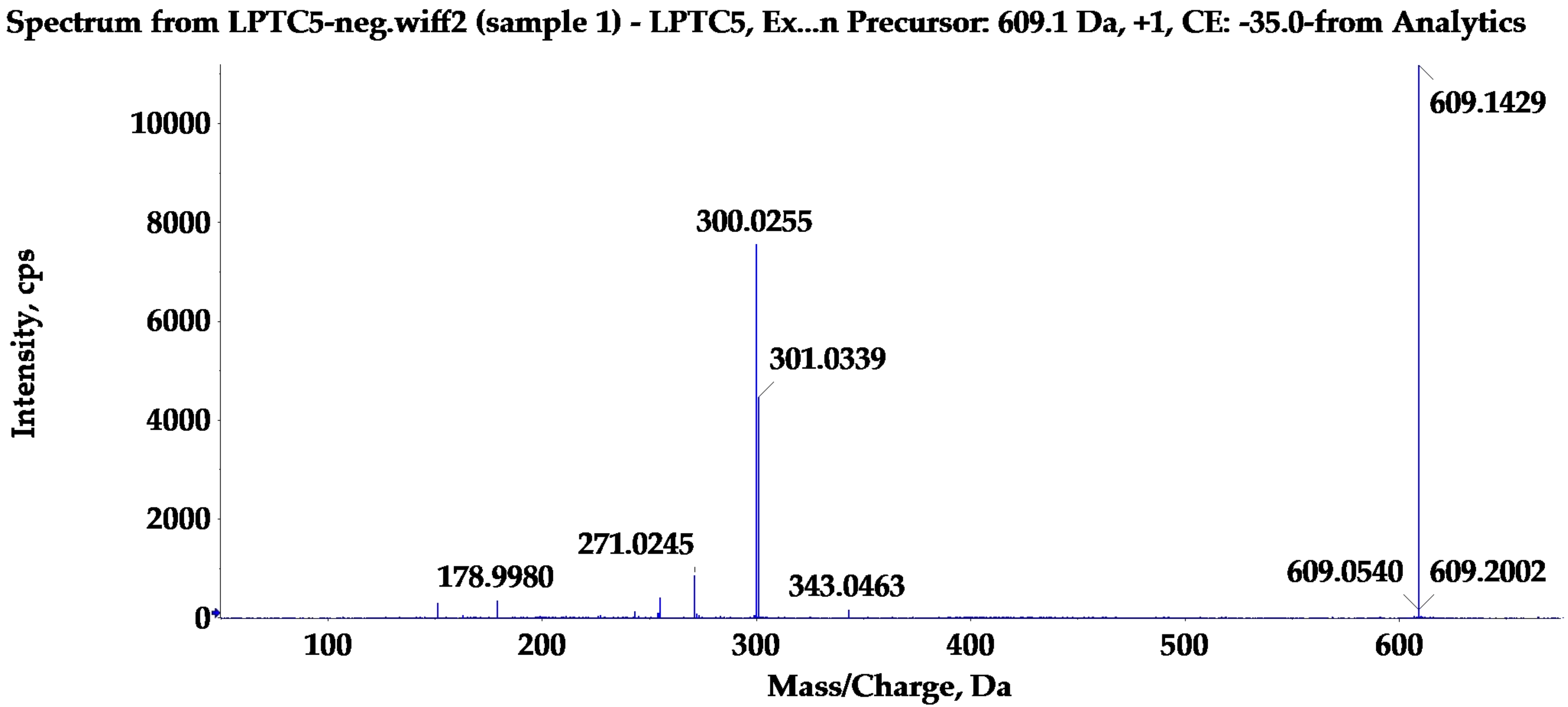
Spectrum from LPTC5-neg.wiff2 (sample 1) - LPTC5, Ex...n Precursor: 609.1 Da, +1, CE: -35.0-from Analytics
Intensity, cps
Mass/Charge, Da
609.1429
300.0255
301.0339
271.0245
178.9980
343.0463
609.0540
609.2002
10000
8000
6000
4000
2000
0
100
200
300
400
500
600

Apigenin 7-glucoside

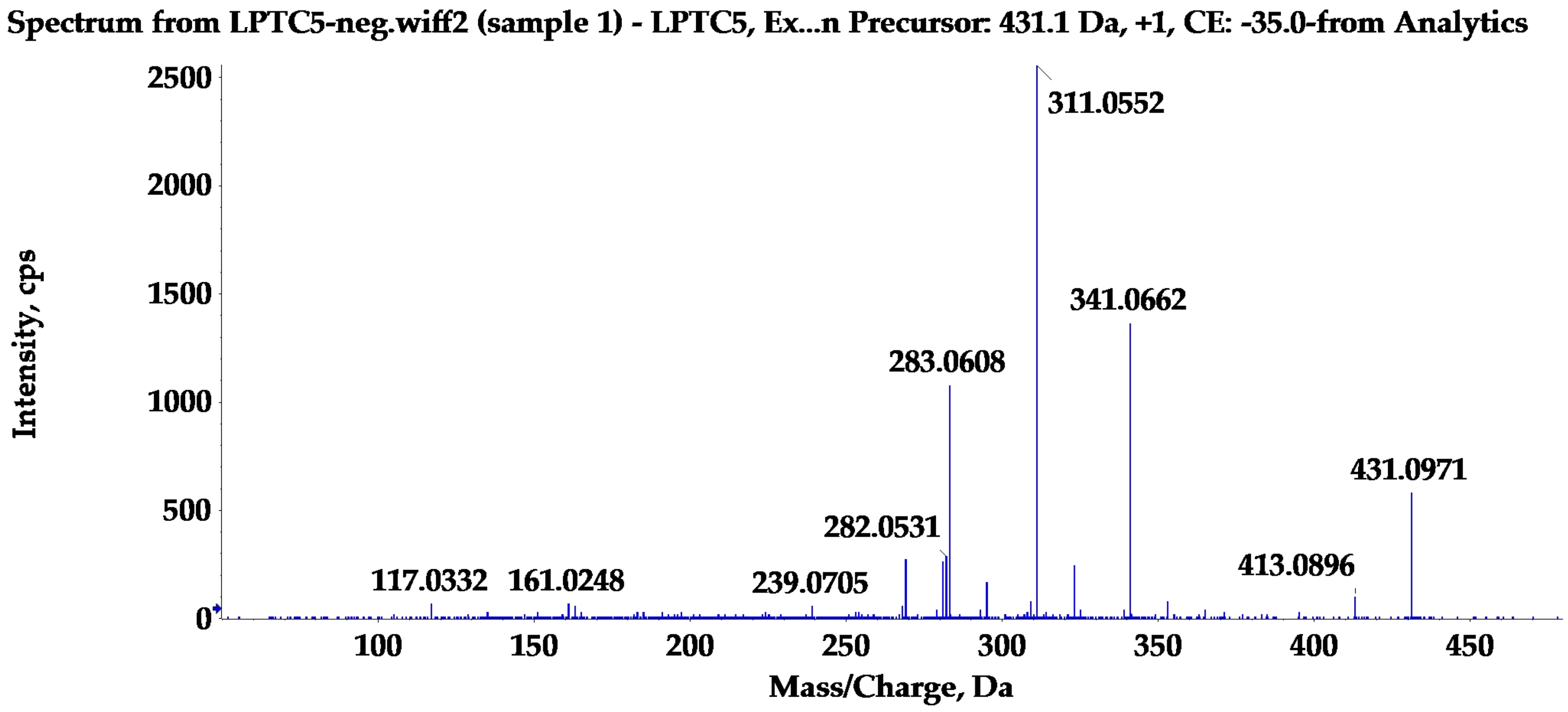

Epigallocatechin 3-O-caffeate

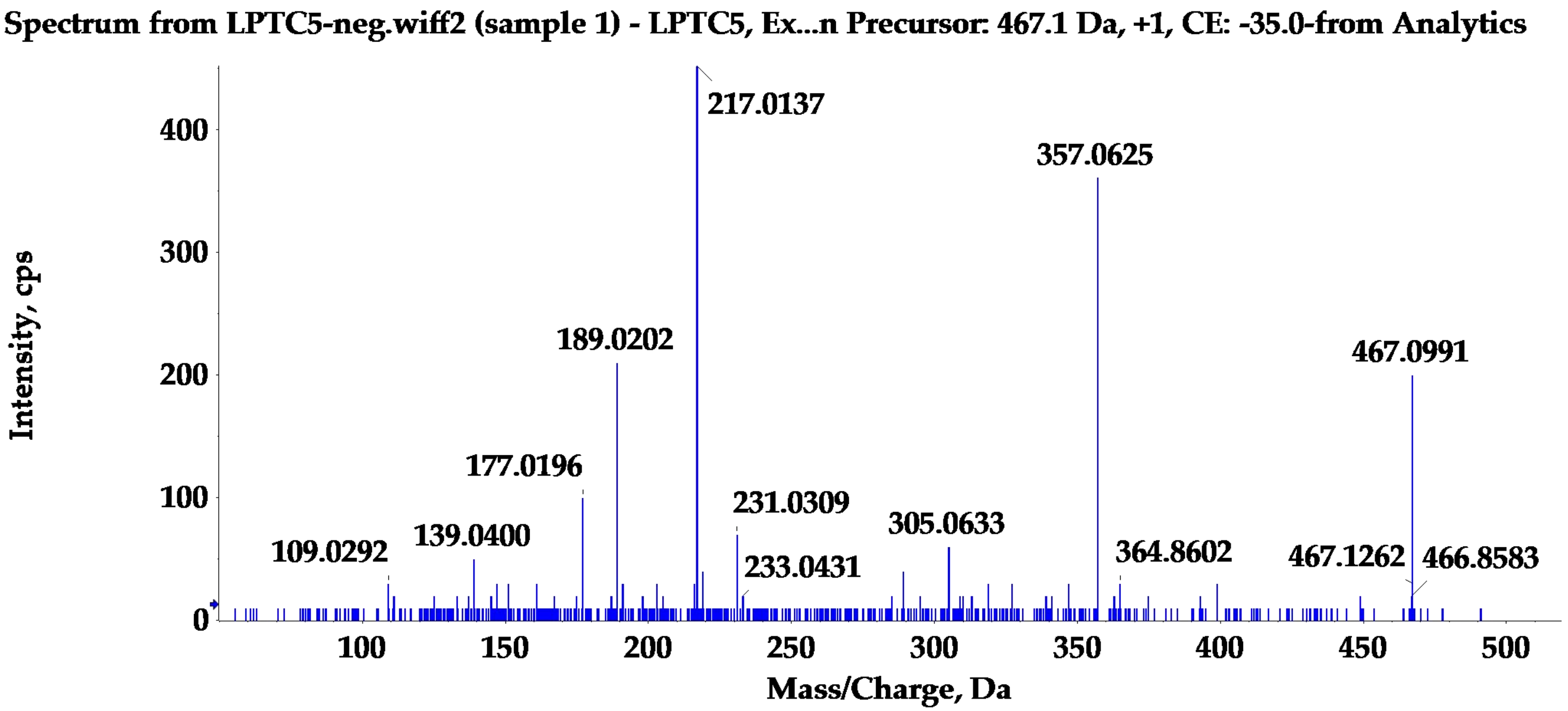

Hyperoside（Quercetin-3-galactoside）

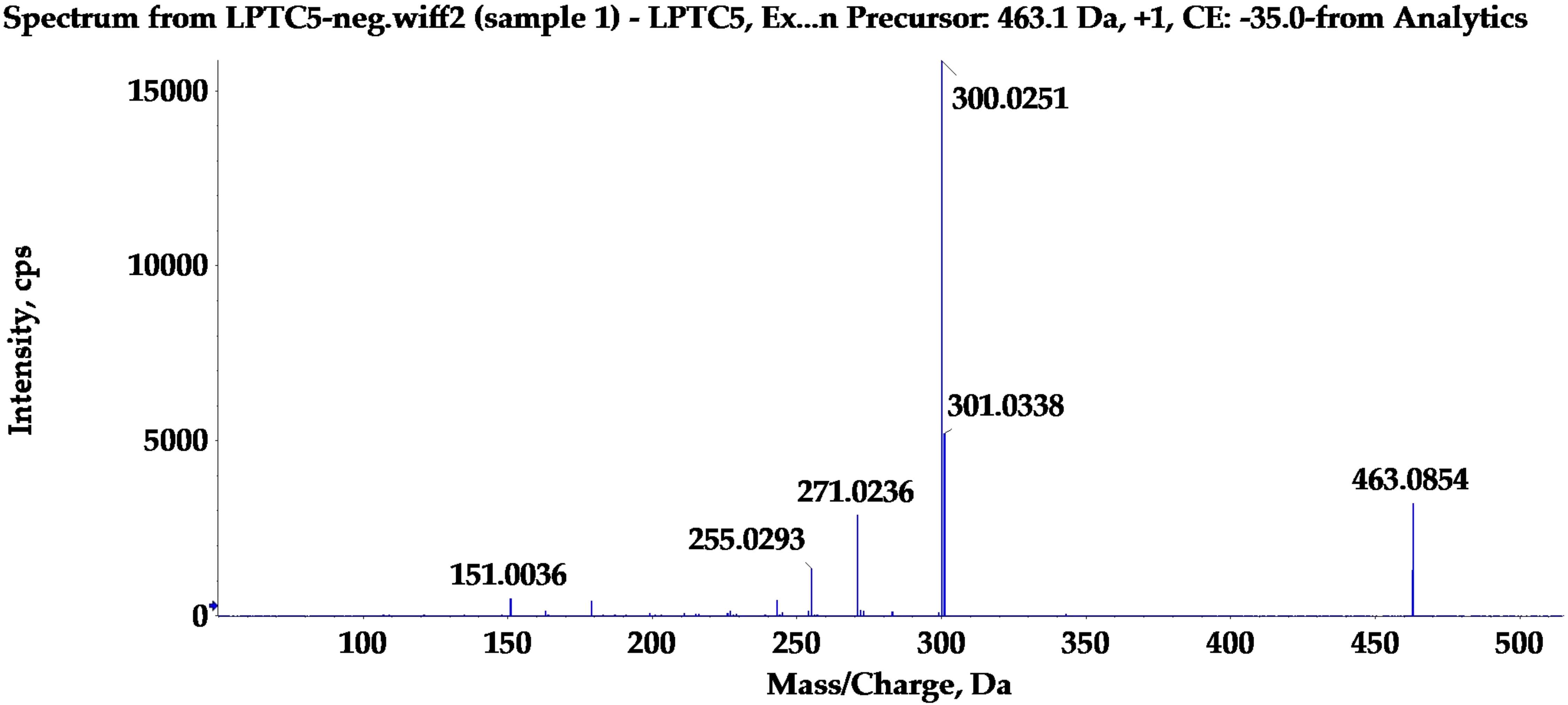

Kaempferol 3-O-(2,6-di-O-alpha-L-rhamnopyranosyl)-beta-D-galactopyranoside

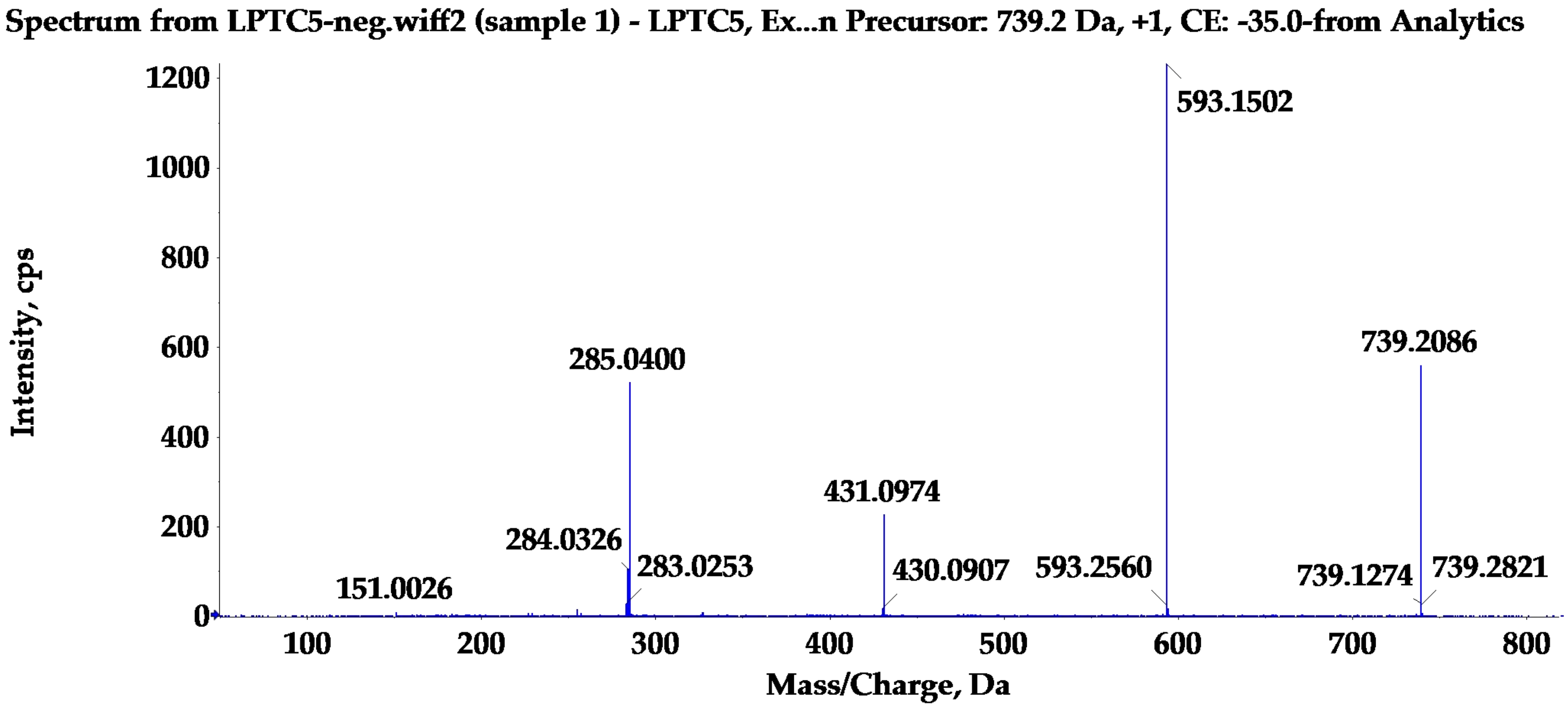

Kaempferol-3-O-rutinoside

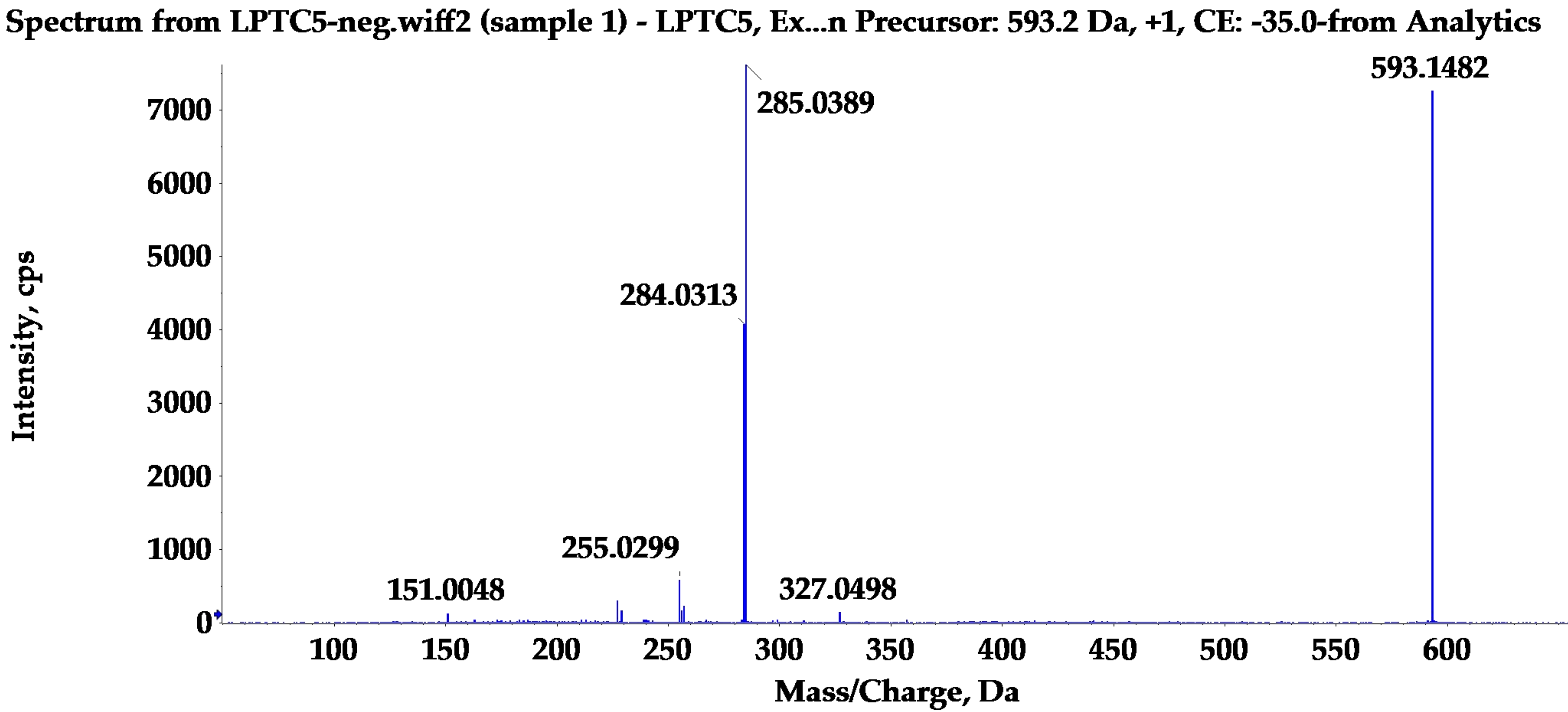

Epigallocatechin 3-O-p-coumarate

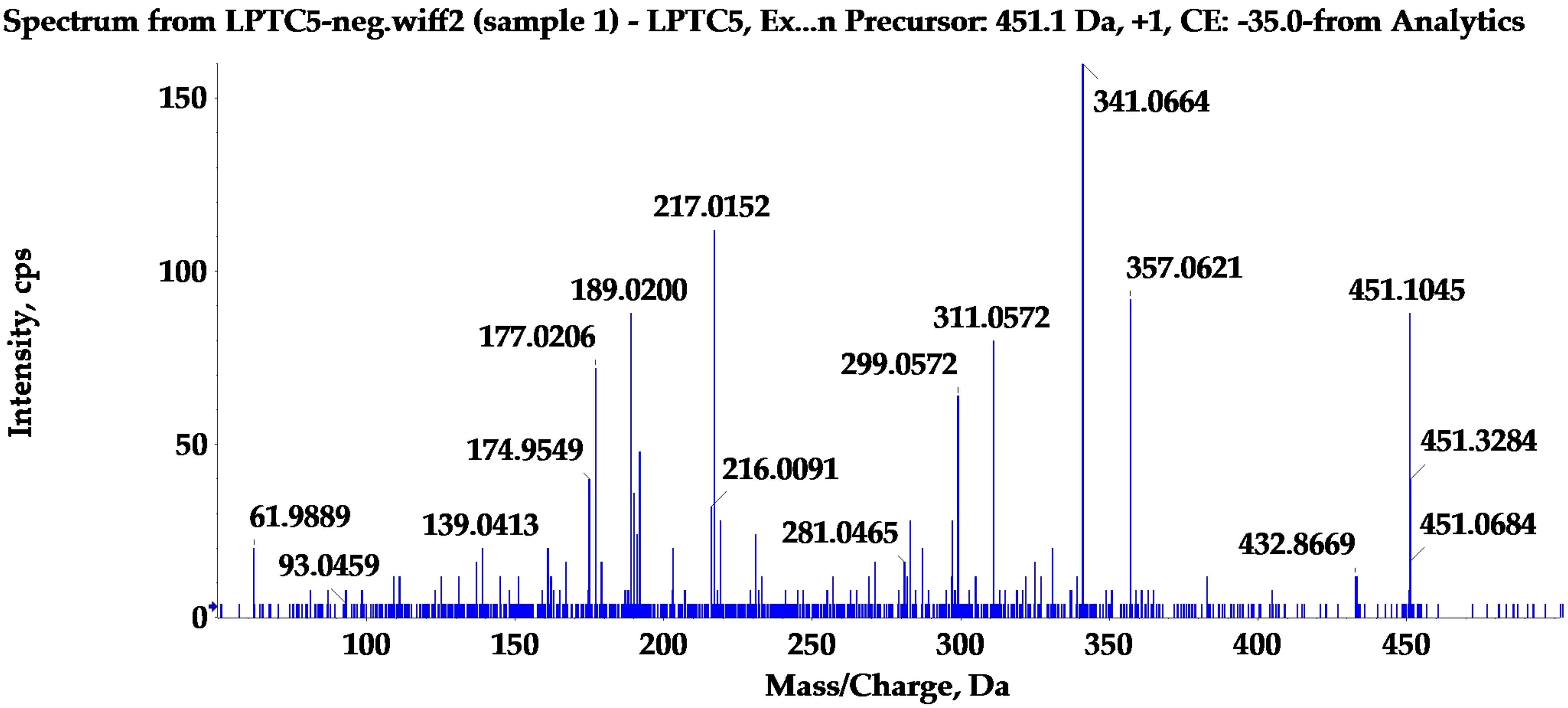

1,2,6,10-Tetrahydroxy-11-methoxy-3,3,12-trimethyl-1,2,3,12-tetrahydro-7H-pyrano[2,3-c]acridin-7-one

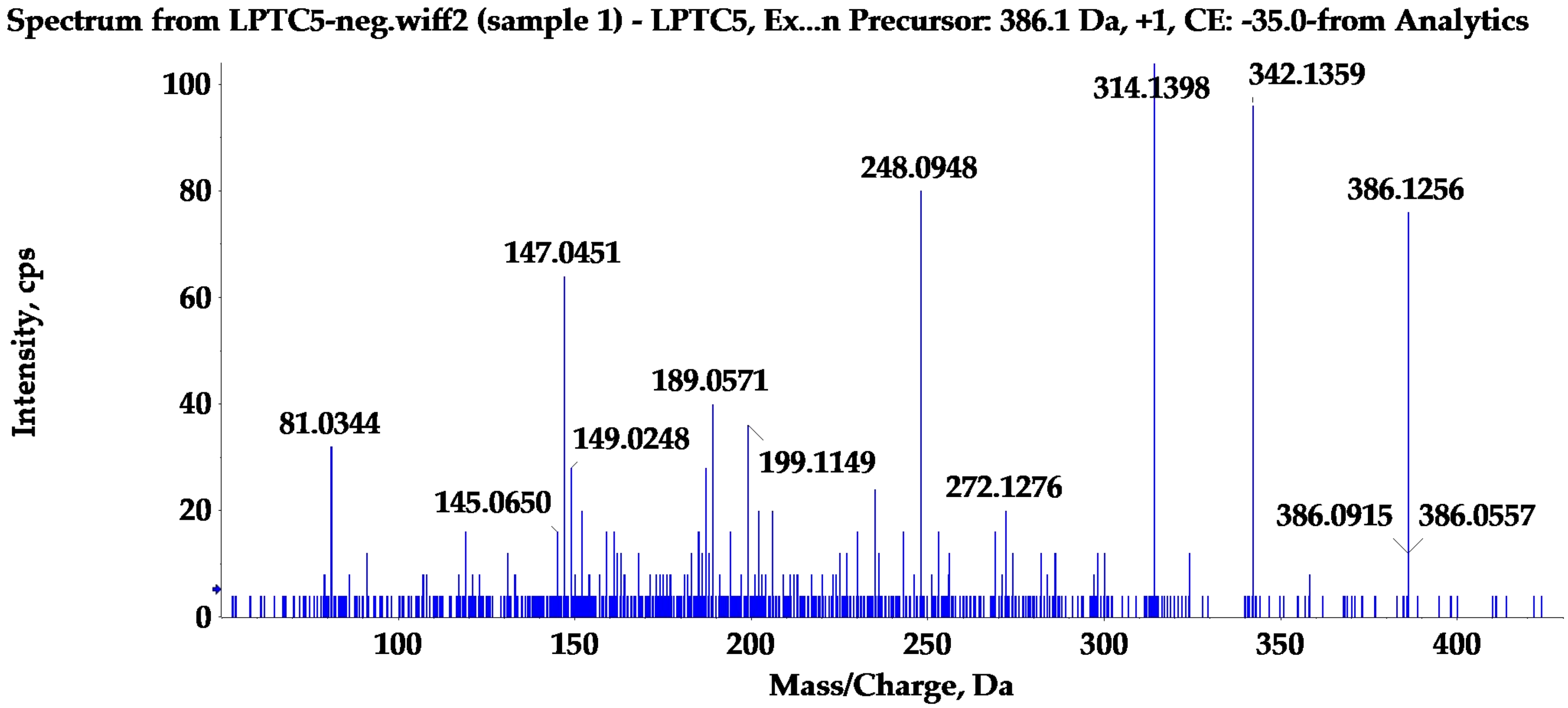

Luteolin-7-O-glucoside

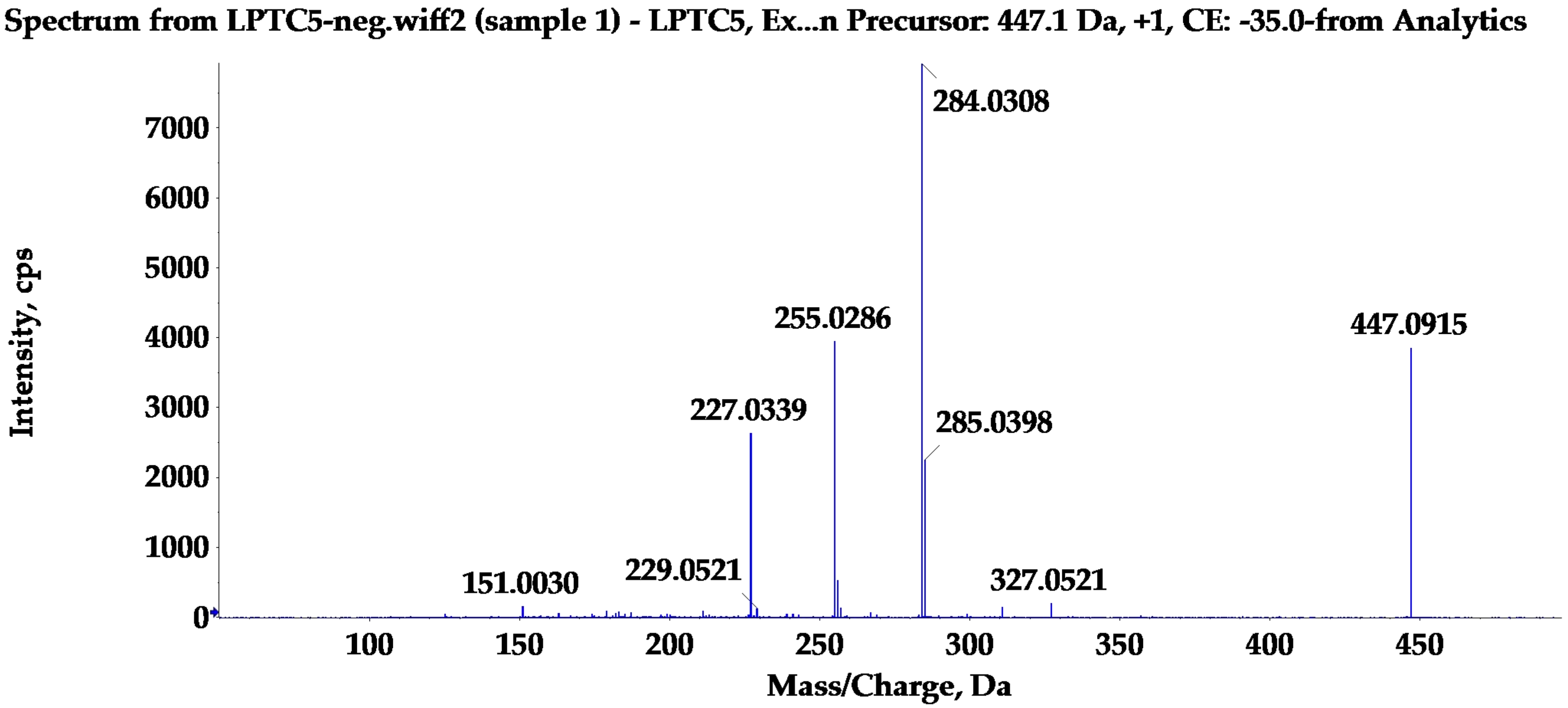

Kaempferol 3-O-(2,6-di-O-alpha-L-rhamnopyranosyl)-beta-D-galactopyranoside

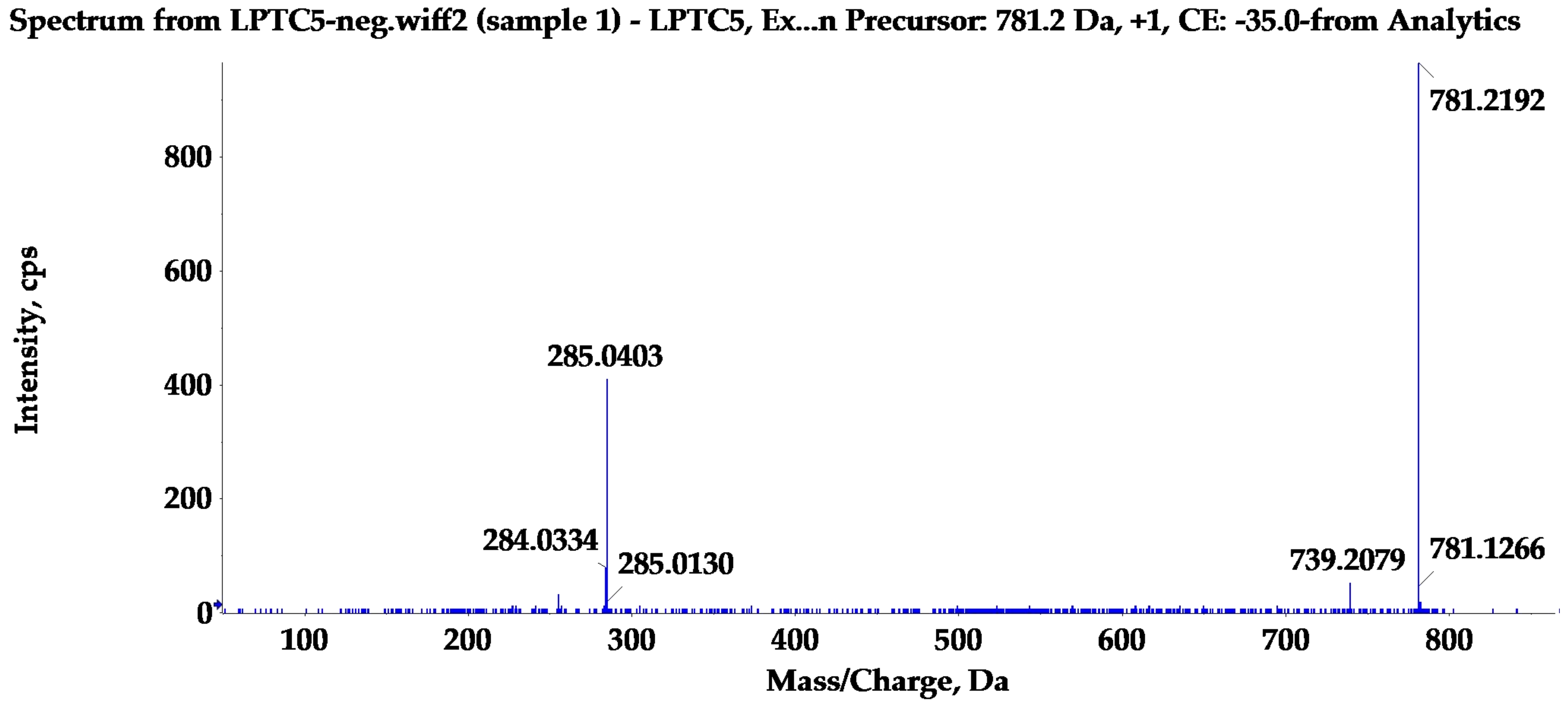

Epigallocatechin 3-O-cinnamate

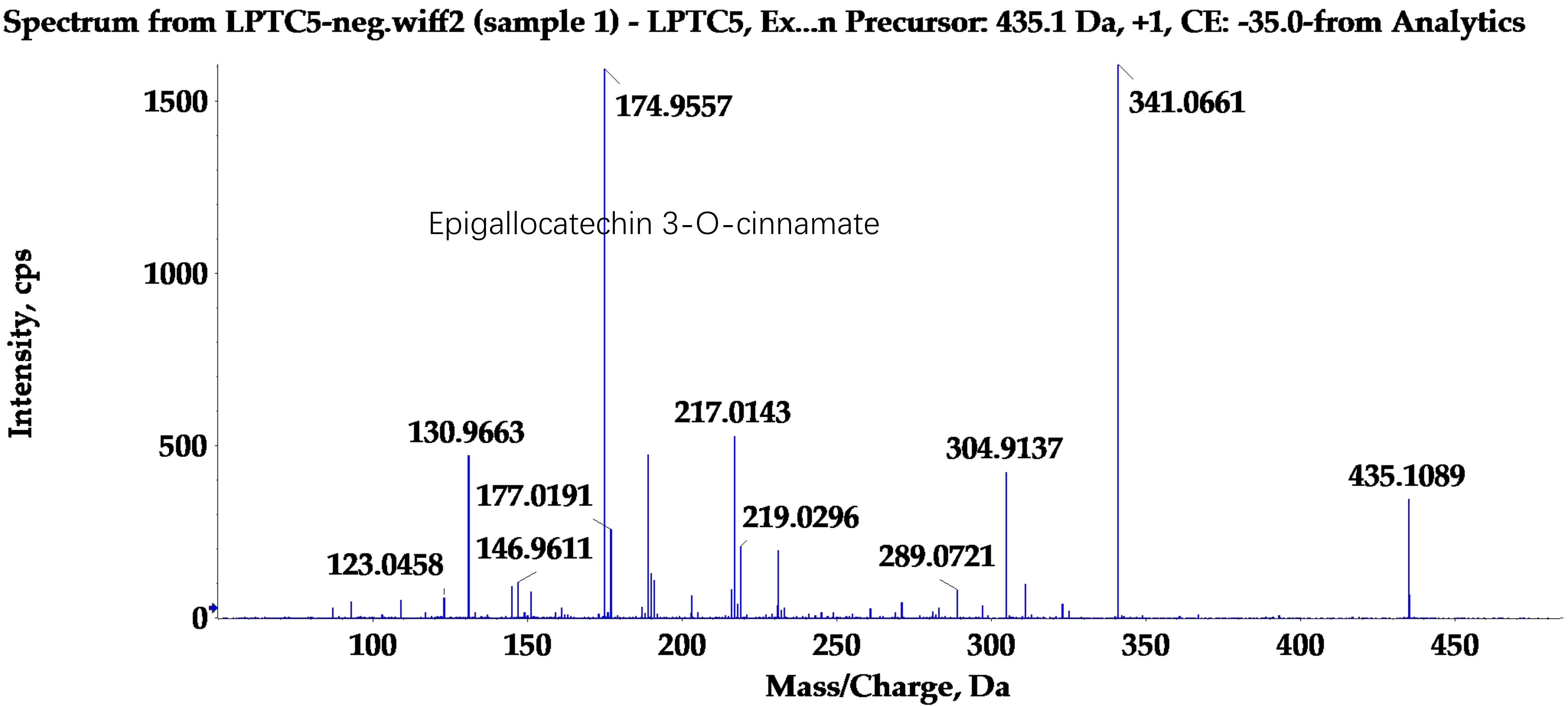

Naringenin

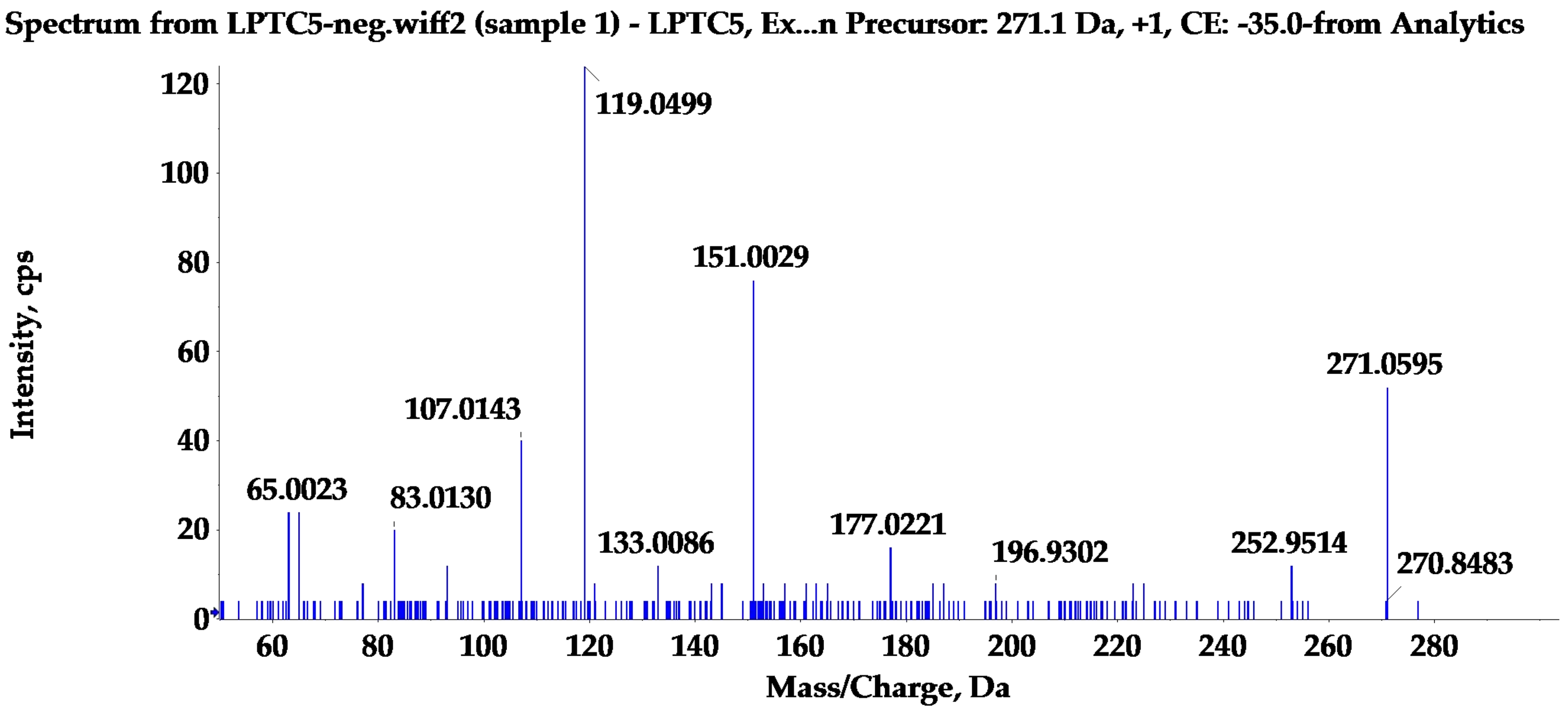

Spectrum from LPTC5-neg.wiff2 (sample 1) - LPTC5, Ex...n Precursor: 271.1 Da, +1, CE: -35.0-from Analytics
Intensity, cps
Mass/Charge, Da
119.0499
151.0029
271.0595
107.0143
65.0023
83.0130
133.0086
177.0221
196.9302
252.9514
270.8483
0
20
40
60
80
100
120
60
80
100
120
140
160
180
200
220
240
260
280

Desgalloyl theasinensin F

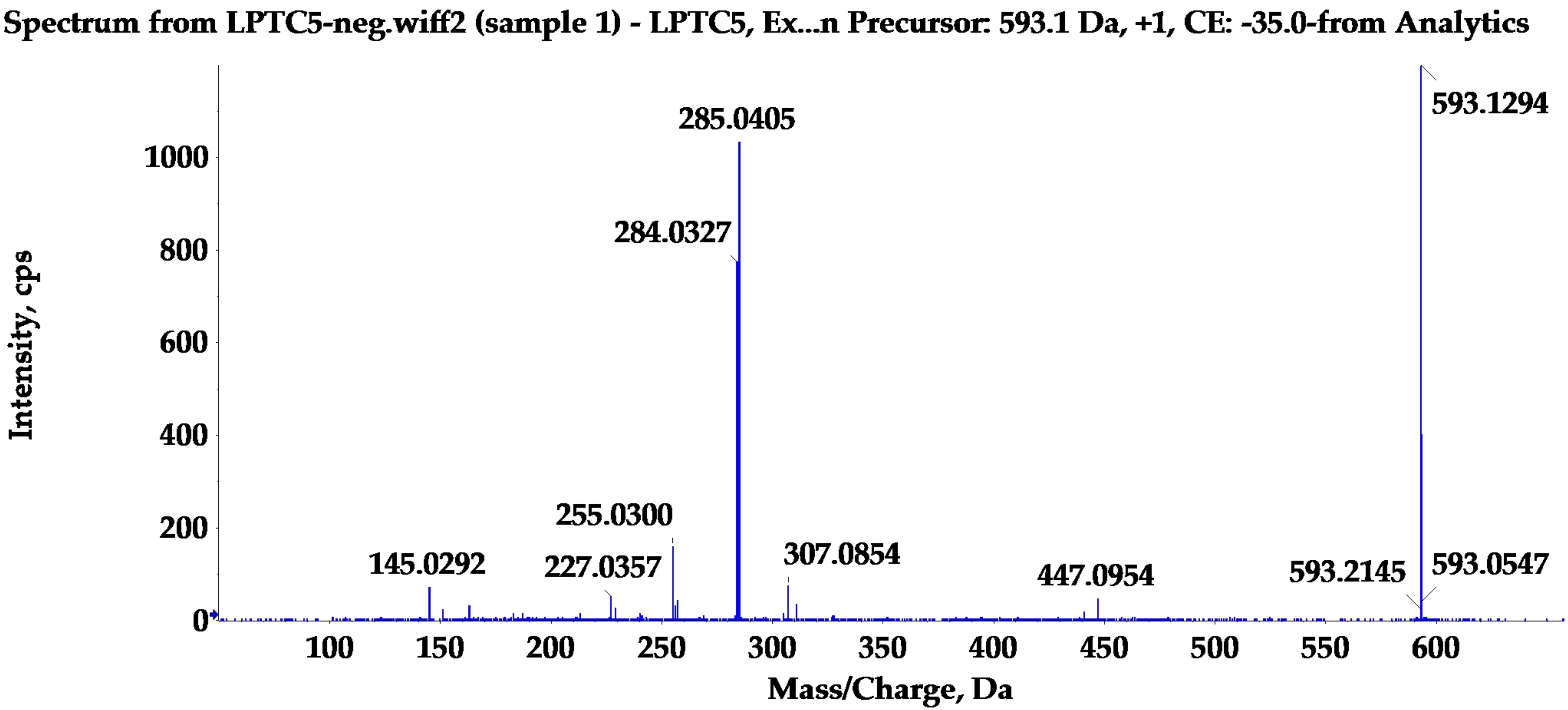

4-Dodecylbenzenesulfonic acid

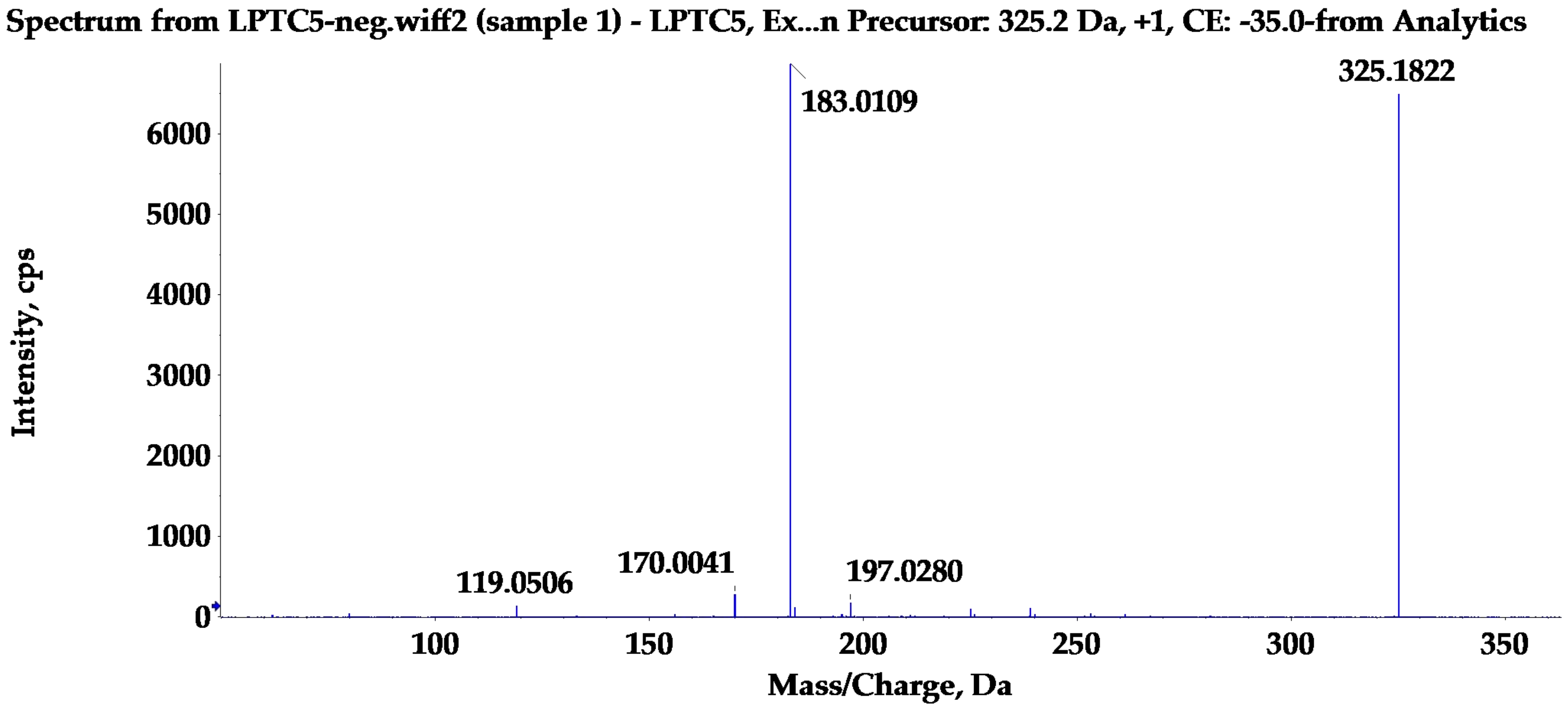

Vitamin B2

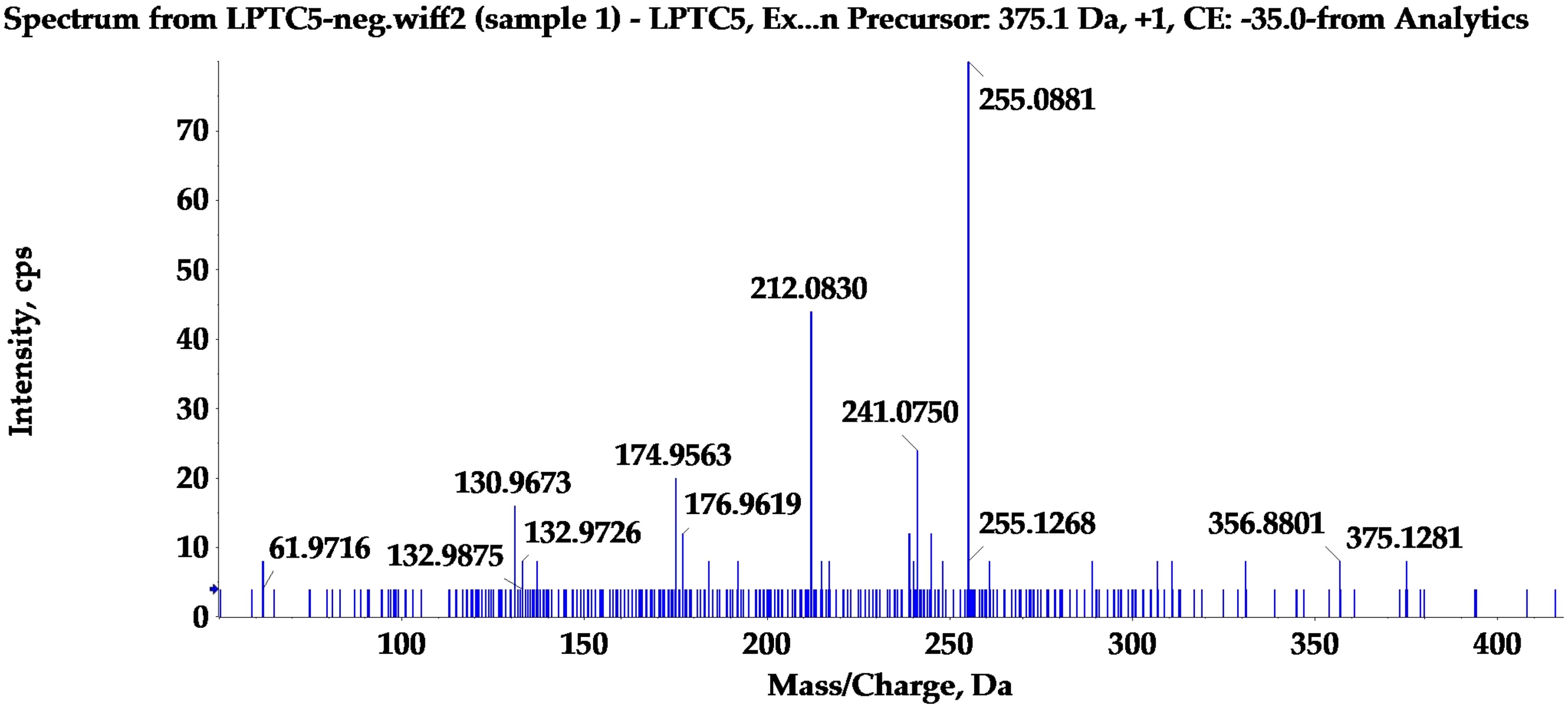

Spectrum from LPTC5-neg.wiff2 (sample 1) - LPTC5, Ex...n Precursor: 375.1 Da, +1, CE: -35.0-from Analytics
Intensity, cps
Mass/Charge, Da
255.0881
212.0830
241.0750
174.9563
130.9673
176.9619
132.9726
255.1268
356.8801
375.1281
61.9716
132.9875
0
10
20
30
40
50
60
70
100
150
200
250
300
350
400

Linoleic acid [Smart Confirmation]

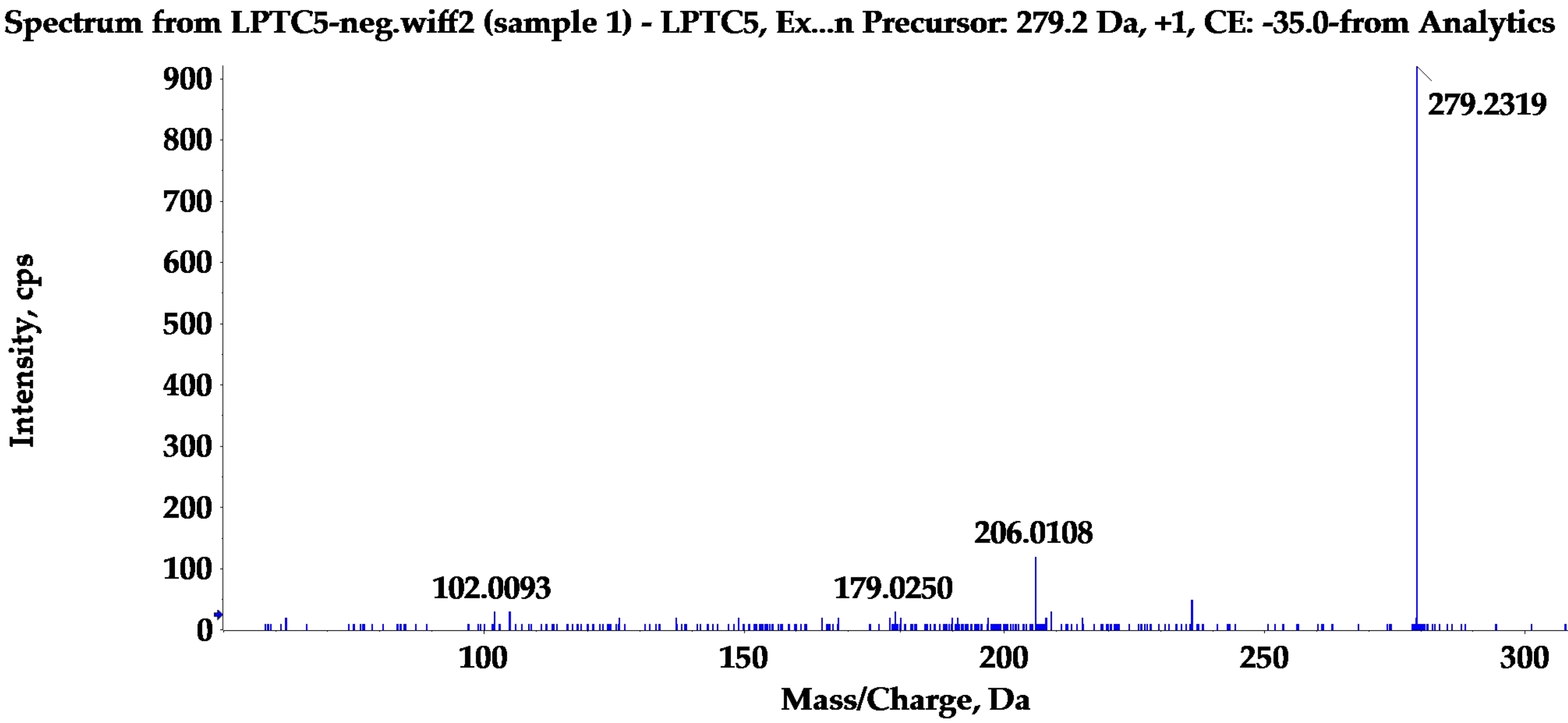

Palmitic acid [Smart Confirmation]

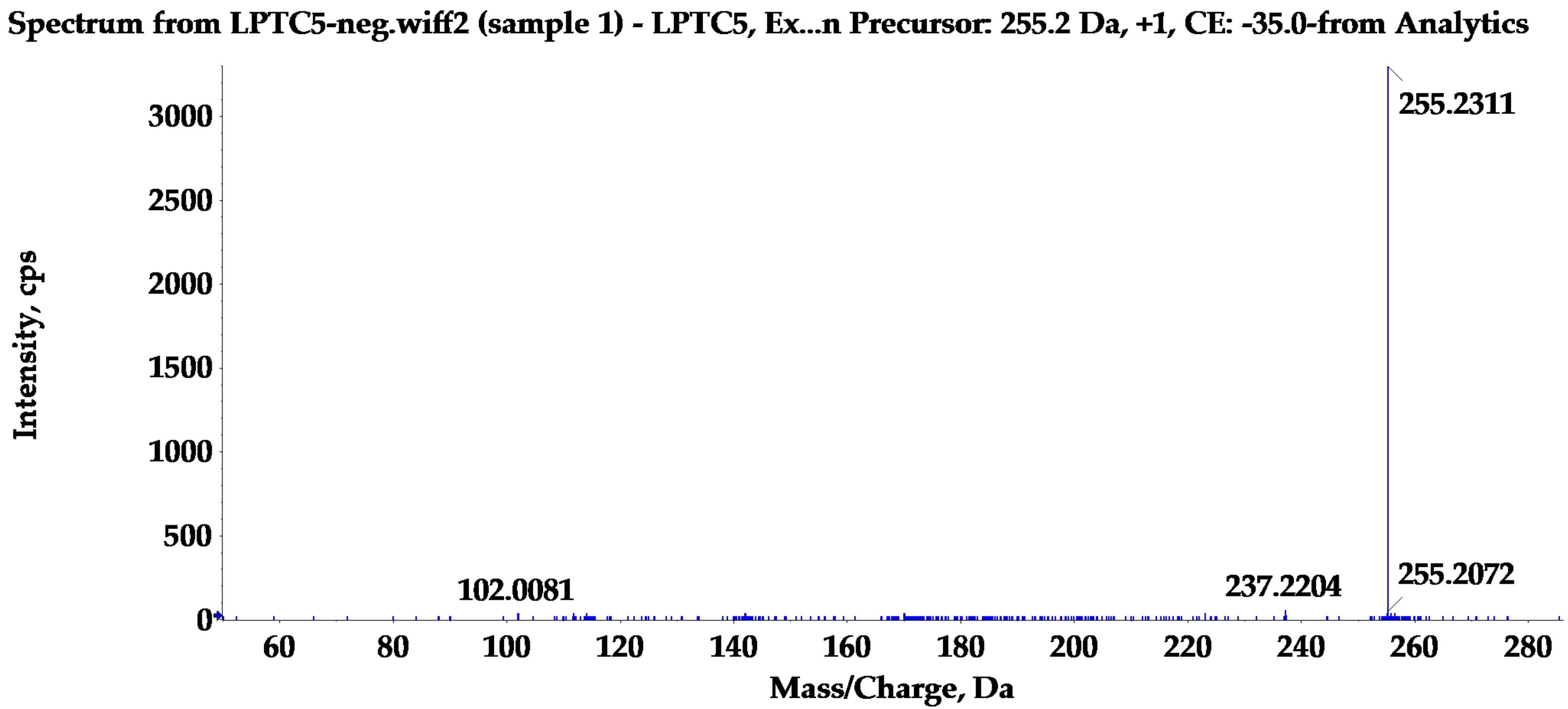

Benzoic acid

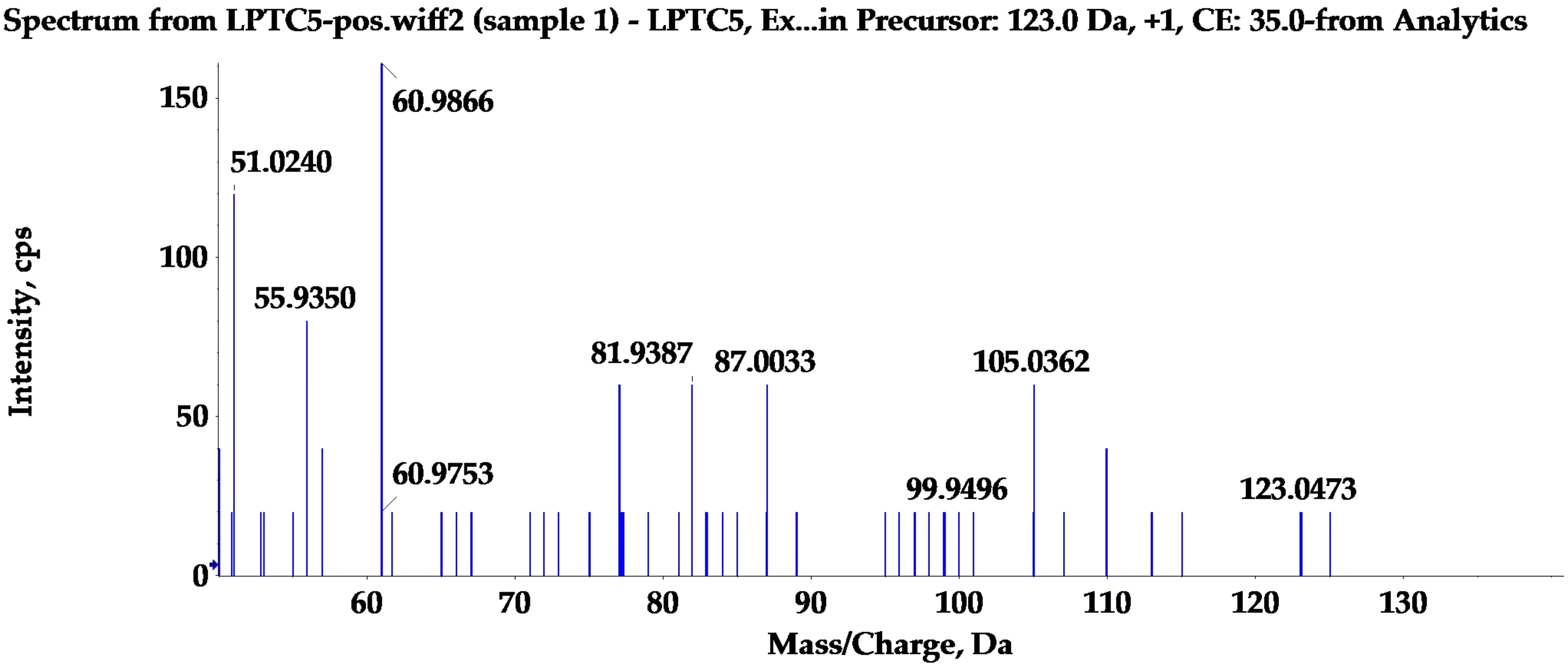

L-Pyroglutamicacid

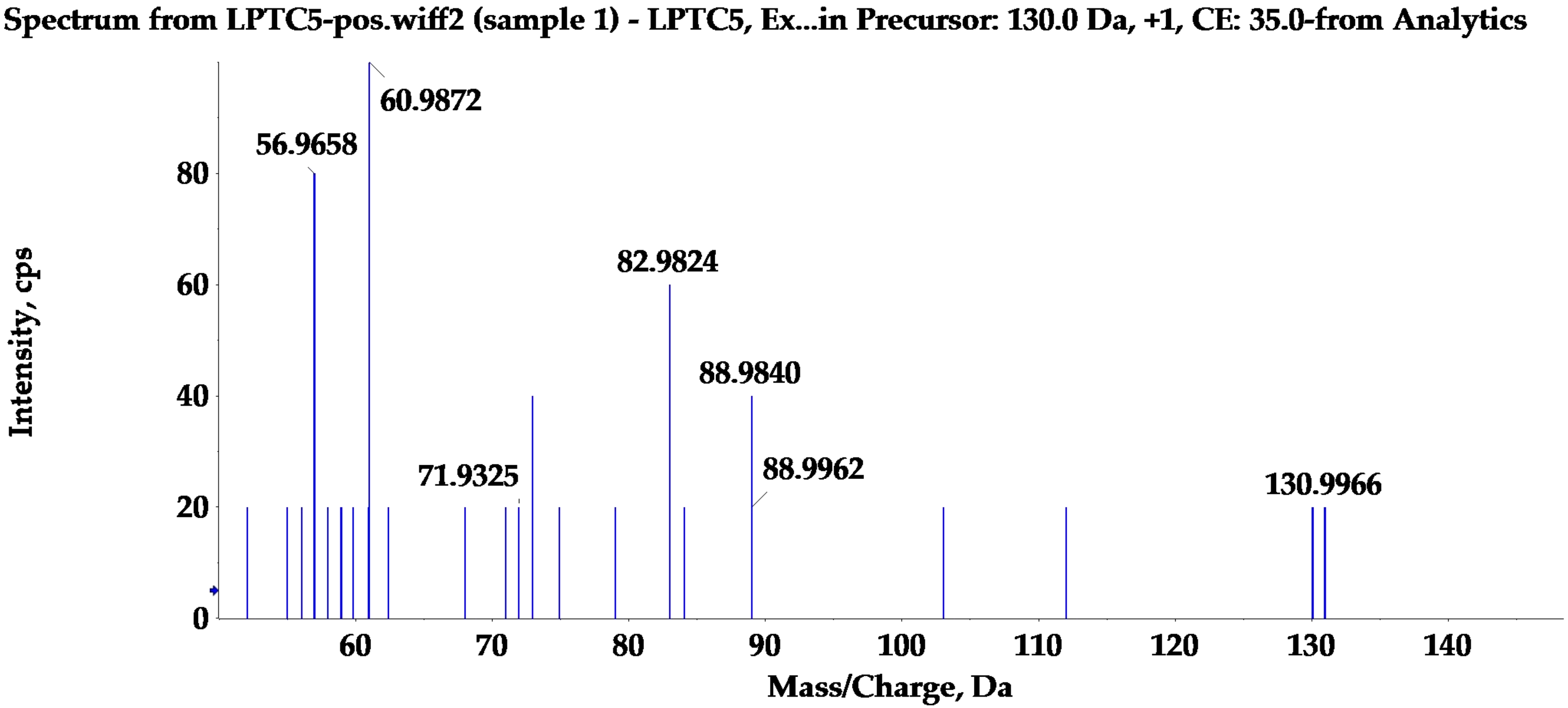

Adenosine

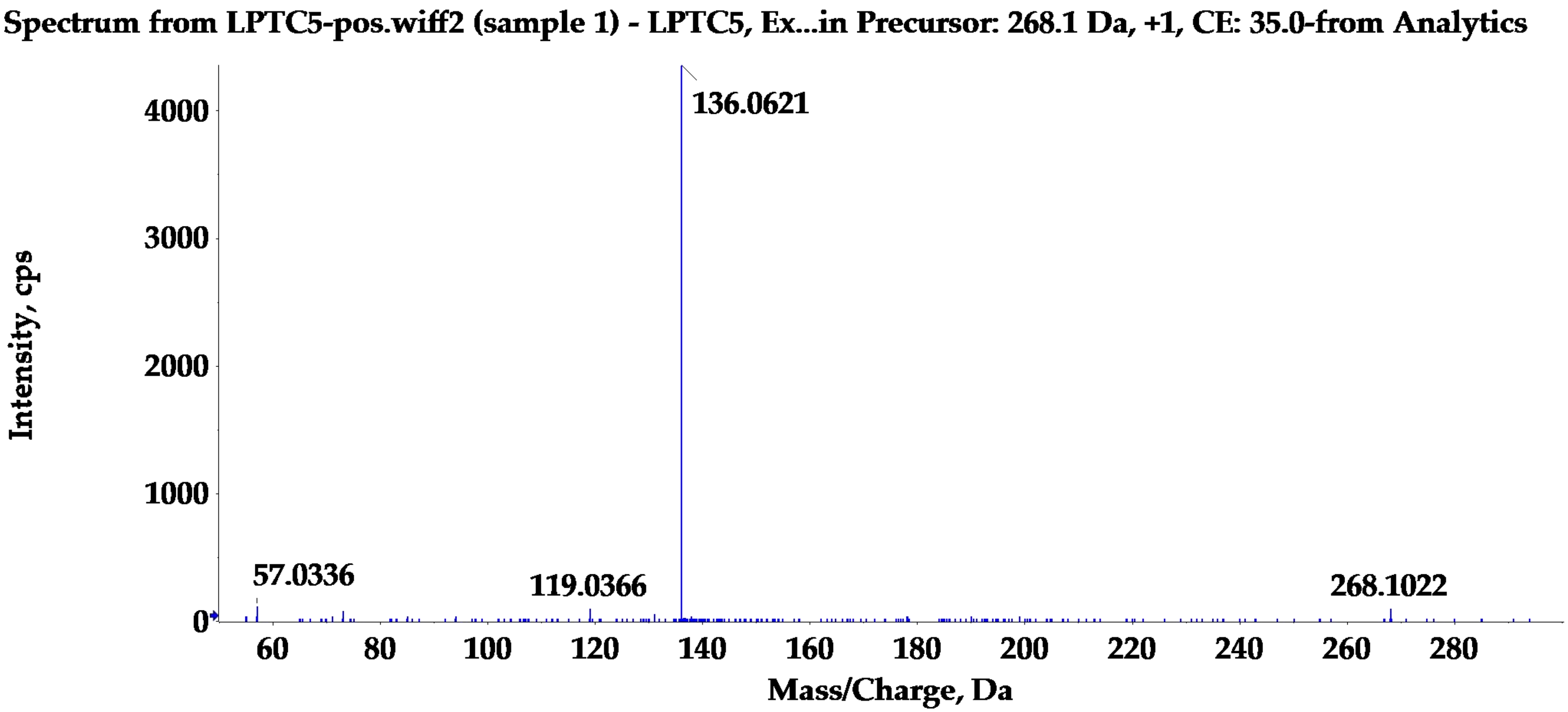
Spectrum from LPTC5-pos.wiff2 (sample 1) - LPTC5, Ex...in Precursor: 268.1 Da, +1, CE: 35.0-from Analytics
Intensity, cps
4000
3000
2000
1000
0
136.0621
57.0336
119.0366
268.1022
60
80
100
120
140
160
180
200
220
240
260
280
Mass/Charge, Da

hypoxanthine

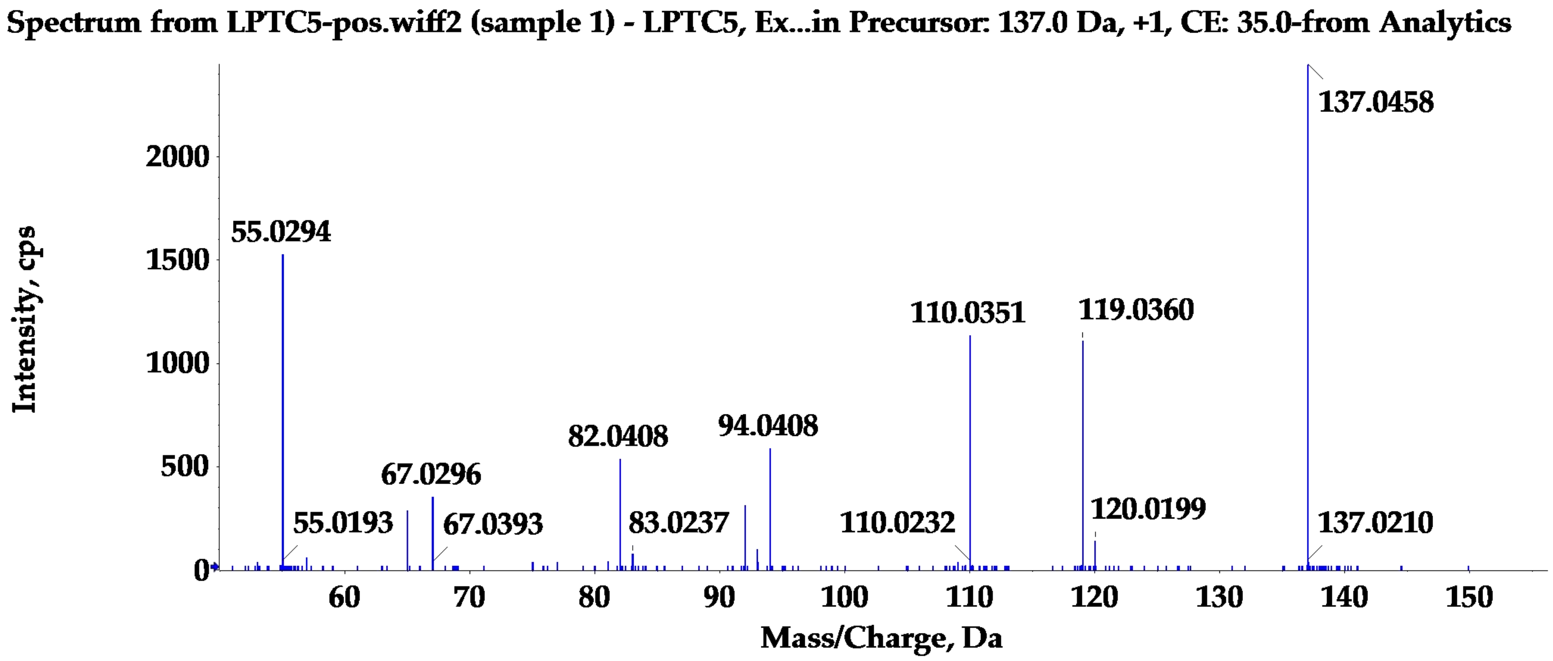

2-Amino-1,3,4-octadecanetriol

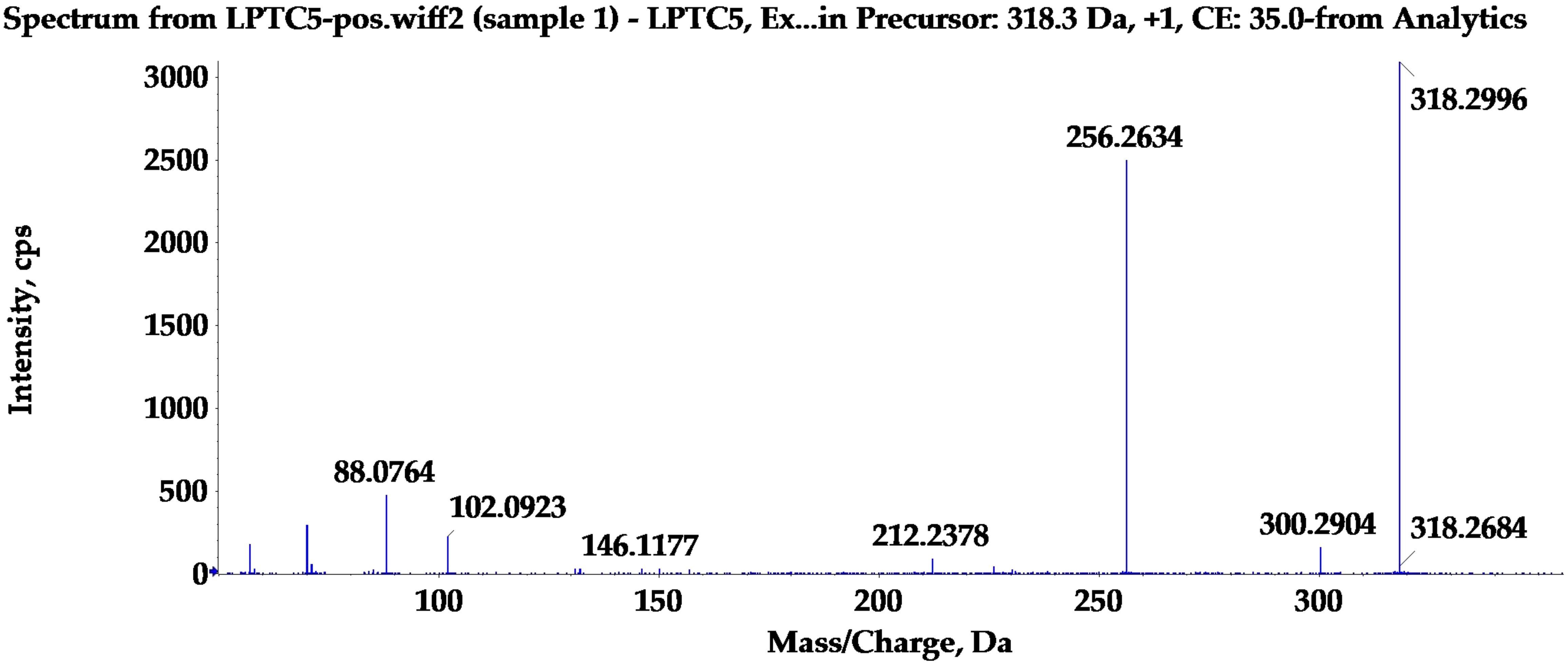

Guanine

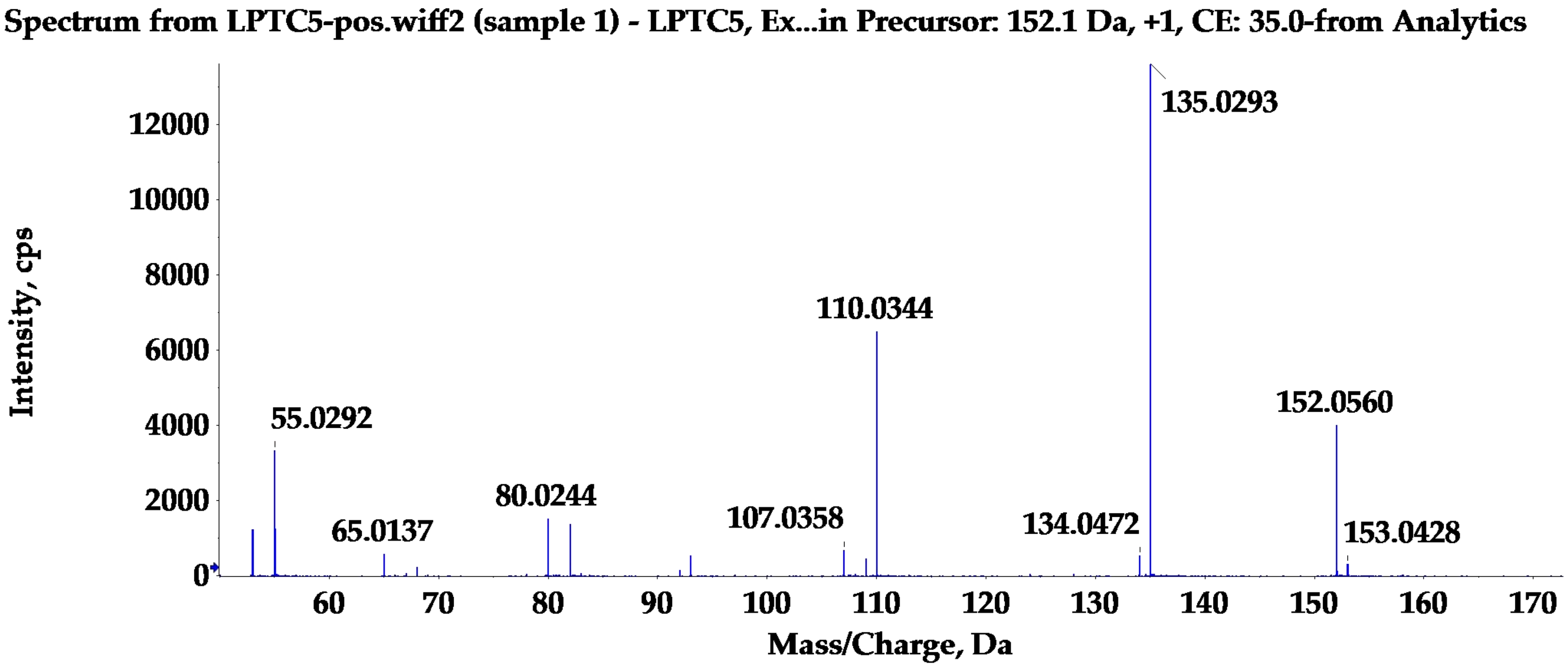

Spectrum from LPTC5-pos.wiff2 (sample 1) - LPTC5, Ex...in Precursor: 152.1 Da, +1, CE: 35.0-from Analytics
Intensity, cps
Mass/Charge, Da
135.0293
110.0344
152.0560
55.0292
80.0244
65.0137
107.0358
134.0472
153.0428
0
2000
4000
6000
8000
10000
12000
60
70
80
90
100
110
120
130
140
150
160
170

Stearic acid amide

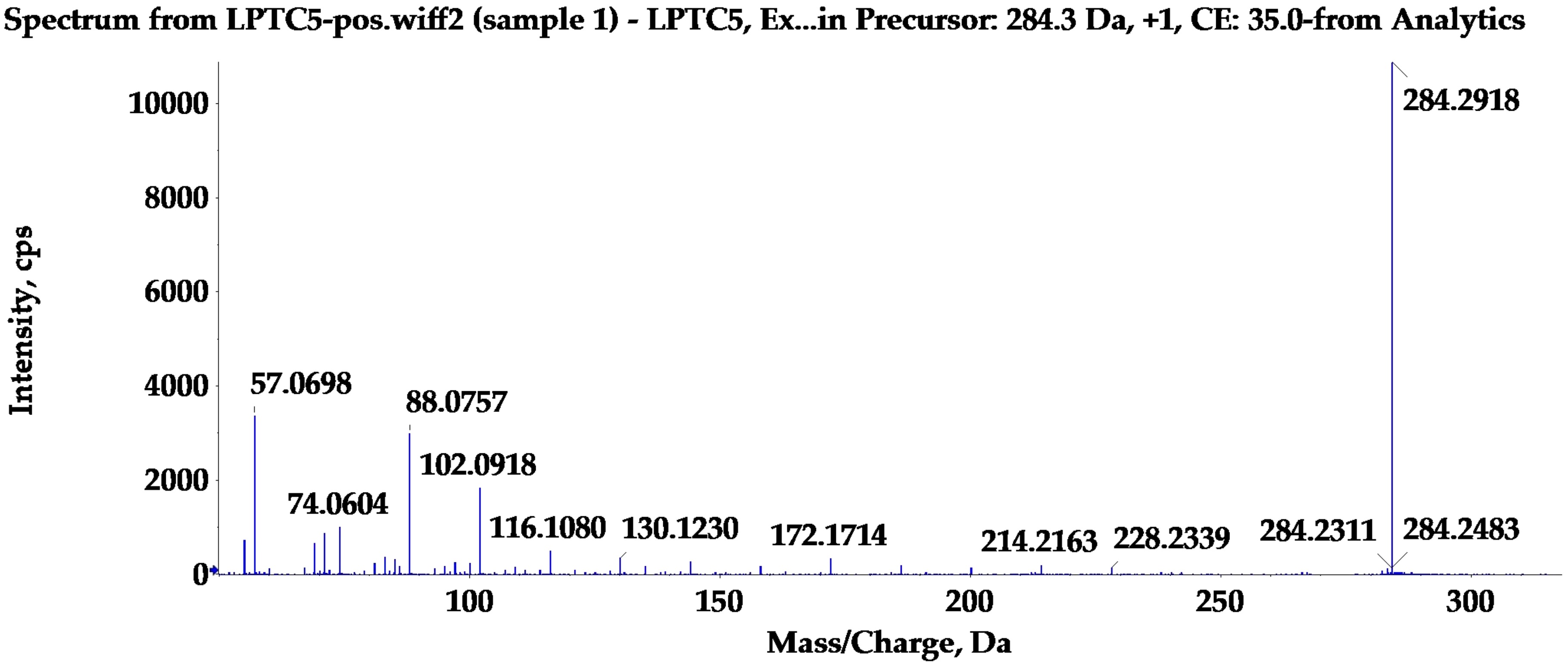

Theobromine

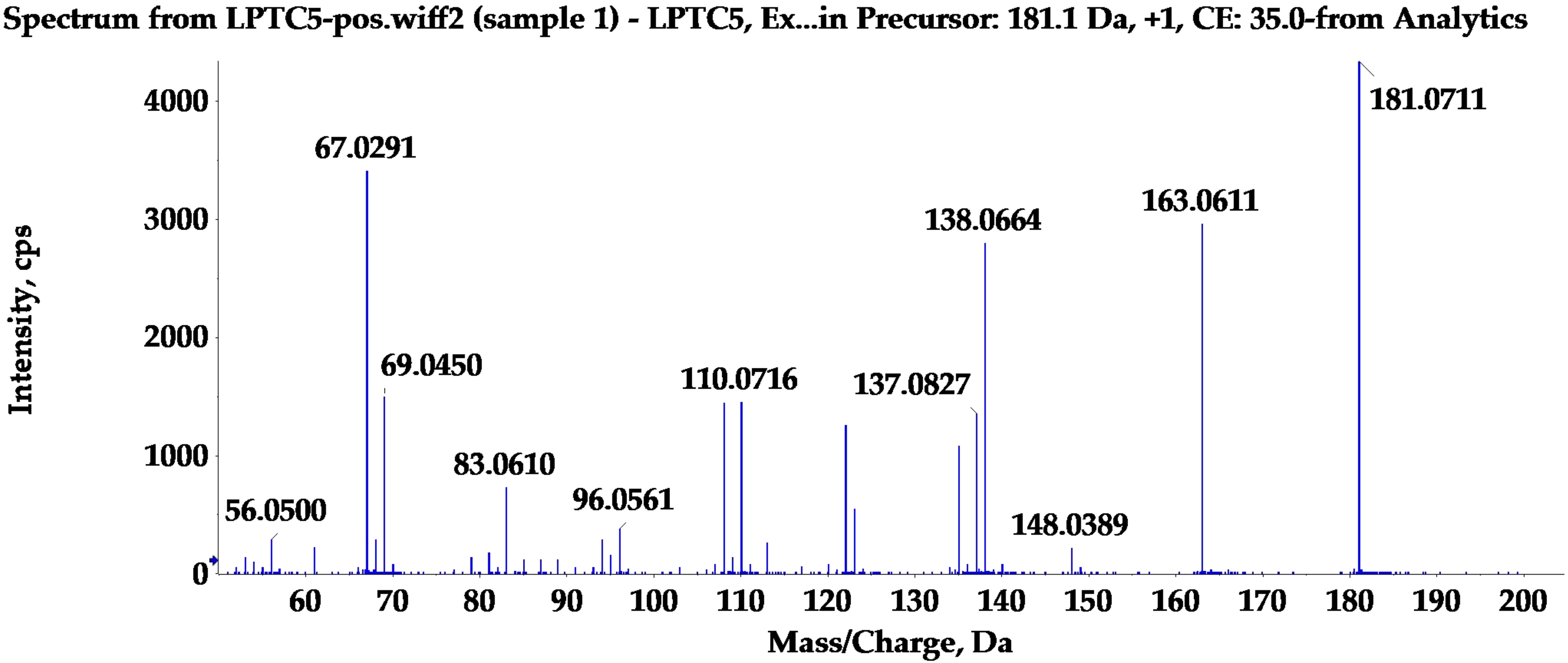

Palmitamide

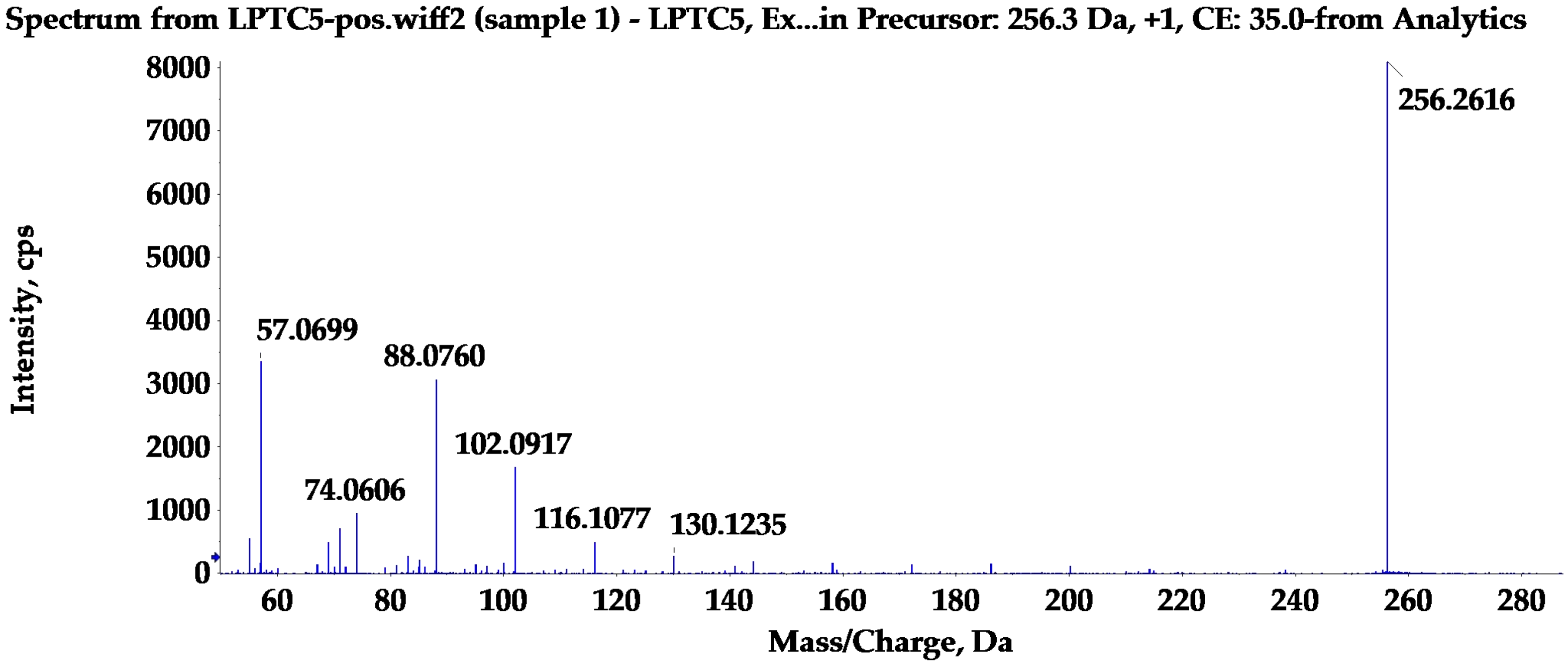

Oleamide

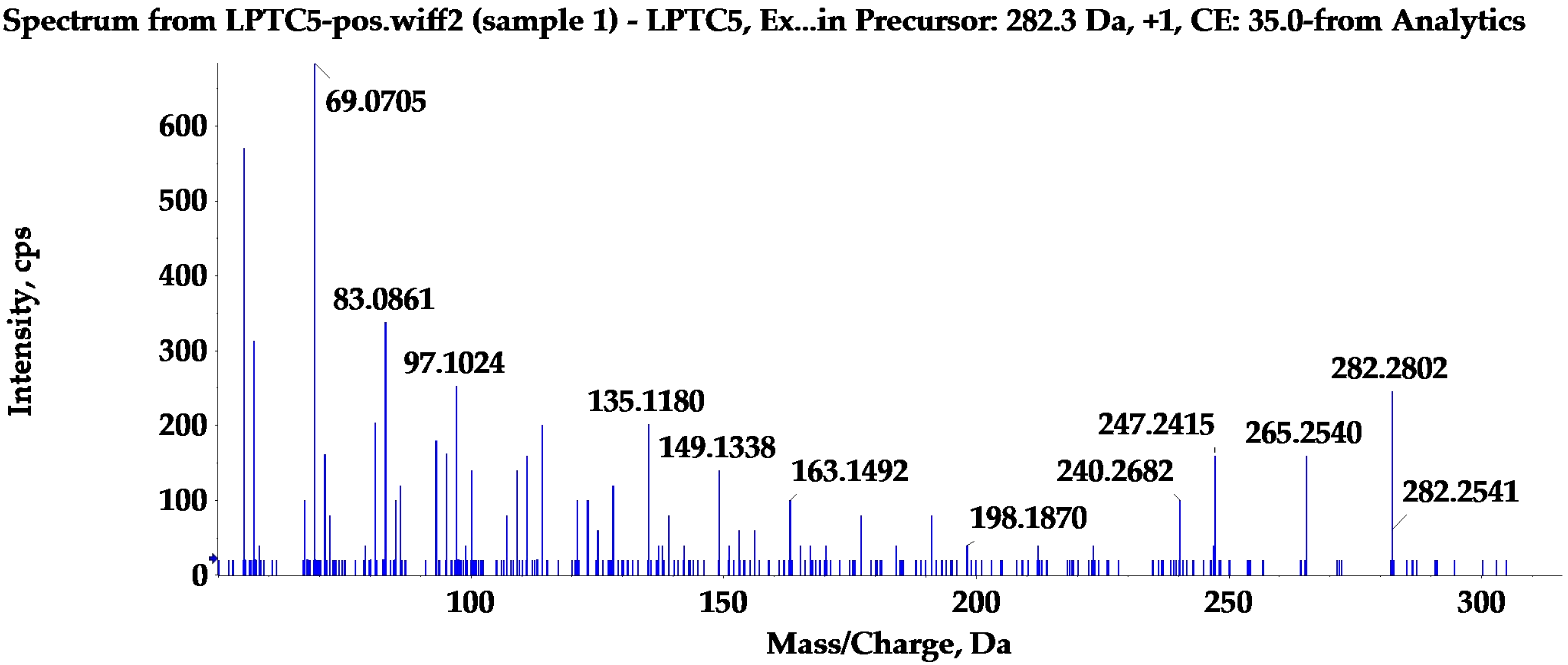

Spectrum from LPTC5-pos.wiff2 (sample 1) - LPTC5, Ex...in Precursor: 282.3 Da, +1, CE: 35.0-from Analytics
Intensity, cps
Mass/Charge, Da
69.0705
83.0861
97.1024
135.1180
149.1338
163.1492
198.1870
240.2682
247.2415
265.2540
282.2802
282.2541
0
100
200
300
400
500
600
100
150
200
250
300

Caffeine

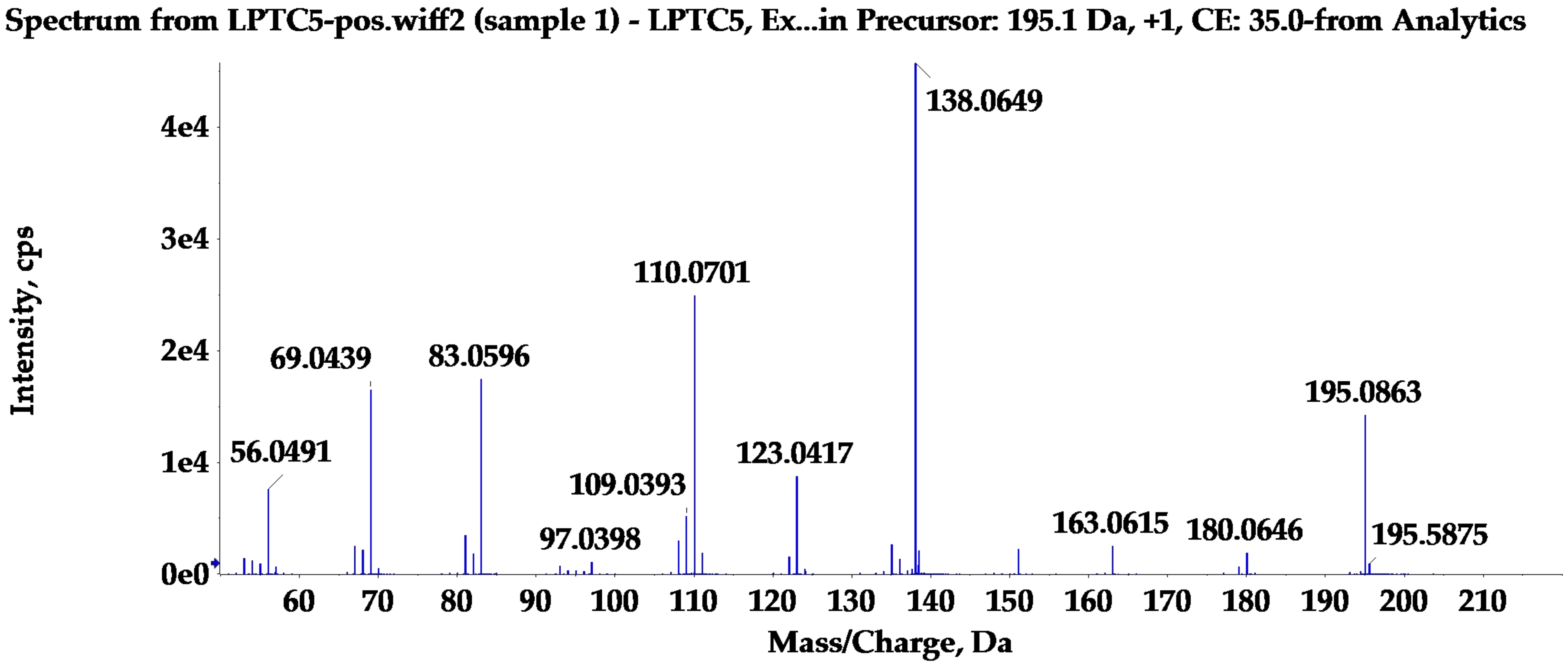

Spectrum from LPTC5-pos.wiff2 (sample 1) - LPTC5, Ex...in Precursor: 195.1 Da, +1, CE: 35.0-from Analytics
Intensity, cps
4e4
3e4
2e4
1e4
0e0
138.0649
110.0701
69.0439
83.0596
195.0863
56.0491
123.0417
109.0393
97.0398
163.0615
180.0646
195.5875
60
70
80
90
100
110
120
130
140
150
160
170
180
190
200
210
Mass/Charge, Da

D-Ascorbic Acid

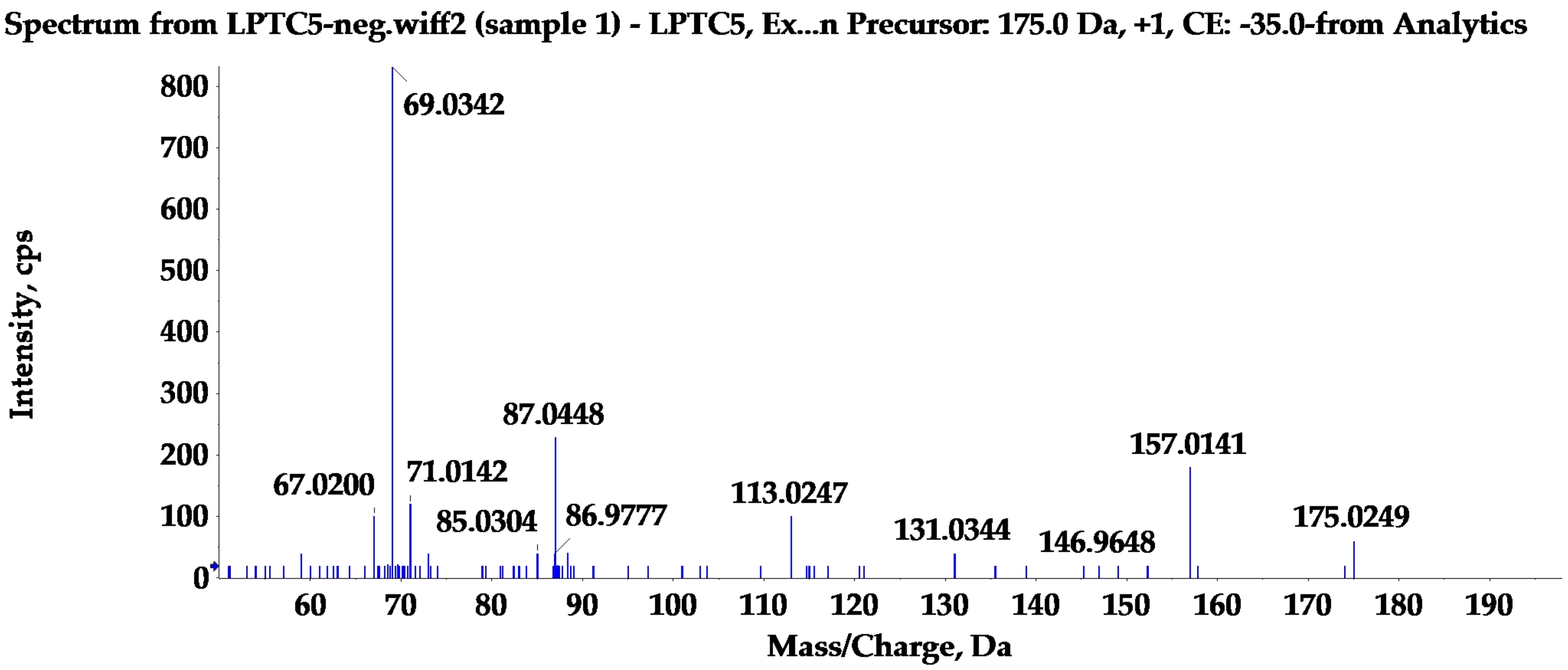

Paeonol

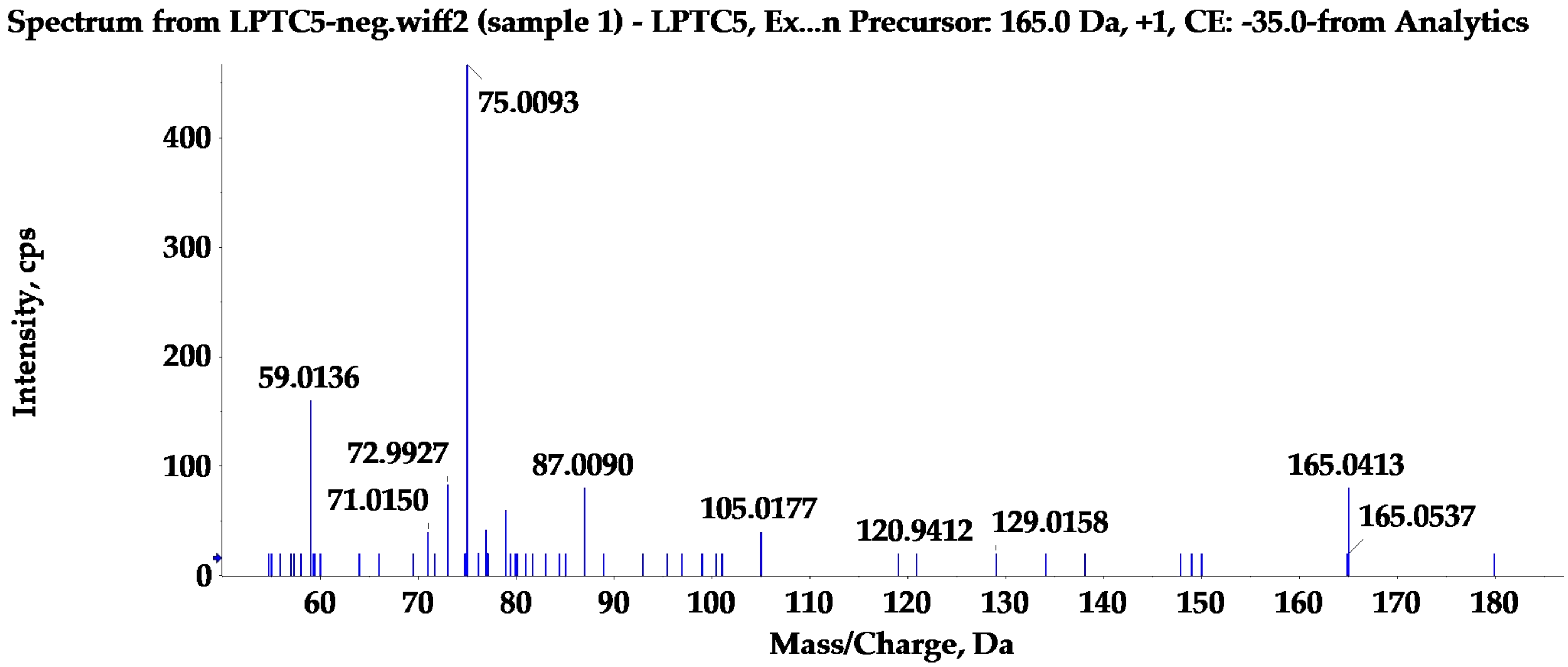
Spectrum from LPTC5-neg.wiff2 (sample 1) - LPTC5, Ex...n Precursor: 165.0 Da, +1, CE: -35.0-from Analytics
Intensity, cps
Mass/Charge, Da
75.0093
59.0136
72.9927
71.0150
87.0090
105.0177
120.9412
129.0158
165.0413
165.0537
0
100
200
300
400
60
70
80
90
100
110
120
130
140
150
160
170
180

Isolariciresinol

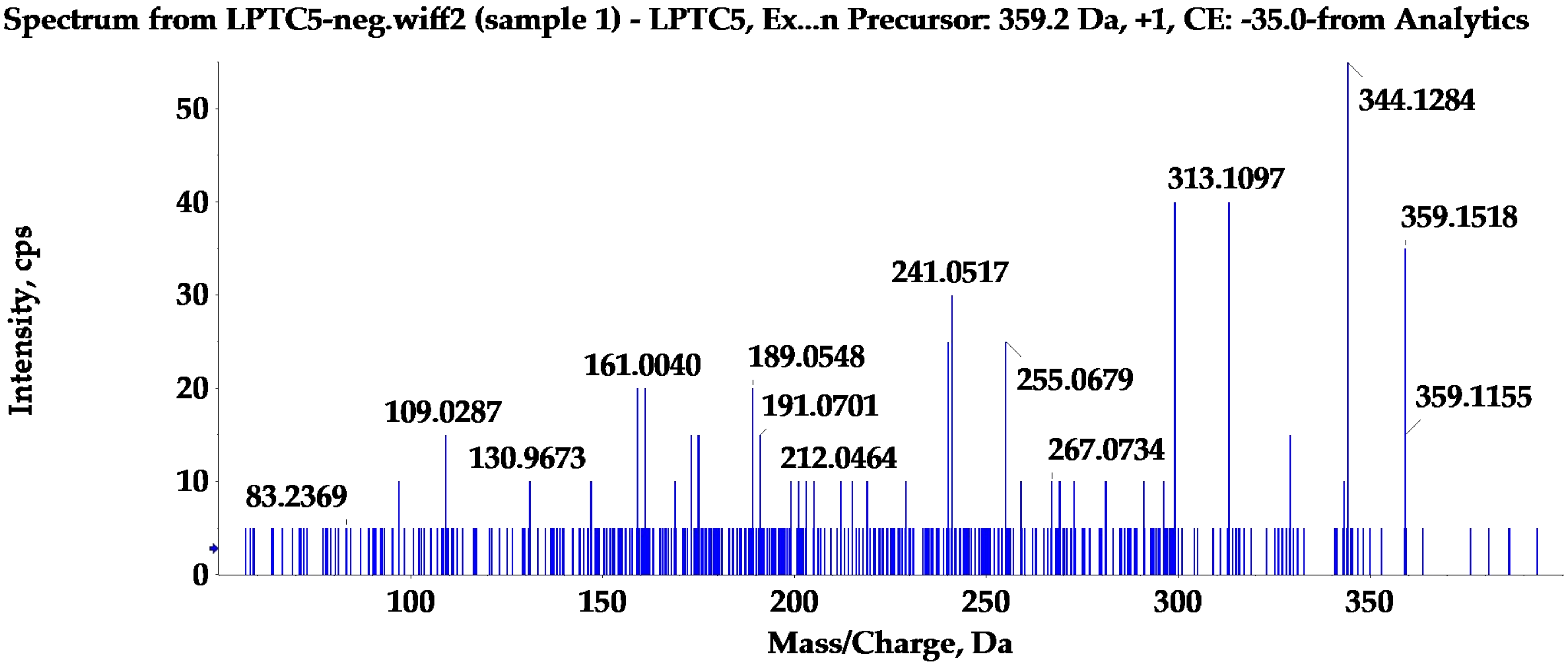

Kaempferol-3-O-rutinoside

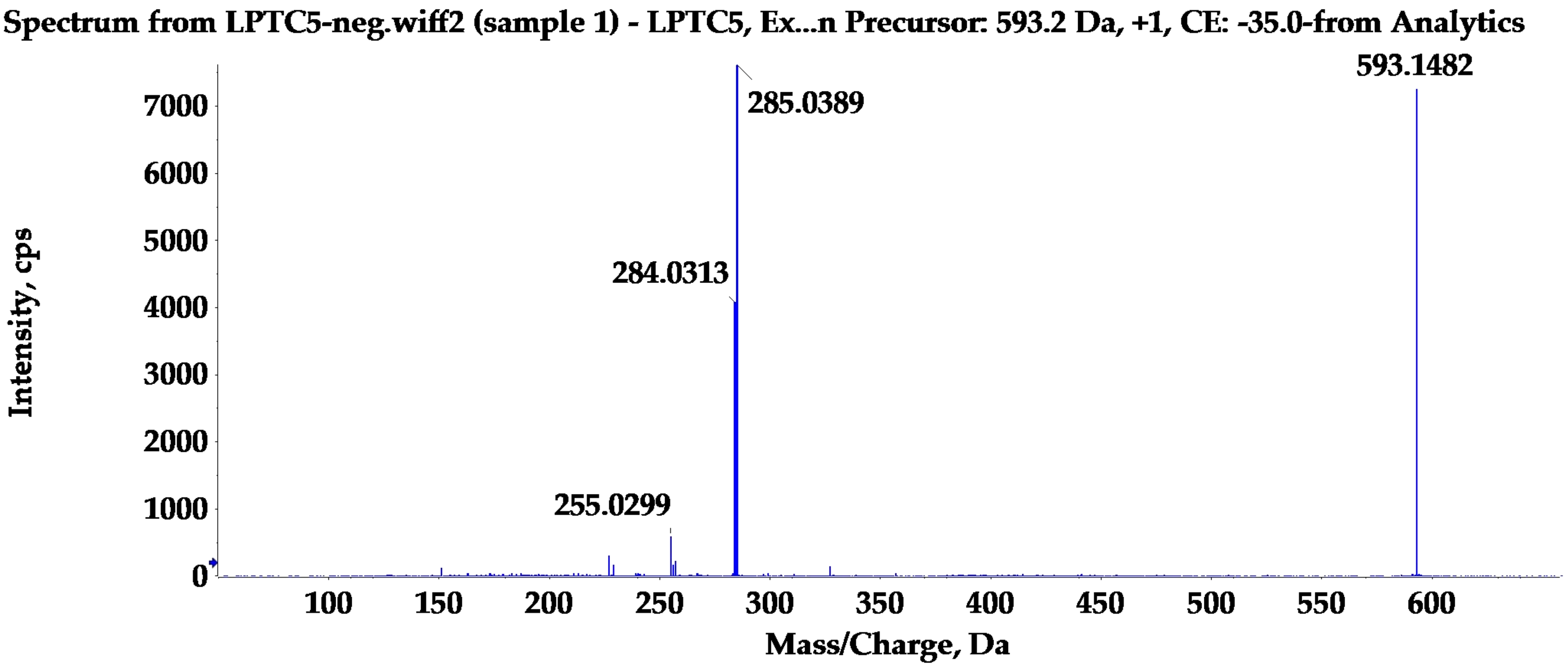

Calycosin-7-O-β-D-glucoside

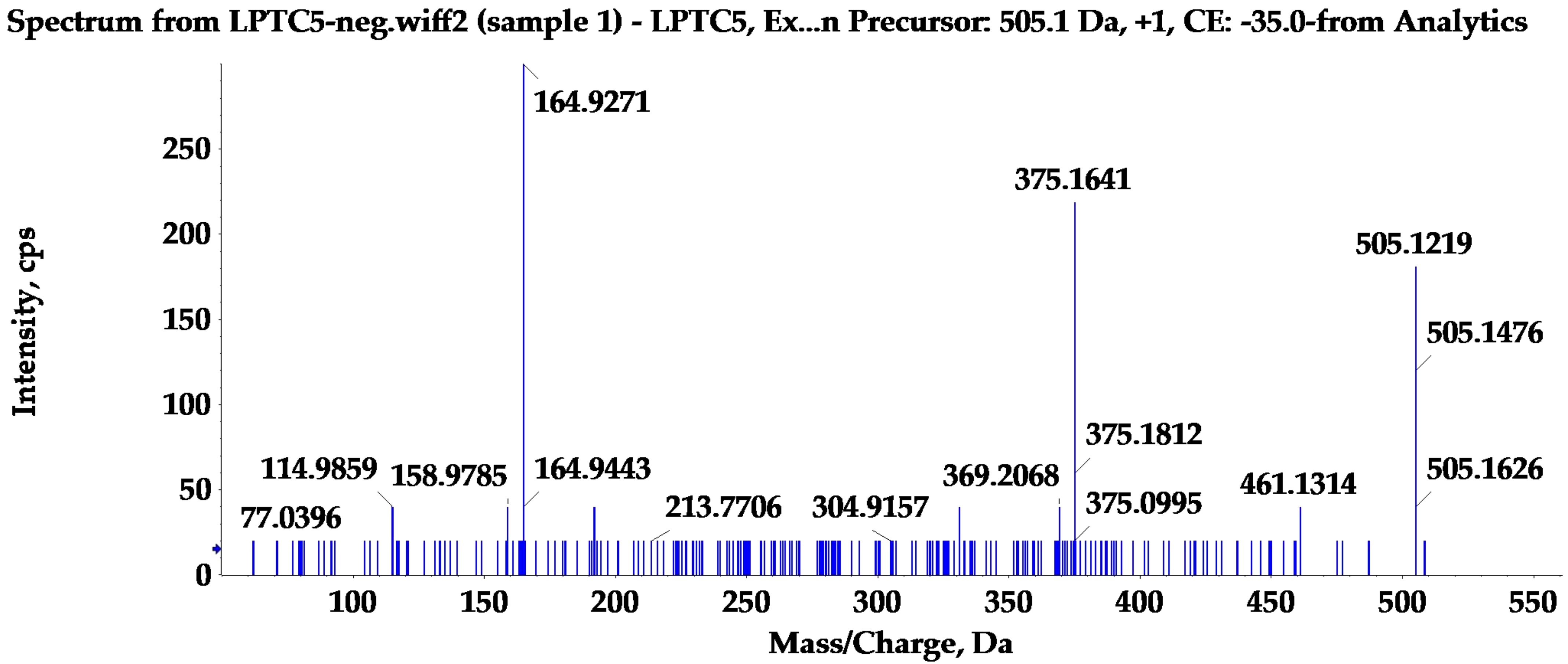

Raspberry Ketone

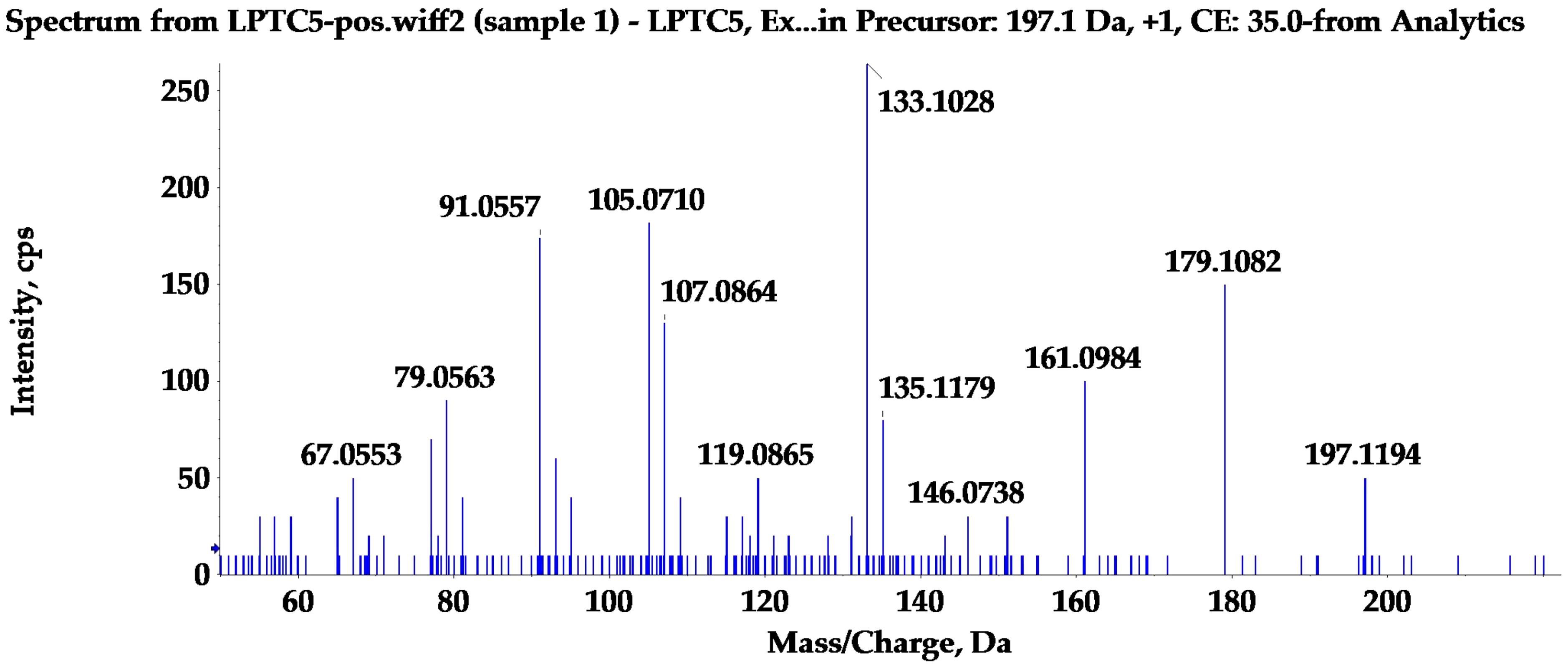

Supplement: Supplementary file 1 [file pharmaceuticals-18-00294-s001.zip › pharmaceuticals-3442841-supplementary/Supplementary Material S2-Ion fragment information.pdf]
